# Selective synthesis of spirobiindanes, alkenyl chlorides, and monofluoroalkenes from unactivated *gem*-difluoroalkanes controlled by aluminum-based Lewis acids

**DOI:** 10.1038/s41598-019-55206-7

**Published:** 2019-12-13

**Authors:** Jiandong Wang, Yuta Ogawa, Norio Shibata

**Affiliations:** 1 Department of Nanopharmaceutical Sciences and Department of Life Science and Applied Chemistry, Nagoya Institute of Technology, Gokiso, Showa-ku, Nagoya 466-5888 Japan; 2 Institute of Advanced Fluorine-Containing Materials, Zhejiang Normal University, 688 Yingbin Avenue, 321004 Jinhua, China

**Keywords:** Synthetic chemistry methodology, Reactive precursors

## Abstract

The highly selective synthesis of spirobiindanes, alkenyl chlorides, and monofluoroalkenes via the cleavage of inert C(sp^3^)–F bonds in unactivated *gem*-difluoroalkanes using readily available and inexpensive aluminum-based Lewis acids of low toxicity is reported. The selectivity of this reaction can be controlled by modifying the substituents on the central aluminum atom of the promoter. An intramolecular cascade Friedel-Crafts alkylation of unactivated *gem*-difluorocarbons can be achieved using a stoichiometric amount of AlCl_3_. The subsequent synthesis of alkenyl chlorides via F/Cl exchange followed by an elimination can be accomplished using AlEt_2_Cl as a fluoride scavenger and halogen source. The defluorinative elimination of acyclic and cyclic *gem*-difluorocarbons to give monofluoroalkenes can be achieved using AlEt_3_.

## Introduction

The widespread use of a variety of readily available organofluorine molecules in the chemical industry and the environmental concerns caused by the longevity of some potentially toxic fluorinated organic compounds has inspired impressive advances of the defluorinative functionalization of carbon-fluorine (C–F) bond^1–6^. In contrast to the considerable number of reports that focus on the transition-metal-mediated or -catalyzed cleavage of C(sp^2^)–F bonds in aromatic and vinylic fluorocarbons^2–4,6^, the direct degradation of C(sp^3^)–F bonds in unactivated aliphatic fluorides remains challenging^7,8^.

**Figure 1:**
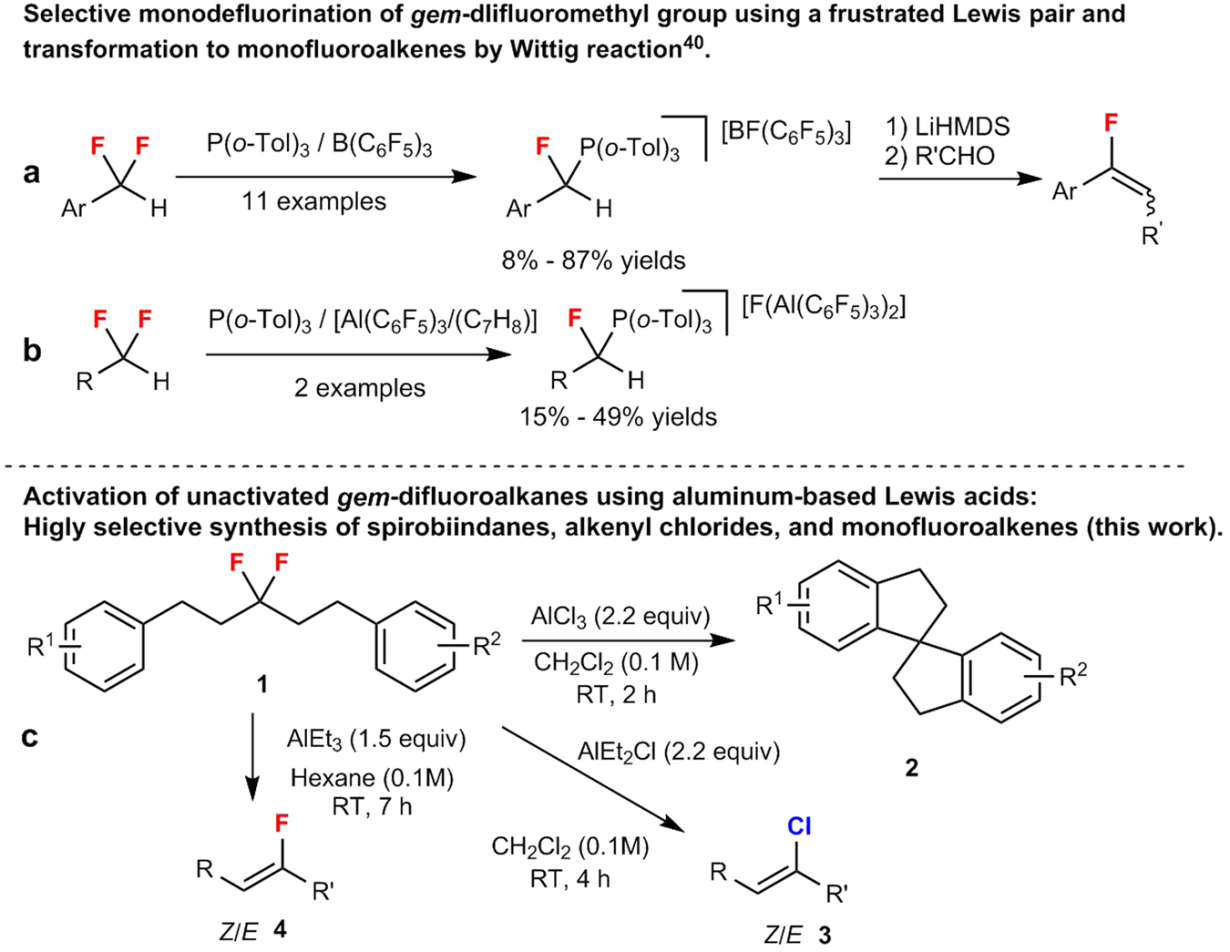
Selective defluorination of unactivated *gem*-difluoroalkanes. (**a,b**) *gem*-Difluoro substrates for C–F bond activation (previous work). (**c**) Selective transformation of *gem*-difluoro substrates controlled by aluminum reagents (this work).

Main-group-based Lewis acids that promote fluoride-abstraction processes have dramatically emerged in recent decades as an attractive strategy to selective functionalize inert C(sp^3^)–F bonds^7,9–12^. Although the fluoride moiety in C–F bonds is neither a good leaving group nor a good Lewis base^13,14^, the formation of more stable covalent bonds (e.g. Si–F, B–F, Al–F, and P–F) provides in many cases the thermodynamic driving force for this heterolytic transformation^7^. In particular, practical and economic protocols that render the scission of the C(sp^3^)–F bond feasible include aluminum-based Lewis acids such as aluminum halides, AlEtCl_2_, AlEt_2_Cl, Al(alkyl)_3_, Al(O*i*-Pr)_3_, or alumina, which are inexpensive, easy to handle, environmentally benign, and commonly used aluminum reagents of low toxicity^15–17^. In 1938, Henne and Newman reported the fluorine/chlorine (F/Cl) exchange between trifluoromethyl benzene and aluminum chloride^18^, while the pioneering work of C–F bond activation appeared in the report by Olah and co-workers in 1957, the ionization of C-F bond to synthesize long lived carbocations^19^. Not only boron based Lewis acids, antimony, bismuth, arsenic based Lewis acids and silica surface also effectively activate C–F bonds^20–25^. Inspired by these work, using aluminum-based Lewis acids, a wide range of transformations of saturated fluorocarbons, including hydrodefluorinations, halodefluorinations, Friedel-Crafts alkylations, and the formation of C-heteroatom bonds^5,7,8^ have been studied extensively. However, most of these reports have focused on activated fluoroalkane substrates for further modifications such as benzylic and allylic trifluoroalkanes^26–29^, as well as benzylic and tertiary aliphatic monofluoroalkanes^30,31^, all of which afford stabilized carbocation intermediates. Meanwhile, due to their comparatively lower steric congestion, primary monofluoroalkanes have been used for Finkelstein-S_N_2-type halogen-exchange reactions^32,33^. However, in spite of recent advances in transition-metal-catalyzed reactions of activated allylic or propargylic *gem*-difluoroalkanes^34,35^, there are only a few synthetic methods that use classical aluminum-based Lewis acids on *gem*-difluorocarbon-type substrates. In early examples, the alkylation and chlorodefluorination of benzylic *gem*-difluorocarbons has been achieved using an excess of AlCl_3_, AlMe_3_, or AlPh_3_^28^. Subsequently, the S_N_2′-type alkylation of difluorohomoallyl alcohols can be controlled by trialkylaluminum compounds, which can coordinate to fluorine and adjacent oxygen atoms^36,37^. Recently, it has been reported that Al(OTf)_3_ enables the defluorinative cycloaddition/aromatization between benzylic 2,2-difluoroethanol and nitriles to afford oxazoles^38^. Nevertheless, breaking C(sp^3^)–F bonds in unactivated *gem*-difluoroalkanes remains highly challenging^39–41^. In 2018, Young and co-workers achieved the selective monodefluorination of benzylic and non-benzylic *gem*-difluoromethyl compounds using a frustrated Lewis pair approach based on B(C_6_F_5_)_3_ and P(*o*-Tol)_3_ to generate monofluoro phosphonium salts, which were subsequently convert into monofluoroolefins using Wittig protocols (Fig. 1a). Although the activation of benzylic *gem*-difluoromethyl groups proceeds in good yields (Fig. 1a), the abstraction of fluoride from unactivated 1,1-difluoroalkanes does not proceed well, and the more fluorophilic Lewis acid [Al(C_6_F_5_)_3_·(C_7_H_8_)] (2 equiv.) was required for the transformation, which proceeded in lower yields (Fig. 1b)^40^.

The occurrence of “over reactions” and poor reaction selectivity, which are mainly caused by unexpected transformations^32,39^ that include hydride shifts, hydrogen fluoride (HF) eliminations, and skeletal rearrangements of unstable fluoro-substituted carbocation intermediates generated from the initial abstraction of fluoride from a *gem*-difluoromethyl moiety, renders controlled synthetic methods highly desirable. Recently, we have reported the selective synthesis of spirobiindanes and monofluoroalkenes using B(C_6_F_5_)_3_ and hexafluoroisopropanol (HFIP), which exhibits a very high affinity toward fluoride^41^. Although the method is of great importance as a proof-of-concept, the reaction still requires high temperatures and the relatively expensive reagents B(C_6_F_5_)_3_ and HFIP, which are critical for this transformation. Our continued interest in the activation and modification of inert C(sp^3^)–F bonds^41,42^ has led us to examine ubiquitous aluminum-based Lewis acids of low cost for the selective synthesis of spirobiindanes (**2**), alkenyl chlorides (**3**), and monofluoroalkenes (**4**) from unactivated *gem*-difluoroalkanes (**1**) under mild conditions. Specifically, we used stoichiometric amounts of AlCl_3_, AlEt_2_Cl, or AlEt_3_ in this study to induce aluminum-fluorine (Al–F) interactions^29,43,44^ for the direct abstraction of fluoride (Fig. 1c).

## Results

### Optimization study

The results of the screening of Al-based Lewis acids for the cleavage of C(sp^3^)–F bonds are summarized in Table 1. Initially, we selected the simple unactivated aliphatic difluoroalkane 3,3-difluoropentane-1,5-diyl)dibenzene (**1a**) as a substrate. When 2.2 equiv. of AlCl_3_ was used to initiate an intramolecular Friedel-Crafts cyclizations, the targeted 2,2′,3,3′-tetrahydro-1,1′-spirobi[indene] (**2a**) was formed in 72% yield (Table 1, entry 1), albeit under heterogeneous conditions. Attempts to render the reaction catalytic were unsuccessful, i.e., the formation of **2a** was observed in <10% yield when 0.2 equiv. of AlCl_3_ were used (entry 3). However, when 1.1 equiv. of AlCl_3_ were used for the degradation of fluorinated **1a**, the defluorinative chlorination/elimination product (3-chloropent-2-ene-1,5-diyl)dibenzene (**3a**) was formed in 24% yield, together with **2a** in 39% yield (entry 2). As alkenyl chlorides represent useful building blocks for the formation of complex organic architectures^45–48^, establishing control by preventing such “over reactions” in favor of alkenyl chlorides **3** would most likely be as attractive as it would be challenging. To solve this problem, we aimed at decelerating the heterolysis of C(sp^3^)–F bonds in *gem*-difluoroalkanes **1** by tuning the Lewis acidity of the aluminum reagents, which could potentially establish control over the reaction selectivity and exclusively afford alkenyl chlorides **3**. Therefore, we focused our attention on organoaluminum reagents with reduced Lewis acidity by adding electron-rich alkyl substituents to the central aluminum atom (entries 4–7).

**Table 1:** Optimization of the reaction conditions with respect to Al-based Lewis acids.


Entry	Lewis acids (equiv)	Time (h)	Product^a^ (%)		
			2a	3a	4a
1	AlCl_3_ (2.2)	2	72	ND	ND
2	AlCl_3_ (1.1)	8	39	24	ND
3	AlCl_3_ (0.2)	8	6	trace	ND
4	AlEtCl_2_ (2.2)	8	complex mixture	—	—
5	AlEt_2_Cl (2.2)	4	ND	92^b^	ND
6	AlEt_3_ (2.2)	<0.5	ND	—	51^c^
7	AlEt_3_ (1.5)	7	ND	—	85^d^

^a^Isolated yields for **2a** and **3a**. ^19^F NMR yield for **4a** using trifluorotoluene as the internal standard. ND = not detected by ^1^H or ^19^F NMR analysis of the crude reaction mixture. ^b^*Z*/*E* = 12:1. ^c^*Z*/*E* = 7.3:1. ^d^Hexane was used as reaction solvent (0.1 M), *Z/E* = 8.7:1.

Recently, it has been reported that an equimolar amount of AlEtCl_2_ promotes an intermolecular S_N_1′-type substitution in 2-trifluoromethyl-1-alkenes^29^. However, when we treated **1a** with 2.2 equiv. of AlEtCl_2_, we obtained only a tar-like complex mixture (entry 4). Yet, when using the weaker Lewis acid AlEt_2_Cl, alkenyl chlorides **3** formed exclusively, i.e., the desired **3a** was obtained in 92% yield and the formation of side products was not observed (entry 5; for more details, see also Supplementary Fig. 76 in SI). AlEt_2_Cl has already been reported to facilitate F/Cl exchange reactions in aliphatic monofluoroalkanes at −78 °C via S_N_1- or S_N_2-type mechanisms, albeit that these reactions exhibit a very limited substrate scope^32^. Using AlEt_3_ under otherwise identical reaction conditions afforded monofluoroalkene **4a** in 51% yield without producing any Friedel-Crafts alkylation products (**2a**). Further improvement of the yield of **4a** to 85% was observed upon conducting the reaction in *n*-hexane, using 1.5 equiv. of AlEt_3_, and prolonging the reaction time (entry 7; for more details, see also Supplementary Table 1 in SI). However, it should be noted here that the AlEt_3_-mediated defluorinative elimination of 1,1-difluorocyclopentane has already been reported by Ozerov, albeit only in one special case^39^. Specifically, the formal HF-abstraction product 1-fluorocyclopent-1-ene was observed in 24% ^19^F NMR yield after a C_6_D_12_ solution of 1,1-difluorocyclopentane (1.5 M) in a J. Young tube had been treated for 24 h with AlEt_3_ (2.0 equiv.) at room temperature^39^. Modifying the substituents on the central aluminum atom (AlCl_3_, AlEt_2_Cl, and AlEt_3_) allowed tuning the reaction selectivity for the heterolysis of the C(sp^3^)–F bonds in unactivated *gem*-difluoroalkanes **1**.

### Substrate scope

**Figure 2:**
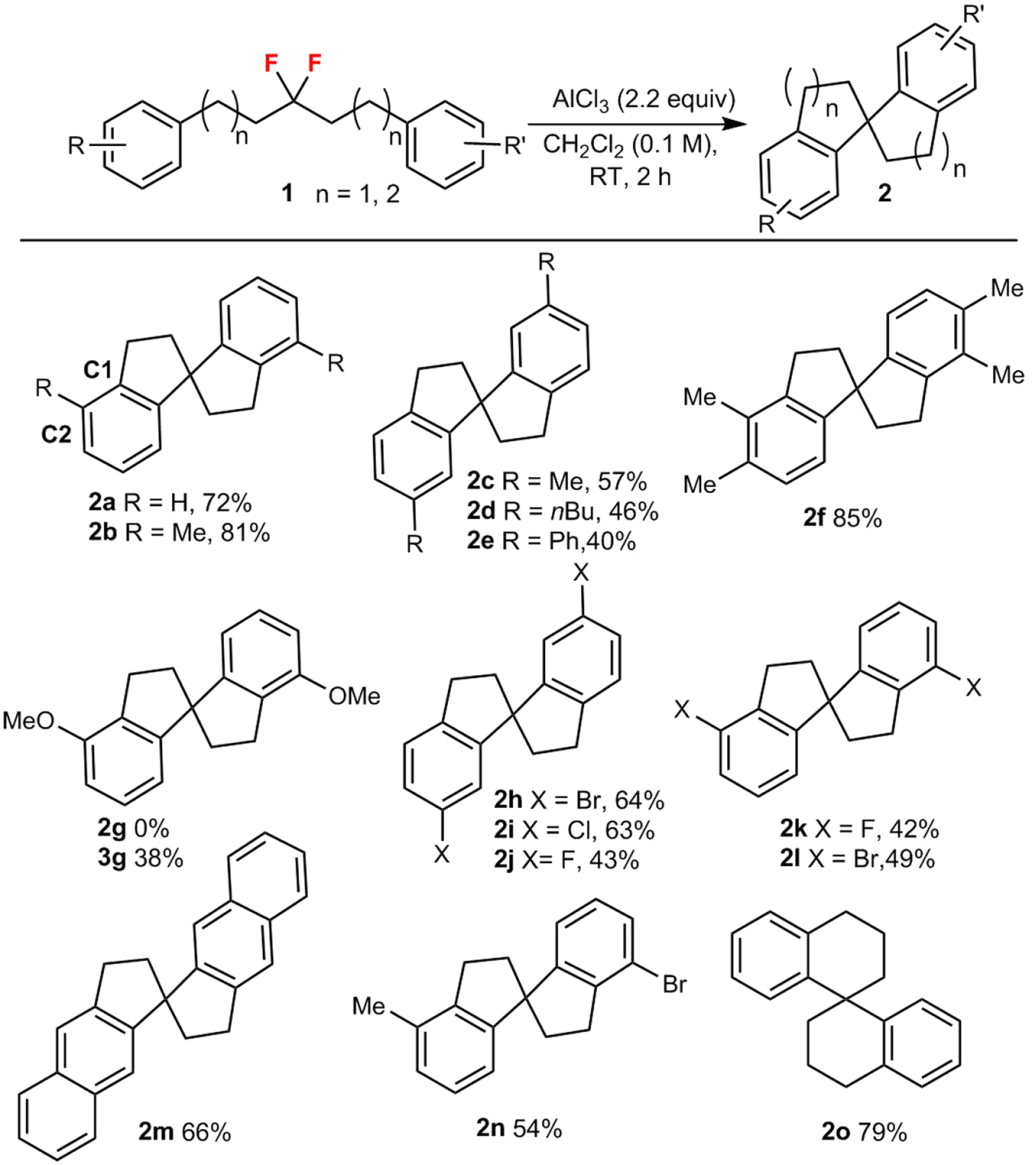
AlCl_3_-mediated synthesis of spirobiindanes 2.

As shown in Fig. 2, aliphatic *gem*-difluoroalkanes substituted with alkyl groups (**1a**–**f**) afford moderate to high yields (up to 85%) of the corresponding spirobiindanes, whereby C2-substituted substrates (**1a**,**b**) perform slightly better than C4-substituted substrates (**1c**–**e**). Interestingly, when using methoxy-substituted *gem*-difluoride **1 g**, alkenyl chloride 2,2′-(3-chloropent-2-ene-1,5-diyl)bis(methoxybenzene) (**3 g**) was formed in 38% yield, and the desired Friedel-Crafts alkylation product (**2 g**) was not observed. Consistent with our strategy that a modification of the Lewis acidity could potentially control the reaction selectivity, the oxygen atom in **1 g** probably coordinates to the aluminum center of AlCl_3_ and thus reduces its Lewis acidity, which would hamper the fluoride-abstraction process, and thus switch the reaction pathway from the expected Friedel-Crafts alkylation to a chlorination/elimination process. The presence of halogen substituents in the *gem*-difluoroalkanes (**1h**–**l**) was well tolerated when using AlCl_3_, and the corresponding products were generated in acceptable yield (42–64%). Moreover, naphthyl-type **2 m**, mixed product **2n**, and the six-membered spiro-compound **2o** were also obtained in good yield.

**Figure 3:**
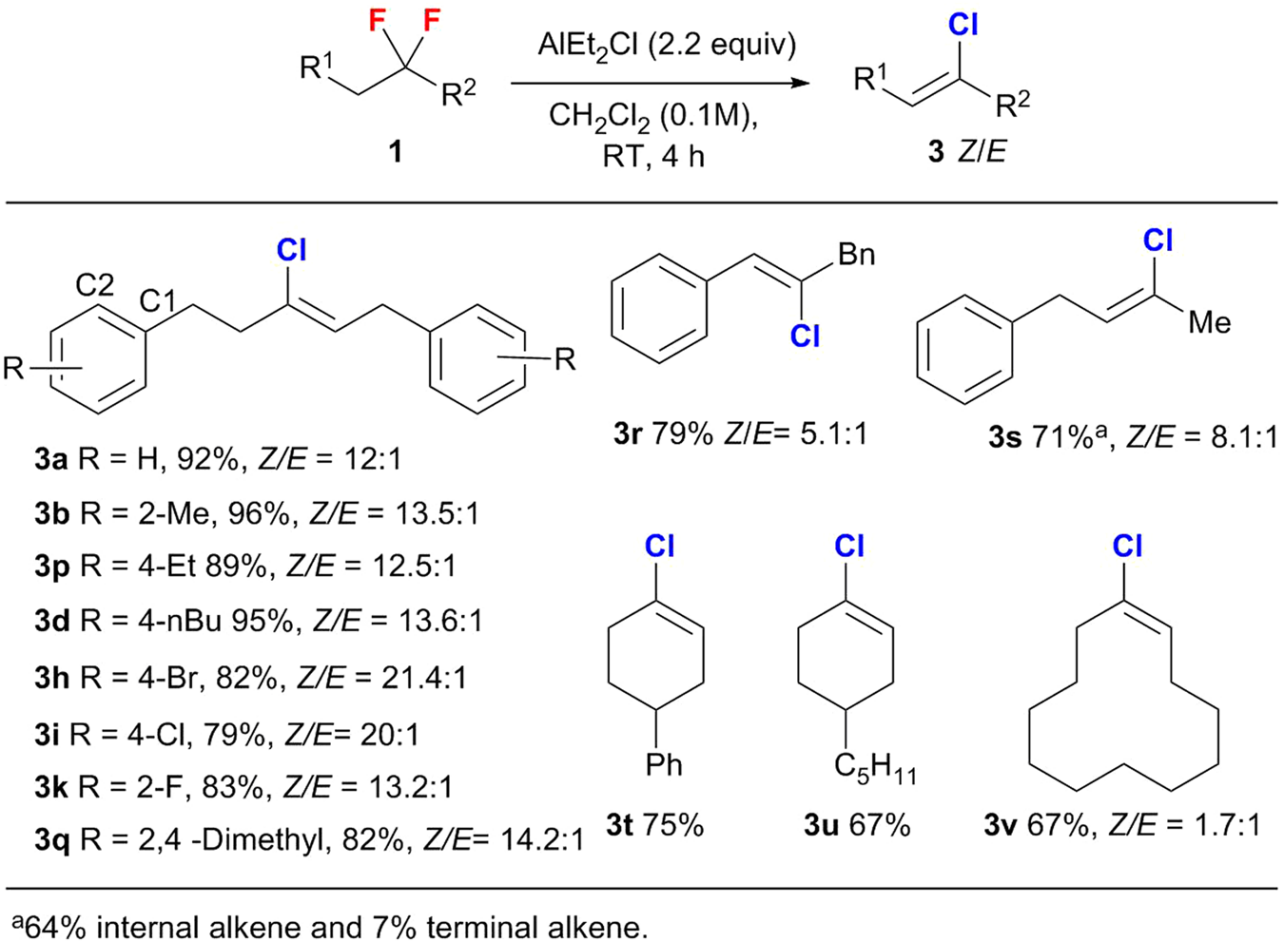
AlEt_2_Cl-mediated synthesis of trisubstituted alkenyl chlorides 3.

Subsequently, we examined the synthesis of tri-substituted alkenyl chlorides (**3**) using AlEt_2_Cl both as an activator and a chloro source (Fig. 3). High yields and good *Z*/*E* stereocontrol were observed in most cases; specifically, long-chain acyclic substrates, independent of their substitution pattern on the benzene ring, afforded the desired alkenyl chlorides (**3a**,**b**, **3p**, **3d**, **3h**–**k**, **3q**), including halogen-substituted products, in good to high yield (up to 96%) with good *Z*/*E* stereoselectivity (up to 21.4:1). In addition, moderate regioselectivity was observed for the defluorinative chlorination/elimination to provide the inner alkene product (3-chlorobut-2-en-1-yl)benzene (**3 s**), and only ~10% of the corresponding terminal alkene was formed. It should also be noted here that cyclic *gem*-difluoroalkanes (**1t**–**v**) are also well tolerated under the AlEt_2_Cl-mediated conditions, furnishing the targeted cyclic alkenyl chlorides (**3t**–**3v**) in acceptable yield (67–75%).

**Figure 4:**
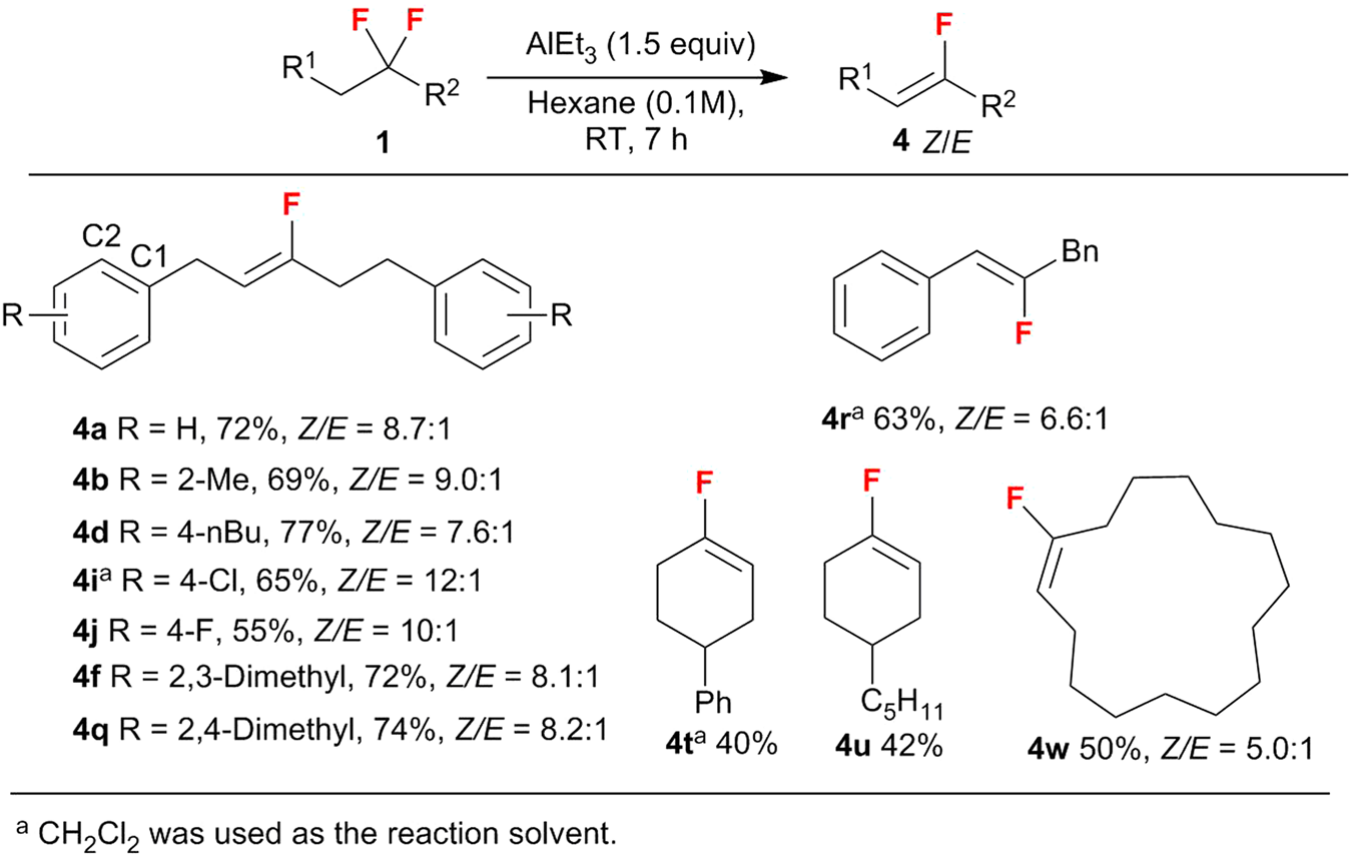
AlEt_3_-mediated synthesis of monofluoroalkenes 4.

Monofluoroalkenes **4** were obtained via a defluorination/elimination process (Fig. 4). As expected, long-chain acyclic substrates, independent of their substitution pattern on the benzene ring, furnished the desired monofluoroalkenes (**4a**-**b**, **4d**, **4i**,**j**, **4f**, and **4q** in moderate to good yield (up to 77%) with good *Z*/*E* stereocontrol (up to 12:1). In particular, dialkyl-substituted substrates **1 f** and **1q** generated the corresponding monofluoroalkenes (**4 f** and **4q**) in 72% and 74% yield, respectively. Furthermore, the defluorination of cyclic substrates including large-ring-type *gem*-difluoroalkanes proceeded smoothly to afford the corresponding cyclic monofluoroalkenes (**4t**, **4 u**, and **4w**) in moderate yield (40–50%)^39,49,50^.

### Mechanistic investigations

**Figure 5:**
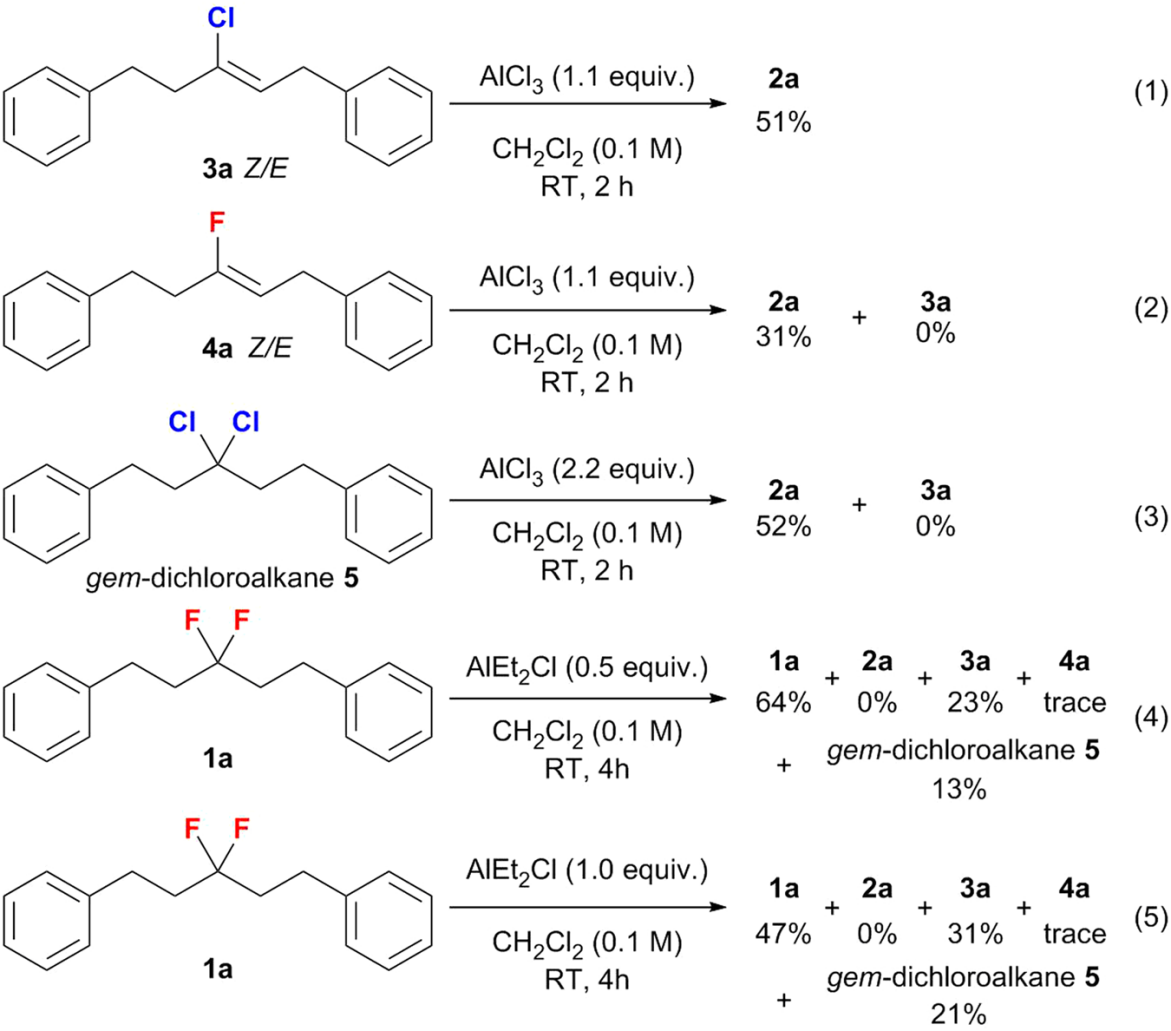
Control experiments to investigate possible reaction intermediates (percentage values refer to the NMR yield).

**Figure 6:**
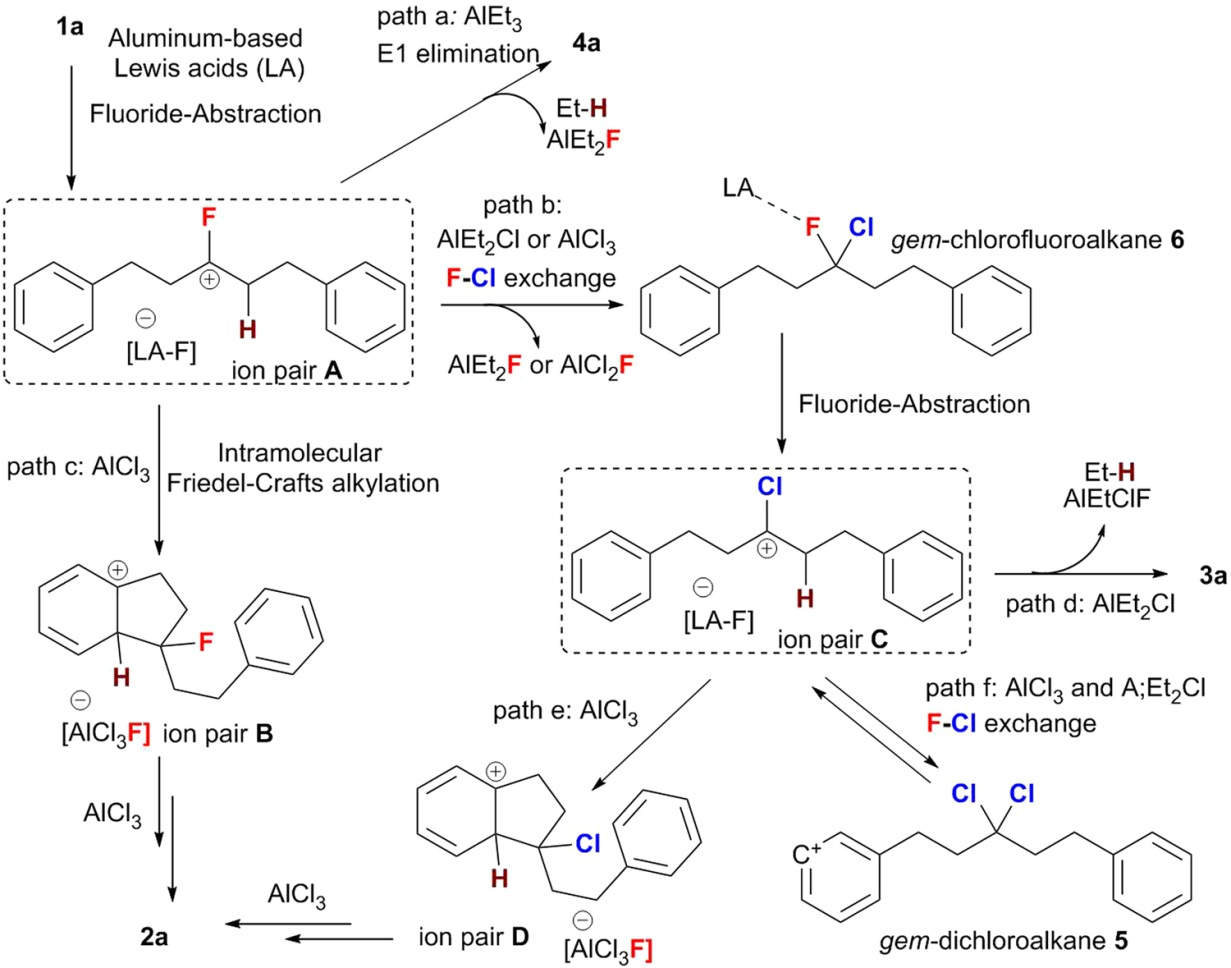
Proposed reaction mechanism.

In order to avoid “over reactions” during the modification of inert C(sp^3^)-F bonds in saturated *gem*-difluoroalkanes (**1**), we reduced the Lewis acidity of the Al-based promoters. We observed that such “controllable reactions” stopped at the defluorinative elimination or F-Cl exchange/elimination stage, while further Friedel-Crafts alkylations did not occur. Indeed, using methoxyl-substituted fluorocarbon **1 g** and AlCl_3_ (Fig. 2) represents a special case, as it does not generate the desired spiro product **2 g**, but alkenyl chloride **3 g** in 38% yield. Accordingly, the vital importance of the Lewis acidity of the aluminum promotors for the reaction selectivity can feasibly be rationalized under consideration of two points: 1. The abstraction of a fluoride anion from the C(sp^3^)–F bonds is facilitated with increasing strength of the Lewis acidity of the main-group promoter, as the fluorine moiety is neither a good Lewis base nor a good leaving group^13^. Indeed, species with a stronger formal positive charge such as [Ph_3_C]^+^, [R_3_Si]^+^, [R_2_Al]^+^, [(C_6_F_5_)_3_PF]^+^, and even P(III) dications with weakly coordinating anions, have recently been used for the direct cleavage and functionalization of C(sp^3^)–F bonds^10,12,51–53^. 2. Weaker Lewis acids favor elimination over substitution reactions of carbocation intermediates, which is due to the higher Lewis basicity of the conjugated Lewis bases [LA–F]^−^ (LA = Lewis acid) generated form the heterolytic cleavage of the C–F bonds. Thus, the weaker LA AlEt_3_ afforded only monofluoroalkenes **4** via a defluorinative elimination, commensurate with the formal loss of one molecule of HF. Although proposing a clear mechanism is difficult due to the potential complexity of the structures of the conjugated Lewis bases [LA–F]^−^, which may form fluoride-bridged polymetric framework^54–57^, as well as due to the heterogeneous reaction conditions when using aluminum trichloride^58,59^, control experiments were conducted (Fig. 5) and a feasible reaction mechanism that would explain the high reaction selectivity is outlined in Fig. 6.

Initially, the strong Al-F interaction could promote the cleavage of one C(sp^3^)–F bond in *gem*-difluoroalkanes to give one tight ion pair (**A**) between a fluorinated carbocation and a conjugated Lewis base [LA–F]^−^ counter ion. Then, the reaction could proceed via three competitive reaction pathways: 1. Direct elimination of the acidic α-proton of the fluorinated carbocation intermediate to give monofluoroalkenes **4**, which is favored in the presence of AlEt_3_; 2. Twofold intramolecular Friedel-Crafts alkylation in the presence of AlCl_3_; 3. F-Cl exchange reaction via S_N_1-type substitutions^32^ in the presence of AlCl_3_ or AlEt_2_Cl. Subsequently, the second abstraction of a fluoride anion could generate the tight ion pair **C**, which bears a chlorinated carbocation. In a similar fashion, the direct Friedel-Crafts alkylation, the F-Cl exchange, and the E1-type elimination represent three competitive reaction pathways. As mentioned above, when 1.1 equiv. of AlCl_3_ were used, the trisubstituted alkenyl chloride was detected in 24% yield (Table 1, entry 2). Using alkenyl chloride **3a** as the chlorinated carbocation precursor furnished the intramolecular Friedel-Crafts type product **2a** in 51% yield in the presence of 1.1 equiv. of AlCl_3_, while 31% yield were observed when using monofluorinated olefin **4a** as the precursor for the fluorinated carbocation intermediate under otherwise identical reaction conditions (Fig. 5). Meanwhile, the F-Cl-exchange-type product 3,3-dichloropentane-1,5-diyl)dibenzene (**5**) also generated spirobiindane **2a** in 52% yield. These results indicate that the cascade intramolecular Friedel-Crafts cyclization is a complex transformation that involves fluorinated carbocations and chlorinated carbocation intermediates, as well as competitive F-Cl exchange reaction pathways. Although we were unable to capture any F-Cl exchange products such as *gem*-chlorofluoroalkane **6** when using 0.5 equiv. or 1.0 equiv. of AlEt_2_Cl, the double F-Cl exchange product *gem*-dichloroalkane **5** was observed in 13% and 21% yield, respectively (Fig. 5; for more details, see the NMR study in Supplementary Figs. 78–85 in SI). Thus, alkenyl chloride **3** is probably generated from the double F-Cl exchange product *gem*-dichloroalkane **5**, which may serve as a reservoir for the chlorinated carbocation intermediate in the tight ion pair **C**.

## Discussion

In conclusion, we have developed a highly selective synthetic route to spirobiindanes **2**, trisubstituted alkenyl chlorides **3**, and monofluoroalkenes **4**, based on the aluminum-induced cleavage of inert C(sp^3^)–F bonds in unactivated *gem*-difluoroalkanes **1**. The three reaction types can be selectively controlled by using the readily available aluminum-based Lewis acids AlCl_3_, AlEt_2_Cl, or AlEt_3_. Since the reaction can be performed using ubiquitous and cheap aluminum-based Lewis acids at room temperature, these methods should be of high practical utility.

## Methods

### General procedure for the intramolecular Friedel-Craft reaction of gem-difluoroalkanes

In a flame-dried test tube (10 mL), to the heterogeneous solution of AlCl_3_ (29.3 mg, 0.22 mmol, 2.2 equiv.) in dry CH_2_Cl_2_ (0.5 mL), *gem*-difluoroalkanes **1** (0.1 mmol) in dry CH_2_Cl_2_ (0.5 mL) was added dropwise by syringe, and the reaction mixture was stirred at room temperature for 2 hours under a positive pressure of argon with a balloon. Then, the resulting mixture was washed with water, extracted with CH_2_Cl_2_, dried over Na_2_SO_4_, filtered, and then concentrated *in vacuo*. The residue was purified by column chromatography on silica gel using *n*-hexane as the eluent to afford the desired spirobiindanes **2a-n** and spirobitetraline **2o**. In addition, alkenyl chloride **3 g**, 2,2′-(3-chloropent-2-ene-1,5-diyl)bis(methoxybenzene), was also prepared as one special example. In addition, the *gem*-difluoroalkanes **1** were prepared based on previous reports via fluorination of corresponding ketones by (diethylamino)sulfur trifluoride or 4-*tert-*butyl-2,6-dimethylphenylsulfur trifluoride (Fluolead).

### General procedure for the synthesis of alkenyl chlorides 3 from *gem*-difluoroalkanes 1

In a flame-dried test tube (10 mL), diethylaluminum chloride (255 *μ*L, ca. 0.22 mmol, 2.2 equiv., ca. 15% in hexane, ca. 0.87 mol/L) was added slowly to the solution of *gem*-difluoroalkanes **1** (0.1 mmol) in dry CH_2_Cl_2_ (0.1 M, 1.0 mL), and the reaction mixture was stirred at room temperature for 4 hours under a positive pressure of argon with a balloon. Then, the resulting mixture was washed with water, extracted with CH_2_Cl_2_, dried over Na_2_SO_4_, filtered, and then concentrated *in vacuo*. The residue was purified by column chromatography on silica gel using *n*-hexane as the eluent to afford the desired alkenyl chloride **3**. The ratio for *Z*/*E* isomers was determined by ^1^H NMR based on previous literature.

### General procedure for the synthesis of monofluoroalkene 4 from *gem*-difluoroalkanes 1

In a flame-dried test tube (10 mL), triethylaluminum (150 *μ*L, ca. 0.15 mmol, 1.5 equiv.,15% in hexane, ca. 1.0 mol/L) was added slowly to the solution of *gem*-difluoroalkanes **1** (0.1 mmol) in *n*-hexane (0.1 M, 1.0 mL), and the reaction mixture was stirred at room temperature for 7 hours under a positive pressure of nitrogen with a balloon. Then, the resulting mixture was washed with water, extracted with CH_2_Cl_2_, dried over Na_2_SO_4_, filtered, and then concentrated *in vacuo*. The residue was purified by column chromatography on silica gel to afford the desired monofluoroalkene **4**. The ratio for *Z*/*E* isomers was determined by ^19^F NMR.

## Supplementary information

supplementary information

## Data Availability

The authors declare that all the data supporting the findings of this study are available within the paper and its supplementary information files, and also are available from the corresponding author upon reasonable request.

## References

[1] Amii H, Uneyama K (2009). C−F Bond Activation in Organic Synthesis. Chem. Rev..

[2] Ahrens T, Kohlmann J, Ahrens M, Braun T (2015). Functionalization of Fluorinated Molecules by Transition-Metal-Mediated C–F Bond Activation To Access Fluorinated Building Blocks. Chem. Rev..

[3] Eisenstein O, Milani J, Perutz RN (2017). Selectivity of C–H Activation and Competition between C–H and C–F Bond Activation at Fluorocarbons. Chem. Rev..

[4] Pike SD, Crimmin MR, Chaplin AB (2017). Organometallic chemistry using partially fluorinated benzenes. Chem. Commun..

[5] Hamel J-D, Paquin J-F (2018). Activation of C–F bonds α to C–C multiple bonds. Chem. Commun..

[6] Kuehnel MF, Lentz D, Braun T (2013). Synthesis of Fluorinated Building Blocks by Transition-Metal-Mediated Hydrodefluorination Reactions. Angew. Chem. Int. Ed..

[7] Stahl T, Klare HFT, Oestreich M (2013). Main-Group Lewis Acids for C−F Bond Activation. ACS Catal..

[8] Shen Q (2015). Review of recent advances in C−F bond activation of aliphatic fluorides. J. Fluorine Chem..

[9] Caputo CB, Stephan DW (2012). Activation of Alkyl C–F Bonds by B(C_6_F_5_)_3_: Stoichiometric and Catalytic Transformations. Organometallics.

[10] Chitnis SS (2018). Phosphorus Coordination Chemistry in Catalysis: Air Stable P(III)-Dications as Lewis Acid Catalysts for the Allylation of C–F Bonds. Organometallics.

[11] Bamford KL, Chitnis SS, Qu Z-W, Stephan DW (2018). Interactions of C−F Bonds with Hydridoboranes: Reduction, Borylation and Friedel-Crafts Alkylation. Chem.-Eur. J..

[12] Douvris C, Ozerov OV (2008). Hydrodefluorination of Perfluoroalkyl Groups Using Silylium-Carborane Catalysts. Science.

[13] O’Hagan D (2008). Understanding organofluorine chemistry. An introduction to the C–F bond. Chem. Soc. Rev..

[14] Nolte C, Ammer J, Mayr H (2012). Nucleofugality and Nucleophilicity of Fluoride in Protic Solvents. J. Org. Chem..

[15] Maruoka K, Yamamoto H (1985). Selective Reactions Using Organoaluminum Reagents [New Synthetic Methods (54)]. Angew. Chem. Int. Ed..

[16] Nikonov GI (2017). New Tricks for an Old Dog: Aluminum Compounds as Catalysts in Reduction Chemistry. ACS Catal..

[17] Amsharov KY, Kabdulov MA, Jansen M (2012). Facile Bucky‐Bowl Synthesis by Regiospecific Cove‐Region Closure by HF Elimination. Angew. Chem. Int. Ed..

[18] Henne AL, Newman MS (1938). The Action of Aluminum Chloride on Fluorinated Compounds. J. Am. Chem. Soc..

[19] Olah, G. A., Kuhn S. & Olah, J. 416. Aromatic substitution. Part III. Alkylation of aromatic compounds by the boron trifluoride-catalysed reaction of alkyl fluorides. *J. Chem. Soc*., 2174–2176 (1957).

[20] Olah GA, Kuhn SJ, Barnes DG (1964). Selective Friedel-Crafts Reactions. I. Boron Halide Catalyzed Haloalkylation of Benzene and Alkylbenzenes with Fluorohaloalkanes. J. Org. Chem..

[21] Olah GA, Mo YK (1972). Organic fluorine compounds. XXXIII. Electrophilic additions to fluoro olefins in superacids. J. Org. Chem..

[22] Ichikawa, J., Jyono, H., Kudo, T., Fujiwara, M. & Yokota, M. Friedel-Crafts Cyclization of 1,1-Difluoroalk-1-enes: Synthesis of Benzene-Fused Cyclic Ketones via α-Fluorocarbocations. *Synthesis*, 39–46 (2005).

[23] Betterley NM (2018). Bi(OTf)_3_ Enabled C–F Bond Cleavage in HFIP: Electrophilic Aromatic Formylation with Difluoro(phenylsulfanyl)methane. Asian J. Org. Chem..

[24] Christe KO, Wilson WW, Schack CJ, Wilson RD (1985). Lewis acid induced intramolecular redox reactions of difluoroamino compounds. Inorg. Chem..

[25] Culver DB, Conley MP (2018). Activation of C−F Bonds by Electrophilic Organosilicon Sites Supported on Sulfated Zirconia. Angew. Chem. Int. Ed..

[26] Riera J, Castañer J, Carilla J, Robert A (1989). New synthesis of polychloro(trifluoromethyl)benzenes and highly strained polychloro(trichloromethyl)benzenes. Tetrahedron Lett..

[27] Ramchandani RK, Wakharkar RD, Sudalai A (1996). AlCl_3_-Catalyzed regiospecific alkylation of aromatics with chlorobenzotrifluorides: A high yield preparation of 1,1-dichlorodiphenylmethanes. Tetrahedron Lett..

[28] Terao, J., Nakamura M. & Kambe, N. Non-catalytic conversion of C–F bonds of benzotrifluorides to C–C bonds using organoaluminium reagents. *Chem. Commun*., 6011–6013 (2009). 10.1039/b915620h 19809627

[29] Fuchibe K, Hatta H, Oh K, Oki R, Ichikawa J (2017). Lewis Acid Promoted Single C–F Bond Activation of the CF_3_ Group: S_N_1′-Type 3,3-Difluoroallylation of Arenes with 2-Trifluoromethyl-1-alkenes. Angew. Chem. Int. Ed..

[30] Ooi T, Uraguchi D, Kagashima N, Maruoka K (1997). Organoaluminum-catalyzed new alkylation of tert-alkyl fluorides: Synthetic utility of Al–F interaction. Tetrahedron Lett..

[31] Jaiswal AK, Goh KKK, Sung S, Young RD (2017). Aluminum-Catalyzed Cross-Coupling of Silylalkynes with Aliphatic C–F Bonds. Org. Lett..

[32] Terao, J. *et al*. Conversion of a (sp^3^)C–F bond of alkyl fluorides to (sp^3^)C–X (X = Cl, C, H, O, S, Se, Te, N) bonds using organoaluminium reagents. *Chem. Commun*., 855–857 (2007). 10.1039/b613641a 17308654

[33] Mizukami Y, Song Z, Takahashi T (2015). Halogen Exchange Reaction of Aliphatic Fluorine Compounds with Organic Halides as Halogen Source. Org. Lett..

[34] Drouin M, Hamel J-D, Paquin J-F (2016). Exploiting 3,3-Difluoropropenes for the Synthesis of Monofluoroalkenes. Synlett.

[35] Wang C-Q, Ye L, Feng C, Loh T-P (2017). C–F Bond Cleavage Enabled Redox-Neutral [4+1] Annulation via C–H Bond Activation. J. Am. Chem. Soc..

[36] Yanai H, Okada H, Sato A, Okada M, Taguchi T (2011). Copper-free defluorinative alkylation of allylic difluorides through Lewis acid-mediated C–F bond activation. Tetrahedron Lett..

[37] Sato A, Yanai H, Suzuki D, Okada M, Taguchi T (2015). Synthesis of (*Z*)-fluoroallyl azides through aluminium-mediated defluorinative functionalization reactions. Tetrahedron Lett..

[38] Hsieh M-T, Lee K-H, Kuo S-C, Lin H-C (2018). Lewis acid-mediated defluorinative [3+2] cycloaddition/aromatization cascade of 2,2-difluoroethanol systems with nitriles. Adv. Synth. Catal..

[39] Gu W, Haneline MR, Douvris C, Ozerov OV (2009). Carbon−Carbon Coupling of C(sp^3^)−F Bonds Using Alumenium Catalysis. J. Am. Chem. Soc..

[40] Mandal D, Gupta R, Young RD (2018). Selective Monodefluorination and Wittig Functionalization of *gem*-Difluoromethyl Groups to Generate Monofluoroalkenes. J. Am. Chem. Soc..

[41] Wang J, Ogawa Y, Shibata N (2019). Activation of Saturated Fluorocarbons to Synthesize Spirobiindanes, Monofluoroalkenes, and Indane derivatives. iScience.

[42] Haufe G (2012). C−F Bond Activation of Unactivated Aliphatic Fluorides: Synthesis of Fluoromethyl-3,5-diaryl-2-oxazolidinones by Desymmetrization of 2-Aryl-1,3-difluoro-2-propanols. Angew. Chem. Int. Ed..

[43] Ooi T, Kagoshima N, Uraguchi D, Maruoka K (1998). Organoaluminum-promoted selective addition to fluorinated carbonyl compounds via pentacoordinate trialkylaluminum complexes. Tetrahedron Lett..

[44] Maruoka K, Ooi T (1999). The Synthetic Utility of the Hypercoordination of Boron and Aluminum. Chem.-Eur. J..

[45] Petrone DA, Ye J, Lautens M (2016). Modern Transition-Metal-Catalyzed Carbon–Halogen Bond Formation. Chem. Rev..

[46] Derosa J (2017). Palladium(II)-Catalyzed Directed anti-Hydrochlorination of Unactivated Alkynes with HCl. J. Am. Chem. Soc..

[47] Iwai T, Fujihara T, Terao J, Tsuji Y (2012). Iridium-Catalyzed Addition of Aroyl Chlorides and Aliphatic Acid Chlorides to Terminal Alkynes. J. Am. Chem. Soc..

[48] Zeng X, Liu S, Hammond GB, Xu B (2018). Hydrogen-Bonding-Assisted Brønsted Acid and Gold Catalysis: Access to Both (*E*)- and (*Z*)-1,2-Haloalkenes via Hydrochlorination of Haloalkynes. ACS Catal..

[49] Vandamme M, Paquin J-F (2017). Eliminative Deoxofluorination Using XtalFluor-E: A One-Step Synthesis of Monofluoroalkenes from Cyclohexanone Derivatives. Org. Lett..

[50] Drouin M, Hamel J-D, Paquin J-F (2018). Synthesis of Monofluoroalkenes: A Leap Forward. Synthesis.

[51] Chen J, Chen EY-X (2015). Elusive Silane–Alane Complex [Si—H⋅⋅⋅Al]: Isolation, Characterization, and Multifaceted Frustrated Lewis Pair Type Catalysis. Angew. Chem. Int. Ed..

[52] Zhu J, Pérez M, Caputo CB, Stephan DW (2016). Use of Trifluoromethyl Groups for Catalytic Benzylation and Alkylation with Subsequent Hydrodefluorination. Angew. Chem. Int. Ed..

[53] Forster F, Metsaenen TT, Irran E, Hrobarik P, Oestreich M (2017). Cooperative Al-H Bond Activation in DIBAL-H: Catalytic Generation of an Alumenium-Ion-Like Lewis Acid for Hydrodefluorinative Friedel-Crafts Alkylation. J. Am. Chem. Soc..

[54] Chen M-C, Roberts JAS, Marks TJ (2004). New Mononuclear and Polynuclear Perfluoroarylmetalate Cocatalysts for Stereospecific Olefin Polymerization. Organometallics.

[55] Lehmkuhl H (1964). Complex Formation with Organoaluminum Compounds. Angew. Chem. Int. Ed..

[56] Laubengayer AW, Lengnick GF (1966). The Structure and Properties of Diethylfluoroalane, (C_2_H_5_)_2_AlF. Inorg. Chem..

[57] Dimitrov A, Heidemann D, Kemnitz E (2006). F/Cl-Exchange on AlCl_3_-Pyridine Adducts:  Synthesis and Characterization of trans-Difluoro-tetrakis-pyridine-aluminum-chloride, [AlF_2_(Py)_4_]Cl. Inorg. Chem..

[58] Petrov VA, Krespan CG, Smart BE (1998). Isomerization of halopolyfluoroalkanes by the action of aluminum chlorofluoride. J. Fluorine Chem..

[59] Krahl T (2003). Structural Insights into Aluminum Chlorofluoride (ACF). Inorg. Chem..

