# Supplementary material for: Selective synthesis of spirobiindanes, alkenyl chlorides, and monofluoroalkenes from unactivated gem-difluoroalkanes controlled by aluminum-based Lewis acids
Source: Sci Rep. 2019 Dec 13;9:19113. doi: 10.1038/s41598-019-55206-7 (PMC6911048; doi:10.1038/s41598-019-55206-7)

Supplementary Information

Selective synthesis of spirobiindanes, alkenyl chlorides, and monofluoroalkenes from unactivated *gem*-difluoroalkanes controlled by aluminum-based Lewis acids

Jiandong Wang[1], Yuta Ogawa[1] & Norio Shibata[1, 2]

[1] Department of Nanopharmaceutical Sciences and Department of Life Science and Applied Chemistry, Nagoya Institute of Technology, Gokiso, Showa-ku, Nagoya 466-5888, Japan. (email: nozshiba@nitech.ac.jp)
[2] Institute of Advanced Fluorine-Containing Materials, Zhejiang Normal University, 688 Yingbin Avenue, 321004 Jinhua, China.

General information

All reactions were performed in oven-dried and flame-dried glassware (10 mL) under a positive pressure of argon atmosphere unless mentioned otherwise. Solvents were transferred *via* syringe and were introduced into the reaction vessels through a rubber septum. All of the reactions were monitored by thin-layer chromatography (TLC) carried out on 0.25 mm Merck silica gel (60-F_{254}). The TLC plates were visualized with UV light and 7% phosphomolybdic acid or $KMnO_4$ in ethanol/heat. Column chromatography was carried out on a column packed with silica gel (60N spherical neutral size 50-63 μm). The ^{1}H NMR (300 MHz), ^{19}F NMR (282 MHz), ^{13}C NMR (125 MHz or 75 MHz) spectra for solution in $CDCl_3$ were recorded on a Buruker Avance 500, a Varian Mercury 300 spectrometers. Chemical shifts (δ) are expressed in ppm downfield from internal TMS (δ = 0.00) for ^{1}H NMR. C_6F_6 [δ = −162.2 ($CDCl_3$)] was used as an internal standard for ^{19}F NMR. Mass spectra were recorded on a SHIMADZU GCMS-QP5050A (EI-MS), GCMS-Agilent Technologies 5977A MSD (EI-MS) and SHIMAZU LCMS-2020 (ESI-MS and APCI-MS). High resolution mass spectrometry (HRMS) was recorded on a Waters, GCT Premier (EI-MS) with a TOF analyzer. Infrared spectra were recorded on a JASCO FT/IR-4100 spectrometer.

Dehydrated solvents such as CH_2Cl_2, *n*-hexane and Et_2O (water max 0.001%) were purchased from Wako Pure Chemical Industries, Ltd. and used under argon atmosphere. 1,4-Difluorobenzene was purchased from Tokyo Chemical Industry Co., Ltd., and were dried and distilled from 4Å molecule sieves under argon atmosphere, and were stored in glove box. Aluminum (III) chloride was purchased from Tokyo Chemical Industry Co., Ltd. (>98.0%, as light yellow powder). Organoaluminum reagents such as ethylaluminum dichloride (17% in hexane, ca. 1 mol/L), diethylaluminum chloride (ca. 15% in hexane, ca. 0.87 mol/L), triethylaluminum (15% in hexane, ca. 1.0 mol/L), was purchased from Tokyo Chemical Industry Co., Ltd., and used under argon atmosphere.

The preparation of spirobiindanes 2a-2f, 2h-2o and alkenyl chloride 3g, related to Figure 2

General procedure for the intramolecular Friedel-Craft reaction of *gem*-difluoroalkanes: In a flame-dried test tube (10 mL), to the heterogeneous solution of $AlCl_3$ (29.3 mg, 0.22 mmol, 2.2 equiv.) in dry CH_2Cl_2 (0.5 mL), *gem*-difluoroalkanes **1** (0.1 mmol) in dry CH_2Cl_2 (0.5 mL) was added dropwise by syringe, and the reaction mixture was stirred at room temperature for 2 hours under a positive pressure of argon with a balloon. Then, the resulting mixture was washed with water, extracted with CH_2Cl_2, dried over Na_2SO_4, filtered, and then concentrated *in vacuo*. The residue was purified by column chromatography on silica gel using *n*-hexane as the eluent to afford the desired spirobiindanes **2a-2f**, **2h-2o**. In addition, alkenyl chloride **3g**, 2,2'-(3-chloropent-2-ene-1,5-diyl)bis(methoxybenzene), was also prepared as one special example. In addition, the *gem*-difluoroalkanes **1** were prepared based on previous reports via fluorination of corresponding ketones by (diethylamino)sulfur trifluoride or 4-*tert*-butyl-2,6-dimethylphenylsulfur trifluoride (Fluolead).[1]

2,2',3,3'-Tetrahydro-1,1'-spirobi[indene] **2a**[1,2]

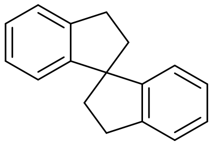

(3,3-Difluoropentane-1,5-diyl)dibenzene **1a** (26.0 mg, 0.1 mmol) in CH_2Cl_2 (0.5 mL) was slowly added to a solution of $AlCl_3$ (29.3 mg, 0.22 mmol, 2.2 equiv.) in dry CH_2Cl_2 (0.5 mL). And the resulting mixture was stirred at room temperature for 2 hours under argon atmosphere. The residue was purified by column chromatography on silica gel (*n*-hexane) to give **2a** (17.5 mg, 79%) as a colorless oil. ^{1}H NMR ($CDCl_3$, 300 MHz) δ 7.29–7.20 (m, 2H), 7.17–7.08 (m, 4H), 6.94–6.86 (m, 2H), 3.04–2.94 (m, 4H), 2.35-2.13 (m, 4H). MS (EI, *m*/*z*) 220 $[M]^+$

4,4’-Dimethyl-2,2',3,3'-tetrahydro-1,1'-spirobi[indene] **2b**[1]

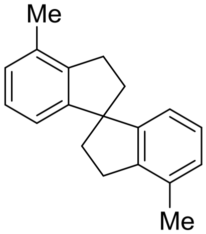

2,2'-(3,3-Difluoropentane-1,5-diyl)bis(methylbenzene) **1b** (28.8 mg, 0.1 mmol) in CH_2Cl_2 (0.5 mL) was slowly added to a solution of $AlCl_3$ (29.3 mg, 0.22 mmol, 2.2 equiv.) in dry CH_2Cl_2 (0.5 mL). And the resulting mixture was stirred at room temperature for 2 hours under argon atmosphere. The purification by column chromatography on silica gel (*n*-hexane) to give **2b** (20.1 mg, 81%) as a white solid. ^{1}H NMR (300 MHz, $CDCl_3$) δ 7.12–6.99 (m, 4H), 6.76 (d, *J* = 7.2 Hz, 2H), 2.97–2.89 (m, 4H), 2.36–2.23 (m, 8H), 2.22–2.04 (m, 2H). MS (EI, *m*/*z*) 248 $[M]^+$

5,5'-Dimethyl-2,2',3,3'-tetrahydro-1,1'-spirobi[indene] **2c**[1]

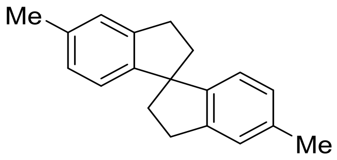

3,3'-(3,3-Difluoropentane-1,5-diyl)bis(methylbenzene) **1c** (28.8 mg, 0.1 mmol) in CH_2Cl_2 (0.5 mL) was slowly added to a solution of $AlCl_3$ (29.3 mg, 0.22 mmol, 2.2 equiv.) in dry CH_2Cl_2 (0.5 mL). And the resulting mixture was stirred at room temperature for 2 hours under argon atmosphere. The residue was purified by column chromatography on silica gel (*n*-hexane) to give **2c** (14.5 mg, 57%) as a colorless oil. ^1H NMR (300 MHz, $CDCl_3$) δ 7.19 (d, *J* = 7.6 Hz, 2H), 7.11–6.98 (m, 2H), 6.75 (s, 2H), 2.96-2.87 (m, 4H), 2.34–2.16 (m, 8H), 2.23–2.09 (m, 2H). MS (EI, *m*/*z*) 248 $[M]^+$

6,6'-Dibutyl-2,2',3,3'-tetrahydro-1,1'-spirobi[indene] **2d**[1]

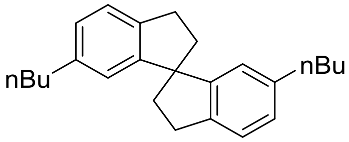

4,4'-(3,3-Difluoropentane-1,5-diyl)bis(butylbenzene) **1d** (30.0 mg, 0.08 mmol) in CH_2Cl_2 (0.5 mL) was slowly added to a solution of $AlCl_3$ (22.4 mg, 0.18 mmol, 2.2 equiv.) in dry CH_2Cl_2 (0.5 mL). And the resulting mixture was stirred at room temperature for 2 hours under argon atmosphere. The residue was purified by column chromatography on silica gel (*n*-hexane) to give **2d** (12.6 mg, 46%) as a colorless oil. ^1H NMR (300 MHz, $CDCl_3$) δ 7.20 (d, *J* = 7.6 Hz, 2H), 7.06–6.97 (m, 2H), 6.76 (s, 2H), 2.99–2.85 (m, 4H), 2.56–2.46 (m, 4H), 2.38–2.25 (m, 2H), 2.22–2.11 (m, 2H), 1.53–1.44 (m, 4H), 1.30 (dq, *J* = 14.5, 7.2 Hz, 4H), 0.90 (t, *J* = 7.3 Hz, 6H). MS (EI, *m*/*z*) 332 $[M]^+$

6,6'-Diphenyl-2,2',3,3'-tetrahydro-1,1'-spirobi[indene] **2e**

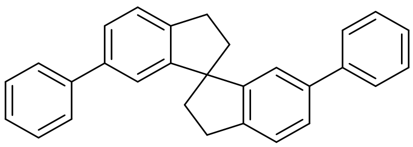

4,4''-(3,3-Difluoropentane-1,5-diyl)di-1,1'-biphenyl **1e** (41.2 mg, 0.1 mmol) in CH_2Cl_2 (0.5 mL) was slowly added to a solution of $AlCl_3$ (29.3 mg, 0.22 mmol, 2.2 equiv.) in dry CH_2Cl_2 (0.5 mL). And the resulting mixture was stirred at room temperature for 2 hours under argon atmosphere. The residue was purified by column chromatography on silica gel (*n*-hexane) to give **2e** (15.3 mg, 40%) as a sticky colorless oil. ^1H NMR (300 MHz, $CDCl_3$) δ 7.55–7.43 (m, 6H), 7.41–7.32 (m, 6H), 7.31–7.18 (m, 4H), 3.19–3.00 (m, 4H), 2.46–2.20 (m, 4H). ^{13}C NMR (126 MHz, $CDCl_3$) δ 151.0, 143.1, 141.4, 140.1, 128.5, 127.1, 126.8, 125.9, 124.6, 122.1, 60.8, 40.9, 30.6. IR (KBr):

3027, 2929, 2844, 1654, 1571, 1479, 906, 825, 754 cm^{-1}. HRMS (EI) calcd. for $C_{29}H_{24}^+$ [M^+]: 372.1878 found 372.1888

4,4',5,5'-Tetramethyl-2,2',3,3'-tetrahydro-1,1'-spirobi[indene] **2f**[1]

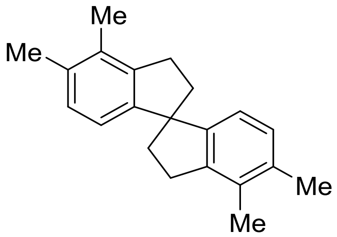

3,3'-(3,3-Difluoropentane-1,5-diyl)bis(1,2-dimethylbenzene) **1f** (31.6 mg, 0.1 mmol) in CH_2Cl_2 (0.5 mL) was slowly added to a solution of $AlCl_3$ (29.3 mg, 0.22 mmol, 2.2 equiv.) in dry CH_2Cl_2 (0.5 mL). And the resulting mixture was stirred at room temperature for 2 hours under argon atmosphere. The residue was purified by column chromatography on silica gel (*n*-hexane) to give **2f** (23.7 mg, 85%) as a white solid. ^1H NMR (300 MHz, $CDCl_3$) δ 6.96 (d, J = 7.6 Hz, 2H), 6.68 (d, J = 7.6 Hz, 2H), 2.94 (dd, J = 11.6, 6.0 Hz, 4H), 2.24–2.24 (m, 8H), 2.23 (s, 6H), 2.19–2.10 (m, 2H). MS (EI, *m/z*) 276 $[M]^+$

6,6'-Dibromo-2,2',3,3'-tetrahydro-1,1'-spirobi[indene] **2h**[1]

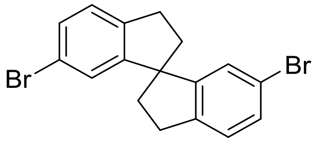

4,4'-(3,3-Difluoropentane-1,5-diyl)bis(bromobenzene) **1h** (41.9 mg, 0.1 mmol) in CH_2Cl_2 (0.5 mL) was slowly added to a solution of $AlCl_3$ (29.3 mg, 0.22 mmol, 2.2 equiv.) in dry CH_2Cl_2 (0.5 mL). And the resulting mixture was stirred at room temperature for 2 hours under argon atmosphere. The residue was purified by column chromatography on silica gel (*n*-hexane) to give **2h** (24.3 mg, 64%) as a white solid. ^1H NMR (300 MHz, $CDCl_3$) δ 7.33 (dd, J = 8.0, 1.8 Hz, 2H), 7.16 (d, J = 8.0 Hz, 2H), 7.02 (d, J = 1.5 Hz, 2H), 2.97 (dd, J = 8.2, 6.0 Hz, 4H), 2.33–2.21 (m, 2H), 2.24–2.16 (m, 2H). MS (EI, *m/z*) 376, 378, 380 $[M]^+$

6,6'-Dichloro-2,2',3,3'-tetrahydro-1,1'-spirobi[indene] **2i**[1]

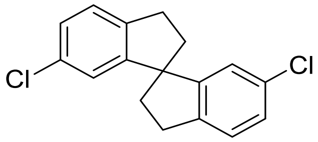

4,4'-(3,3-Difluoropentane-1,5-diyl)bis(chlorobenzene) **1i** (32.9 mg, 0.1 mmol) in CH_2Cl_2 (0.5 mL) was slowly added to a solution of $AlCl_3$ (29.3 mg, 0.22 mmol, 2.2 equiv.) in dry CH_2Cl_2 (0.5 mL). And the resulting mixture was stirred at room temperature for 2 hours under argon atmosphere.

The residue was purified by column chromatography on silica gel (*n*-hexane) to give **2i** (18.3 mg, 63%) as a white solid ^{1}H NMR (300 MHz, $CDCl_3$) δ 7.26–7.13 (m, 4H), 6.88 (d, *J* = 1.3 Hz, 2H), 2.96 (dd, *J* = 8.4, 5.9 Hz, 4H), 2.37–2.14 (m, 4H). MS (EI, *m*/*z*) 288 $[M]^+$

6,6'-Difluoro-2,2',3,3'-tetrahydro-1,1'-spirobi[indene] **2j**

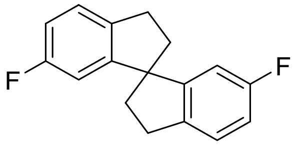

4,4'-(3,3-Difluoropentane-1,5-diyl)bis(fluorobenzene) **1j** (29.6 mg, 0.1 mmol) in CH_2Cl_2 (0.5 mL) was slowly added to a solution of $AlCl_3$ (29.3 mg, 0.22 mmol, 2.2 equiv.) in dry CH_2Cl_2 (0.5 mL). And the resulting mixture was stirred at room temperature for 2 hours under argon atmosphere. The residue was purified by column chromatography on silica gel (*n*-hexane) to give **2j** (11.0 mg, 43%) as light yellow semi-solid. ^{1}H NMR (300 MHz, $CDCl_3$) δ 7.25–7.12 (m, 2H), 6.88 (td, *J* = 9.0, 2.3 Hz, 2H), 6.60 (dd, *J* = 8.9, 2.0 Hz, 2H), 3.04–2.88 (m, 4H), 2.41–2.27 (m, 2H), 2.25–2.12 (m, 2H). ^{13}C NMR (126 MHz, $CDCl_3$) δ 162.3 (d, *J* = 243.3 Hz), 151.9 (d, *J* = 7.2 Hz), 138.8 (d, *J* = 2.4 Hz), 125.3 (d, *J* = 8.6 Hz), 113.8 (d, *J* = 22.5 Hz), 110.2 (d, *J* = 22.1 Hz), 61.0, 40.8, 30.1. IR (KBr): 2940, 2857, 1614, 1479, 1265, 1172, 869, 809 cm^{-1}. HRMS (EI) calcd. for $C_{17}H_{14}F_2$ $[M^+]$: 256.1064 found 256.1062

4,4'-Difluoro-2,2',3,3'-tetrahydro-1,1'-spirobi[indene] **2k**[1]

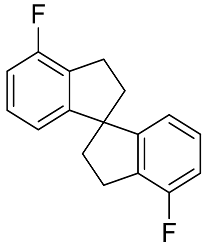

2,2'-(3,3-Difluoropentane-1,5-diyl)bis(fluorobenzene) **1k** (29.6 mg, 0.1 mmol) in CH_2Cl_2 (0.5 mL) was slowly added to a solution of $AlCl_3$ (29.3 mg, 0.22 mmol, 2.2 equiv.) in dry CH_2Cl_2 (0.5 mL). And the resulting mixture was stirred at room temperature for 2 hours under argon atmosphere. The residue was purified by column chromatography on silica gel (*n*-hexane) to give **2k** (10.9 mg, 42%) as a white solid. ^{1}H NMR (300 MHz, $CDCl_3$) δ 7.16–7.08 (m, 2H), 6.96–6.83 (m, 2H), 6.71 (d, *J* = 7.5 Hz, 2H), 3.13–2.95 (m, 4H), 2.35 (ddd, *J* = 11.6, 7.5, 2.0 Hz, 2H), 2.26–2.14 (m, 2H). MS (EI, *m*/*z*) 256 $[M]^+$

4,4'-Dibromo-2,2',3,3'-tetrahydro-1,1'-spirobi[indene] **2l**[1]

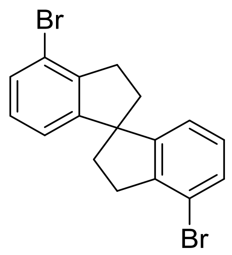

2,2'-(3,3-Difluoropentane-1,5-diyl)bis(bromobenzene) **1l** (41.5 mg, 0.1 mmol) in CH_2Cl_2 (0.5 mL) was slowly added to a solution of $AlCl_3$ (29.3 mg, 0.22 mmol, 2.2 equiv.) in dry CH_2Cl_2 (0.5 mL). And the resulting mixture was stirred at room temperature for 2 hours under argon atmosphere. The residue was purified by column chromatography on silica gel (*n*-hexane) to give **2l** (18.8 mg, 49%) as a white solid. ^{1}H NMR (300 MHz, $CDCl_3$) δ 7.37–7.30 (m, 2H), 7.03 (t, *J* = 7.7 Hz, 2H), 6.86 (d, *J* = 7.5 Hz, 2H), 3.09–2.96 (m, 4H), 2.37–2.25 (m, 2H), 2.24–2.14 (m, 2H). MS (EI, *m*/*z*) 376, 378, 380 $[M]^+$

2,2',3,3'-Tetrahydro-1,1'-spirobi[cyclopenta[*b*]naphthalene] **2m**[1]

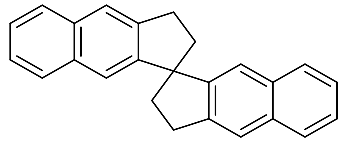

2,2'-(3,3-Difluoropentane-1,5-diyl)dinaphthalene **1m** (36.0 mg, 0.1 mmol) in CH_2Cl_2 (0.5 mL) was slowly added to a solution of $AlCl_3$ (29.3 mg, 0.22 mmol, 2.2 equiv.) in dry CH_2Cl_2 (0.5 mL). And the resulting mixture was stirred at room temperature for 2 hours under argon atmosphere. The residue was purified by column chromatography on silica gel (*n*-hexane) to give **2m** (22.2 mg, 66%) as a white solid. ^{1}H NMR (300 MHz, $CDCl_3$) δ 7.84–7.75 (m, 4H), 7.67 (d, *J* = 7.8 Hz, 2H), 7.39–7.27 (m, 6H), 3.25–3.12 (m, 4H), 2.49–2.31 (m, 4H). MS (EI, *m*/*z*) 320 $[M]^+$

4-Bromo-4'-methyl-2,2',3,3'-tetrahydro-1,1'-spirobi[indene] **2n**[1]

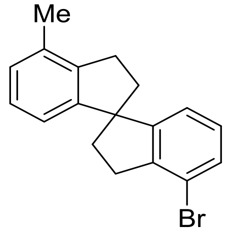

1-Bromo-2-(3,3-difluoro-5-(*o*-tolyl)pentyl)benzene **1n** (44.4 mg, 0.12 mmol) in CH_2Cl_2 (0.5 mL) was slowly added to a solution of $AlCl_3$ (36.3 mg, 0.27 mmol, 2.2 equiv.) in dry CH_2Cl_2 (0.5 mL). And the resulting mixture was stirred at room temperature for 2 hours under argon atmosphere. The purification by column chromatography on silica gel (*n*-hexane) to give **2n** (17.2 mg, 54%) as a white solid. ^{1}H NMR (300 MHz, $CDCl_3$) δ 7.33 (d, *J* = 7.8 Hz, 1H), 7.13–7.03 (m, 3H), 6.85 (d, *J* = 7.4 Hz, 1H), 6.76 (d, *J* = 7.1 Hz, 1H), 3.08–2.98 (m, 2H), 2.98–2.90 (m, 2H), 2.32–2.21 (m,

5H), 2.19–2.09 (m, 2H). MS (EI, *m/z*) 312, 314 $[M]^+$

3,3',4,4'-Tetrahydro-2*H*,2'*H*-1,1'-spirobi[naphthalene] **2o**[1]

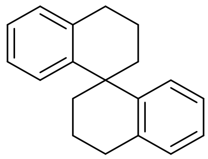

(4,4-Difluoroheptane-1,7-diyl)dibenzene **1o** (28.8 mg, 0.1 mmol) in CH_2Cl_2 (0.5 mL) was slowly added to a solution of $AlCl_3$ (29.3 mg, 0.22 mmol, 2.2 equiv.) in dry CH_2Cl_2 (0.5 mL). And the resulting mixture was stirred at room temperature for 2 hours under argon atmosphere. The residue was purified by column chromatography on silica gel (*n*-hexane) to give **2o** (19.6 mg, 79%) as a white solid. ^1H NMR (300 MHz, $CDCl_3$) δ 7.13–6.99 (m, 6H), 6.77 (d, *J* = 7.5 Hz, 2H), 2.99–2.87 (m, 4H), 2.18–2.10 (m, 2H), 1.94–1.83(m, 6H). MS (EI, *m/z*) 248 $[M]^+$

2,2'-(3-Chloropent-2-ene-1,5-diyl)bis(methoxybenzene) **3g**[1]

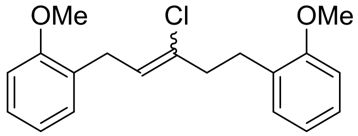

2,2'-(3,3-Difluoropentane-1,5-diyl)bis(methoxybenzene) **1g** (32.0 mg, 0.1 mmol) in CH_2Cl_2 (0.5 mL) was slowly added to a solution of $AlCl_3$ (29.3 mg, 0.22 mmol, 2.2 equiv.) in dry CH_2Cl_2 (0.5 mL). And the resulting mixture was stirred at room temperature for 2 hours under argon atmosphere. The residue was purified by column chromatography on silica gel (*n*-hexane) to give **3g** (12.0 mg, 38%) as a colorless oil. The ratio for *Z*/*E* isomers (9.4:1.0) was determined by ^1H NMR. (*Z*)-**3g**: ^1H NMR (300 MHz, $CDCl_3$) δ 7.22–7.07 (m, 3H), 6.98 (d, *J* = 7.0 Hz, 1H), 6.93–6.76 (m, 4H), 5.58 (t, *J* = 7.0 Hz, 1H), 3.81 (s, 6H), 3.48 (d, *J* = 6.9 Hz, 2H), 2.89 (t, *J* = 7.3 Hz, 2H), 2.62 (t, *J* = 7.3 Hz, 2H). ^{13}C NMR (126 MHz, $CDCl_3$) δ 157.4, 157.1, 135.0, 130.1, 129.4, 129.1, 128.0, 127.28, 127.25, 123.8, 120.4, 120.3, 110.14, 110.08, 55.2, 55.1, 39.5, 29.0, 28.7. MS (EI, *m/z*) 316 $[M]^+$. IR (KBr): 2996, 2948, 1587, 1436, 1459, 1494, 1307, 1108, 1029, 757 cm^{-1}. HRMS (EI) calcd. for $C_{19}H_{21}ClO_2$ $[M^+]$: 316.1230 found 316.1238

The preparation of alkenyl chloride 3, related to Figure 3

General procedure for the synthesis of alkenyl chlorides **3** from *gem*-difluoroalkanes **1**: In a flame-dried test tube (10 mL), diethylaluminum chloride (255 μL, ca. 0.22 mmol, 2.2 equiv, ca. 15% in hexane, ca. 0.87 mol/L) was added slowly to the solution of *gem*-difluoroalkanes **1** (0.1 mmol) in dry CH_2Cl_2 (0.1 M, 1.0 mL), and the reaction mixture was stirred at room temperature for 4 hours under a positive pressure of argon with a balloon. Then, the resulting mixture was washed with water, extracted with CH_2Cl_2, dried over Na_2SO_4, filtered, and then concentrated *in vacuo*. The residue was purified by column chromatography on silica gel using *n*-hexane as the eluent to afford

the desired alkenyl chloride **3**. The ratio for *Z*/*E* isomers was determined by ^{1}H NMR based on previous literature.[3,5]

(3-Chloropent-2-ene-1,5-diyl)dibenzene **3a**[4]

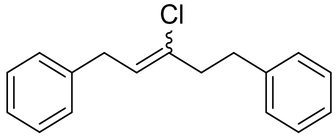

Diethylaluminum chloride (255 μL, 0.22 mmol, 2.2 equiv.) was added slowly to the solution of 3,3-difluoropentane-1,5-diyl)dibenzene **1a** (26.0 mg, 0.1 mmol) in dry CH_2Cl_2 (1.0 mL), and the resulting mixture was stirred at room temperature for 4 hours under argon atmosphere. The residue was purified by column chromatography on silica gel (*n*-hexane) to give **3a** (24.1 mg, 92%) as a colorless oil. The ratio for *Z*/*E* isomers (12:1) was determined by ^{1}H NMR. (*Z*)-**3a**: ^{1}H NMR (300 MHz, $CDCl_3$) δ 7.37–7.15 (m, 8H), 7.06 (d, *J* = 7.1 Hz, 2H), 5.55 (t, *J* = 7.1 Hz, 1H), 3.49 (d, *J* = 7.0 Hz, 2H), 2.90 (t, *J* = 7.4 Hz, 2H), 2.65 (t, *J* = 7.4 Hz, 2H). ^{13}C NMR (126 MHz, $CDCl_3$) δ 140.6, 139.2, 134.5, 128.5, 128.41, 128.35, 128.3, 126.1, 126.0, 125.0, 41.4, 34.7, 33.7. MS (EI, *m*/*z*) 256 $[M]^+$.

2,2'-(3-Chloropent-2-ene-1,5-diyl)bis(methylbenzene) **3b**

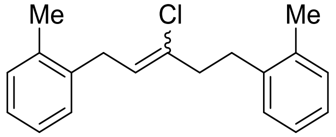

Diethylaluminum chloride (255 μL, 0.22 mmol, 2.2 equiv.) was added slowly to the solution of 2,2'-(3,3-difluoropentane-1,5-diyl)bis(methylbenzene) **1b** (28.8 mg, 0.1 mmol) in dry CH_2Cl_2 (1.0 mL), and the resulting mixture was stirred at room temperature for 4 hours under argon atmosphere. The residue was purified by column chromatography on silica gel (*n*-hexane) to give **3b** (27.4 mg, 96%) as a colorless oil. The ratio for *Z*/*E* isomers (13.5:1.0) was determined by ^{1}H NMR. (*Z*)-**3b**: ^{1}H NMR (300 MHz, $CDCl_3$) δ 7.21–7.10 (m, 7H), 7.10–7.02 (m, 1H), 5.54 (t, *J* = 6.9 Hz, 1H), 3.51 (d, *J* = 6.9 Hz, 2H), 3.01–2.84 (m, 2H), 2.66–2.56 (m, 2H), 2.34 (s, 3H), 2.29 (s, 3H). ^{13}C NMR (126 MHz, $CDCl_3$) δ 138.9, 137.9, 136.3, 135.9, 134.7, 130.2, 130.1, 129.1, 128.7, 126.4, 126.2, 126.1, 126.0, 124.3, 40.2, 32.8, 31.4, 19.5, 19.3. IR (KBr): 3019, 2940, 2873, 1666, 1598, 1486, 1468, 1049, 750 cm^{-1}. MS (EI, *m*/*z*) 284 $[M]^+$; HRMS (EI) calcd. for $C_{19}H_{21}Cl$ $[M^+]$: 284.1332 found 284.1338.

4,4'-(3-Chloropent-2-ene-1,5-diyl)bis(ethylbenzene) **3p**

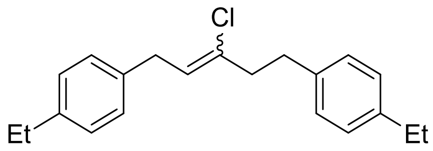

Diethylaluminum chloride (255 μL, 0.22 mmol, 2.2 equiv.) was added slowly to the solution of 4,4'-(3,3-difluoropentane-1,5-diyl)bis(ethylbenzene) **1p** (31.4 mg, 0.1 mmol) in dry CH_2Cl_2 (1.0 mL), and the resulting mixture was stirred at room temperature for 4 hours under argon atmosphere. The residue was purified by column chromatography on silica gel (*n*-hexane) to give **3p** (27.8 mg, 89%) as a colorless oil. The ratio for *Z*/*E* isomers (12.4:1.0) was determined by ^{1}H NMR. (*Z*)-**3p**: ^{1}H NMR (300 MHz, $CDCl_3$) δ 7.19–7.07 (m, 6H), 7.08–6.98 (m, 2H), 5.58 (t, *J* = 7.1 Hz, 1H), 3.48 (d, *J* = 7.1 Hz, 2H), 2.95–2.81 (m, 2H), 2.70–2.56 (m, 6H), 1.25 (t, *J* = 7.6 Hz, 3H), 1.24 (t, *J* = 7.6 Hz, 3H). ^{13}C NMR (126 MHz, $CDCl_3$) δ 142.0, 141.8, 137.8, 136.7, 134.4, 128.4, 128.2, 127.9, 127.8, 125.0, 41.5, 34.3, 33.3, 28.4, 15.7, 15.6. IR (KBr): 3012, 2964, 2929, 2857, 1658, 1511, 1448, 1056 cm^{-1}. MS (EI, *m*/*z*) 312 $[M]^+$; HRMS (EI) calcd. for $C_{21}H_{25}Cl$ $[M^+]$: 312.1645 found 312.1647.

4,4'-(3-Chloropent-2-ene-1,5-diyl)bis(butylbenzene) **3d**

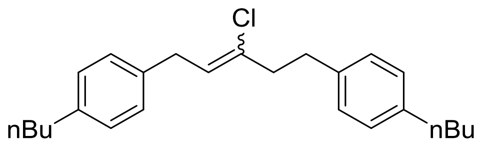

Diethylaluminum chloride (255 μL, 0.22 mmol, 2.2 equiv.) was added slowly to the solution of 4,4'-(3,3-difluoropentane-1,5-diyl)bis(butylbenzene) **1d** (37.2 mg, 0.1 mmol) in dry CH_2Cl_2 (1.0 mL), and the resulting mixture was stirred at room temperature for 4 hours under argon atmosphere. The residue was purified by column chromatography on silica gel (*n*-hexane) to give **3d** (36.3 mg, 95%) as a colorless oil. The ratio for *Z*/*E* isomers (13.6:1.0) was determined by ^{1}H NMR. (*Z*)-**3d**: ^{1}H NMR (300 MHz, $CDCl_3$) δ 7.16–7.03 (m, 6H), 7.04–6.94 (m, 2H), 5.55 (td, *J* = 7.1, 0.7 Hz, 1H), 3.46 (d, *J* = 7.1 Hz, 2H), 2.91–2.78 (m, 2H), 2.69–2.46 (m, 6H), 1.67–1.47 (m, 4H), 1.42–1.20 (m, 4H), 0.92 (t, *J* = 7.3 Hz, 6H). ^{13}C NMR (126 MHz, $CDCl_3$) δ 140.6, 140.5, 137.8, 136.7, 134.4, 128.43, 128.37, 128.3, 128.1, 125.0, 41.4, 35.2, 35.2, 34.3, 33.7, 33.3, 22.41, 22.38, 13.98, 13.96. IR (KBr): 3019, 2952, 2925, 2869, 1650, 1452, 1511, 1076, 825 cm^{-1}. MS (EI, *m*/*z*) 368 $[M]^+$; HRMS (EI) calcd. for $C_{25}H_{33}Cl$ $[M^+]$: 368.2271 found 368.2274.

4,4'-(3-Chloropent-2-ene-1,5-diyl)bis(bromobenzene) **3h**

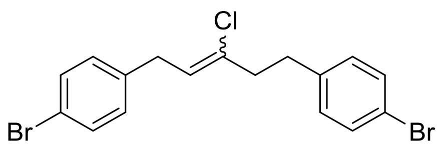

Diethylaluminum chloride (255 μL, 0.22 mmol, 2.2 equiv.) was added slowly to the solution of

4,4'-(3,3-difluoropentane-1,5-diyl)bis(bromobenzene) **1h** (41.8 mg, 0.1 mmol) in dry CH_2Cl_2 (1.0 mL), and the resulting mixture was stirred at room temperature for 4 hours under argon atmosphere. The residue was purified by column chromatography on silica gel (*n*-hexane) to give **3h** (34.6 mg, 82%) as a colorless oil. The ratio for *Z*/*E* isomers (29.4:1.0) was determined by ^{1}H NMR. (*Z*)-**3h**: ^{1}H NMR (300 MHz, $CDCl_3$) δ 7.37 (d, *J* = 8.0 Hz, 4H), 7.03 (d, *J* = 8.1 Hz, 2H), 6.86 (d, *J* = 8.1 Hz, 2H), 5.44 (t, *J* = 7.1 Hz, 1H), 3.40 (d, *J* = 7.0 Hz, 2H), 2.85 (t, *J* = 7.1 Hz, 2H), 2.62 (t, *J* = 7.1 Hz, 2H). ^{13}C NMR (126 MHz, $CDCl_3$) δ 139.3, 138.3, 134.5, 131.5, 131.3, 130.4, 130.0, 124.8, 119.97, 119.95, 41.0, 34.1, 32.9. IR (KBr): 3023, 2918, 1695, 1606, 1590, 1482, 1403, 1093, 809 cm^{-1}. MS (EI, *m*/*z*) 411 [M]$^+$; HRMS (EI) calcd. for $C_{17}H_{15}Br_2Cl$ [M$^+$]: 411.9229 found 411.9243.

4,4'-(3-Chloropent-2-ene-1,5-diyl)bis(chlorobenzene) **3i**

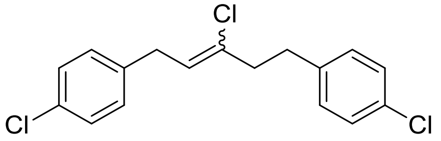

Diethylaluminum chloride (255 μL, 0.22 mmol, 2.2 equiv.) was added slowly to the solution of 4,4'-(3,3-difluoropentane-1,5-diyl)bis(chlorobenzene) **1i** (32.9 mg, 0.1 mmol) in dry CH_2Cl_2 (1.0 mL), and the resulting mixture was stirred at room temperature for 4 hours under argon atmosphere. The residue was purified by column chromatography on silica gel (*n*-hexane) to give **3i** (25.8mg, 79%) as a colorless oil. The ratio for *Z*/*E* isomers (20.1:1.0) was determined by ^{1}H NMR. (*Z*)-**3i**: ^{1}H NMR (300 MHz, $CDCl_3$) δ 7.27–7.16 (m, 4H), 7.13–7.03 (m, 2H), 6.97–6.88 (m, 2H), 5.45 (t, *J* = 7.2 Hz, 1H), 3.42 (d, *J* = 7.2 Hz, 2H), 2.86 (t, *J* = 7.2 Hz, 2H), 2.62 (t, *J* = 7.3 Hz, 2H). ^{13}C NMR (126 MHz, $CDCl_3$) δ 138.8, 137.7, 134.4, 131.88, 131.85, 129.9, 129.5, 128.5, 128.4, 124.9, 41.1, 33.9, 32.8. IR (KBr): 3027, 2929, 1695, 1602, 1494, 1400, 1101, 1004, 802 cm^{-1}. MS (EI, *m*/*z*) 324 [M]$^+$; HRMS (EI) calcd. for $C_{17}H_{15}Cl_3$ [M$^+$]: 324.0239 found 324.0245.

2,2'-(3-Chloropent-2-ene-1,5-diyl)bis(fluorobenzene) **3k**

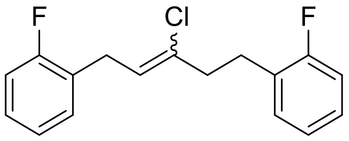

Diethylaluminum chloride (255 μL, 0.22 mmol, 2.2 equiv.) was added slowly to the solution of 2,2'-(3,3-difluoropentane-1,5-diyl)bis(fluorobenzene) **1k** (29.6 mg, 0.1 mmol) in dry CH_2Cl_2 (1.0 mL), and the resulting mixture was stirred at room temperature for 4 hours under argon atmosphere. The residue was purified by column chromatography on silica gel (*n*-hexane) to give **3k** (24.3 mg, 83 %) as a colorless oil. The ratio for *Z*/*E* isomers (13.2:1.0) was determined by ^{1}H NMR. (*Z*)-**3k**: ^{1}H NMR (300 MHz, $CDCl_3$) δ 7.22–7.11 (m, 3H), 7.10–6.89 (m, 5H), 5.54 (t, *J* = 7.1 Hz, 1H), 3.50 (d, *J* = 7.1 Hz, 2H), 2.93 (t, *J* = 7.3 Hz, 2H), 2.65 (t, *J* = 7.4 Hz, 2H). ^{13}C NMR (126 MHz, $CDCl_3$) δ 161.1 (d, *J* = 245.0 Hz), 160.8 (d, *J* = 245.4 Hz), 134.9, 131.0 (d, *J* = 5.1 Hz), 130.3 (d,

J = 4.6 Hz), 127.9 (d, J = 8.0 Hz), 127.8 (d, J = 7.8 Hz), 127.3 (d, J = 15.8 Hz), 126.3 (d, J = 15.9 Hz), 124.0 (d, J = 3.6 Hz), 123.9 (d, J = 3.5 Hz), 123.6, 115.2 (d, J = 10.4 Hz), 115.1 (d, J = 10.3 Hz), 39.8 (d, J = 1.2 Hz), 27.9 (d, J = 3.2 Hz), 27.3 (d, J = 2.1 Hz). IR (KBr): 3045, 2933, 1654, 1590, 1490, 1459, 1128, 1191, 1112, 746 cm^{-1}. MS (EI, *m*/*z*) 292 $[M]^+$; HRMS (EI) calcd. for $C_{17}H_{15}ClF_2$ $[M^+]$: 292.0830 found 292.0830.

4,4'-(3-Chloropent-2-ene-1,5-diyl)bis(1,3-dimethylbenzene) **3q**

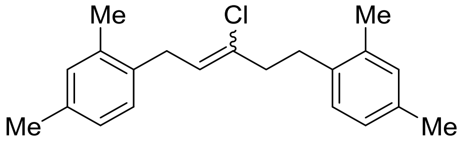

Diethylaluminum chloride (128 μL, 0.11 mmol, 2.2 equiv.) was added slowly to the solution of 4,4'-(3,3-difluoropentane-1,5-diyl)bis(1,3-dimethylbenzene) **1q** (15.7 mg, 0.05 mmol) in dry CH_2Cl_2 (0.5 mL), and the resulting mixture was stirred at room temperature for 4 hours under argon atmosphere. The residue was purified by column chromatography on silica gel (*n*-hexane) to give **3q** (13.2 mg, 82 %) as a colorless oil. The ratio for *Z*/*E* isomers (14.2:1.0) was determined by ^{1}H NMR. (*Z*)-**3q**: ^{1}H NMR (300 MHz, $CDCl_3$) δ 7.11–6.96 (m, 3H), 6.95–6.83 (m, 3H), 5.49 (t, J = 6.8 Hz, 1H), 3.44 (d, J = 6.7 Hz, 2H), 2.89–2.79 (m, 2H), 2.60–2.50 (m, 2H), 2.28 (s, 6H), 2.27 (s, 3H), 2.23 (s, 3H). ^{13}C NMR (126 MHz, $CDCl_3$) δ 136.1, 135.9, 135.7, 135.6, 134.8, 134.5, 131.1, 131.0, 129.0, 128.7, 126.6, 124.4, 40.4, 32.4, 31.1, 20.9, 19.4, 19.2. IR (KBr): 3008, 2960, 2869, 1606, 1650, 1502, 1442, 1378, 1072, 1033, 809 cm^{-1}. MS (EI, *m*/*z*) 312 $[M]^+$; HRMS (EI) calcd. for $C_{21}H_{25}Cl$ $[M^+]$: 312.1645 found 312.1645

(2-Chloroprop-1-ene-1,3-diyl)dibenzene **3r**[3]

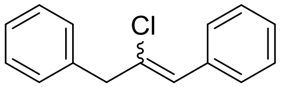

Diethylaluminum chloride (255 μL, 0.22 mmol, 2.2 equiv.) was added slowly to the solution of (2,2-difluoropropane-1,3-diyl)dibenzene **1r** (23.2 mg, 0.1 mmol) in dry CH_2Cl_2 (1.0 mL), and the resulting mixture was stirred at room temperature for 4 hours under argon atmosphere. The residue was purified by column chromatography on silica gel (*n*-hexane) to give (*Z*)-**3r** (15.1 mg, 66 %) and (*E*)-**3r** (3.0 mg, 13%), as the colorless oil. (*Z*)-**3r**: ^{1}H NMR (300 MHz, $CDCl_3$) δ 7.60 (d, J = 7.3 Hz, 2H), 7.47–7.10 (m, 8H), 6.54 (s, 1H), 3.79 (s, 2H). ^{13}C NMR (126 MHz, $CDCl_3$) δ 137.2, 134.8, 133.3, 129.1, 129.0, 128.5, 128.1, 127.6, 126.9, 125.8, 47.2. MS (EI, *m*/*z*) 228 $[M]^+$. (*E*)-**3r**: ^{1}H NMR (300 MHz, $CDCl_3$) δ 7.42–7.31 (m, 4H), 7.29-7.13 (m, 6H), 6.95 (s, 1H), 3.92 (s, 2H). ^{13}C NMR (126 MHz, $CDCl_3$) δ 137.1, 135.5, 135.1, 130.0, 128.6, 128.4, 128.1, 127.5, 126.7, 40.5. MS (EI, *m*/*z*) 228 $[M]^+$.

(3-Chlorobut-2-en-1-yl)benzene **3s**[5]

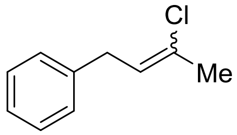

Diethylaluminum chloride (255 μL, 0.22 mmol, 2.2 equiv.) was added slowly to the solution of (3,3-difluorobutyl)benzene **1s** (17.0 mg, 0.1 mmol) in dry CH_2Cl_2 (1.0 mL), and the resulting mixture was stirred at room temperature for 4 hours under argon atmosphere. The residue was purified by column chromatography on silica gel (*n*-hexane) to give **3s** (11.9 mg, 71%), as a colorless oil. The ratio for *Z*/*E* isomers (8.1:1.0) was determined by ^1H NMR, along with 10% terminal alkene ((3-chlorobut-3-en-1-yl)benzene) as side product. (*Z*)-**3s**: ^1H NMR (300 MHz, $CDCl_3$) δ 7.34–7.25 (m, 2H), 7.24–7.12 (m, 3H), 5.64 (td, *J* = 7.2, 1.1 Hz, 1H), 3.51 (d, *J* = 7.1 Hz, 2H), 2.13 (d, *J* = 1.0 Hz, 3H). ^{13}C NMR (126 MHz, $CDCl_3$) δ 139.7, 130.8, 128.4, 128.3, 126.1, 124.5, 35.0, 26.1. MS (EI, *m*/*z*) 166 $[M]^+$.

4-Chloro-1,2,3,6-tetrahydro-1,1'-biphenyl **3t**[3]

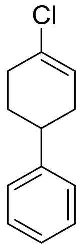

Diethylaluminum chloride (255 μL, 0.22 mmol, 2.2 equiv.) was added slowly to the solution of(4,4-difluorocyclohexyl)benzene **1t** (19.6 mg, 0.1 mmol) in dry CH_2Cl_2 (1.0 mL), and the resulting mixture was stirred at room temperature for 4 hours under argon atmosphere. The residue was purified by column chromatography on silica gel (*n*-hexane) to give **3t** (14.4 mg, 75%) as a colorless oil. ^1H NMR (300 MHz, $CDCl_3$) δ 7.37–7.27 (m, 2H), 7.26–7.17 (m, 3H), 6.00–5.75 (m, 1H), 2.88–2.73 (m, 1H), 2.64–2.44 (m, 2H), 2.44–2.32 (m, 1H), 2.31–2.15 (m, 1H), 2.07–1.80 (m, 2H). ^{13}C NMR (126 MHz, $CDCl_3$) δ 145.5, 131.7, 128.4, 126.7, 126.3, 124.0, 38.9, 33.9, 33.2, 30.6. MS (EI, *m*/*z*) 192 $[M]^+$.

1-Chloro-4-pentylcyclohex-1-ene **3u**

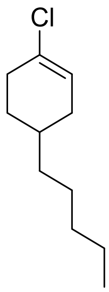

Diethylaluminum chloride (255 μL, 0.22 mmol, 2.2 equiv.) was added slowly to the solution of

1,1-difluoro-4-pentylcyclohexane **1u** (19.0 mg, 0.1 mmol) in dry CH_2Cl_2 (1.0 mL), and the resulting mixture was stirred at room temperature for 4 hours under argon atmosphere. The residue was purified by column chromatography on silica gel (*n*-hexane) to give **3u** (12.4 mg, 67%) as a colorless oil. ^{1}H NMR (300 MHz, $CDCl_3$) δ 5.75 (dd, *J* = 2.8, 1.9 Hz, 1H), 2.43–2.27 (m, 2H), 2.26–2.03 (m, 1H), 1.86–1.62 (m, 2H), 1.54–1.43 (m, 1H), 1.41–1.17 (m, 9H), 0.88 (t, *J* = 6.8 Hz, 3H). ^{13}C NMR (126 MHz, $CDCl_3$) δ 131.6, 124.0, 35.6, 32.7, 32.5, 32.3, 32.0, 29.8, 26.6, 22.6, 14.0. MS (EI, *m*/*z*) 186 $[M]^+$; HRMS (EI) calcd. for $C_{11}H_{19}Cl$ $[M^+]$: 186.1175 found 186.1167.

1-Chlorocyclododec-1-ene **3v**[6]

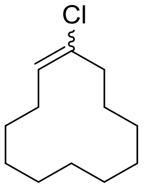

Diethylaluminum chloride (255 μL, 0.22 mmol, 2.2 equiv.) was added slowly to the solution of 1,1-difluorocyclododecane **1v** (20.4 mg, 0.1 mmol) in dry CH_2Cl_2 (1.0 mL), and the resulting mixture was stirred at room temperature for 4 hours under argon atmosphere. The residue was purified by column chromatography on silica gel (*n*-hexane) to give **3v** (13.5 mg, 67%). The ratio for *Z*/*E* isomers (1.7:1.0) was determined by ^{1}H NMR. (*Z*)-**3v**: ^{1}H NMR (300 MHz, $CDCl_3$) δ 5.57 (t, *J* = 8.2 Hz, 1H), 2.45–2.37 (m, 2H), 2.15–2.06 (m, , 2H), 1.73–1.52 (m, 3H), 1.51–1.42 (m, 2H), 1.41–1.19 (m, 11H). ^{13}C NMR (126 MHz, $CDCl_3$) δ 129.1, 128.1, 29.9, 26.7, 25.6, 24.7, 24.6, 24.3, 23.99, 23.90, 22.3, 22.0. (*E*)-**3v**: ^{1}H NMR (300 MHz, $CDCl_3$) δ 5.62 (t, *J* = 7.6 Hz, 1H), 2.43–2.34 (m, 2H), 2.27–2.19 (m, 2H), 1.70–1.53 (m, 3H), 1.49–1.41 (m, 2H), 1.40–1.33 (m, 11H). ^{13}C NMR (126 MHz, $CDCl_3$) δ 134.3, 133.8, 38.1, 28.2, 27.1, 26.0, 25.4, 25.1, 24.6, 23.94, 23.93, 23.6. MS (EI, *m*/*z*) 200 $[M]^+$.

The preparation of monofluoroalkene 4, related to Figure 4

General procedure for the synthesis of monofluoroalkene **4** from *gem*-difluoroalkanes **1**: In a flame-dried test tube (10 mL), triethylaluminum (150 μL, ca. 0.15 mmol, 1.5 equiv.,15% in hexane, ca. 1.0 mol/L) was added slowly to the solution of *gem*-difluoroalkanes **1** (0.1 mmol) in *n*-hexane (0.1 M, 1.0 mL), and the reaction mixture was stirred at room temperature for 7 hours under a positive pressure of nitrogen with a balloon. Then, the resulting mixture was washed with water, extracted with CH_2Cl_2, dried over Na_2SO_4, filtered, and then concentrated *in vacuo*. The residue was purified by column chromatography on silica gel to afford the desired monofluoroalkene **4**. The ratio for *Z*/*E* isomers was determined by ^{19}F NMR.

(3-Fluoropent-2-ene-1,5-diyl)dibenzene **4a**[1]

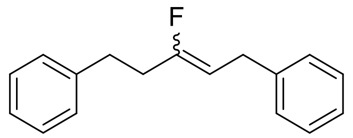

Triethylaluminium (150 μL, 15% in hexane, 0.15 mmol, 1.5 equiv.) was added dropwise to a solution of (3,3-difluoropentane-1,5-diyl)dibenzene **1a** (26.0 mg, 0.1 mmol) in dry *n*-hexane (1.0 mL). And the resulting mixture was stirred for 7 hours under nitrogen atmosphere at room temperature. The residue was purified by column chromatography on silica gel (*n*-hexane/CH_2Cl_2 = 19:1) to give **4a** (17.4 mg, 72%) as a colorless oil. The ratio for *Z*/*E* isomers (8.7:1.0) was determined by ^{19}F NMR. (*Z*)-**4a**: ^{1}H NMR (300 MHz, $CDCl_3$) δ 7.33–7.14 (m, 8H), 7.11 (d, *J* = 6.9 Hz, 2H), 4.67 (dt, *J* = 36.8, 7.6 Hz, 1H), 3.39 (d, *J* = 7.6 Hz, 2H), 2.88–2.80 (m, 2H), 2.57–2.43 (m, 2H); ^{19}F NMR (282 MHz, $CDCl_3$) δ −111.15–−111.43 (m, 1F). MS (EI, *m/z*) 240 $[M]^+$.

2,2'-(3-Fluoropent-2-ene-1,5-diyl)bis(methylbenzene) **4b**[1]

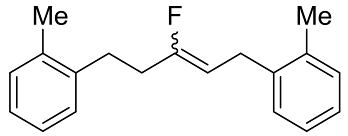

Triethylaluminium (150 μL, 15% in hexane, 0.15 mmol, 1.5 equiv.) was added dropwise to a solution of 2,2'-(3,3-difluoropentane-1,5-diyl)bis(methylbenzene) **1b** (29.0 mg, 0.1 mmol) in dry *n*-hexane (1.0 mL). And the resulting mixture was stirred for 9 hours under nitrogen atmosphere at room temperature. The residue was purified by column chromatography on silica gel (*n*-hexane/CH_2Cl_2 = 19:1) to give **4b** (18.6 mg, 69%) as a colorless oil. The ratio for *Z*/*E* isomers (9.0:1.0) was determined by ^{19}F NMR. (*Z*)-**4b**: ^{1}H NMR (300 MHz, $CDCl_3$) δ 7.18–7.04 (m, 8H), 4.62 (dt, *J* = 36.9, 7.4 Hz, 1H), 3.38 (d, *J* = 7.2 Hz, 2H), 2.90–2.76 (m, 2H), 2.52–2.36 (m, 2H), 2.30 (s, 3H), 2.28 (s, 3H); ^{19}F NMR (282 MHz, $CDCl_3$) δ −110.08–−110.38 (m, 1F). MS (EI, *m/z*) 268 $[M]^+$

4,4'-(3-Fluoropent-2-ene-1,5-diyl)bis(butylbenzene) **4d**[1]

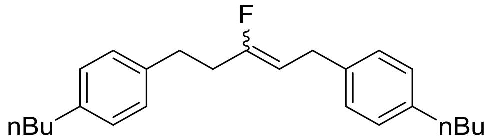

Triethylaluminium (150 μL, 15% in hexane, 0.15 mmol, 1.5 equiv.) was added dropwise to a solution of 4,4'-(3,3-difluoropentane-1,5-diyl)bis(butylbenzene) **1d** (37.2 mg, 0.1 mmol) in dry *n*-hexane (1.0 mL). And the resulting mixture was stirred for 7 hours under nitrogen atmosphere at room temperature. The residue was purified by column chromatography on silica gel (*n*-hexane/CH_2Cl_2 = 14:1) to give **4d** (27.0 mg, 77%) as a colorless oil. The ratio for *Z*/*E* isomers (7.7:1.0) was determined by ^{19}F NMR. (*Z*)-**4d**: ^{1}H NMR (300 MHz, $CDCl_3$) δ 7.14–6.99 (m, 8H),

4.66 (dt, J = 36.9, 7.5 Hz, 1H), 3.36 (d, J = 7.4 Hz, 2H), 2.80 (t, J = 7.8 Hz, 2H), 2.65–2.51 (m, 6H), 1.66–1.48 (m, 4H), 1.35 (dd, J = 14.1, 6.9 Hz, 4H), 0.92 (t, J = 7.1 Hz, 6H); ^{19}F NMR (282 MHz, $CDCl_3$) δ −111.19–−111.53 (m, 1F). MS (EI, *m/z*) 352 $[M]^+$

4,4'-(3-Fluoropent-2-ene-1,5-diyl)bis(chlorobenzene) **4i**[1]

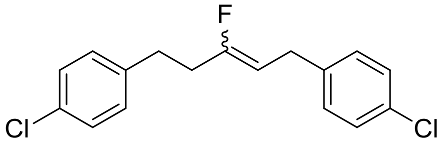

Triethylaluminium (150 μL, 15% in hexane, 0.15 mmol, 1.5 equiv.) was added dropwise to a solution of 4,4'-(3,3-difluoropentane-1,5-diyl)bis(chlorobenzene) **1i** (32.9 mg, 0.1 mmol) in dry CH_2Cl_2 (1.0 mL). And the resulting mixture was stirred for 2 hours under nitrogen atmosphere at room temperature. The purification by column chromatography on silica gel (*n*-hexane/CH_2Cl_2 = 14:1) to give **4i** (19.9 mg, 65%) as a colorless oil. The ratio for *Z*/*E* isomers (12:1) was determined by ^{19}F NMR. (*Z*)-**4i**: ^{1}H NMR (300 MHz, $CDCl_3$) δ 7.25–7.18 (m, 4H), 7.12–7.07 (m, 2H), 7.00–6.95 (m, 2H), 4.58 (dt, J = 36.5, 7.6 Hz, 1H), 3.33 (d, J = 7.5 Hz, 2H), 2.80 (t, J = 7.4 Hz, 2H), 2.55–2.40 (m, 2H); ^{19}F NMR (282 MHz, $CDCl_3$) δ −111.33 (dt, J = 35.9, 17.8 Hz, 1F). (*E*)-**4i**: ^{1}H NMR (300 MHz, $CDCl_3$) δ 7.25–7.15 (m, 4H), 7.15–7.07 (m, 2H), 6.84–6.76 (m, 2H), 5.17 (dt, J = 21.4, 8.0 Hz, 1H), 3.03 (d, J = 8.0 Hz, 2H), 2.83 (t, J = 7.1 Hz, 2H), 2.57 (dt, J = 22.1, 7.3 Hz, 2H); ^{19}F NMR (282 MHz, $CDCl_3$) δ −105.20–−105.59 (m, 1F). MS (EI, *m/z*) 308 $[M]^+$

4,4'-(3-Fluoropent-2-ene-1,5-diyl)bis(fluorobenzene) **4j**[1]

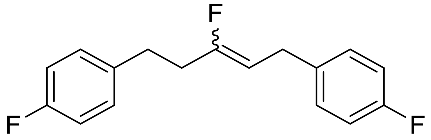

Triethylaluminium (150 μL, 15% in hexane, 0.15 mmol, 1.5 equiv.) was added dropwise to a solution of 4,4'-(3,3-difluoropentane-1,5-diyl)bis(fluorobenzene) **1j** (29.6 mg, 0.1 mmol) in dry *n*-hexane (1.0 mL). And the resulting mixture was stirred for 11 hours under nitrogen atmosphere at room temperature. The purification by column chromatography on silica gel (*n*-hexane/CH_2Cl_2 = 9:1) to give **4j** (15.4 mg, 55%) as a colorless oil. The ratio for *Z*/*E* isomers (10:1) was determined by ^{19}F NMR. (*Z*)-**4j**: ^{1}H NMR (300 MHz, $CDCl_3$) δ 7.16–7.09 (m, 2H), 7.05–6.90 (m, 6H), 4.60 (dt, J = 36.7, 7.6 Hz, 1H), 3.34 (d, J = 7.4 Hz, 2H), 2.81 (t, J = 7.5 Hz, 2H), 2.55–2.41 (m, 2H); ^{19}F NMR (282 MHz, $CDCl_3$) δ −111.41–−111.72 (m, 1F), −117.62–−117.87 (m, 1F), −117.94–−118.17 (m, 1F). (*E*)-**4j**: ^{1}H NMR (300 MHz, $CDCl_3$) δ 7.20–7.10 (m, 2H), 7.02–6.82 (m, 6H), 5.18 (dt, J = 21.2, 8.0 Hz, 1H), 3.04 (d, J = 8.0 Hz, 2H), 2.84 (t, J = 7.1 Hz, 2H), 2.58 (dt, J = 21.8, 6.9 Hz, 2H); ^{19}F NMR (282 MHz, $CDCl_3$) δ −105.62 (q, J = 22.0 Hz, 1F), −117.51–−117.66 (m, 1F), −117.66–−117.80 (m, 1F). MS (EI, *m/z*) 276 $[M]^+$

3,3'-(3-Fluoropent-2-ene-1,5-diyl)bis(1,2-dimethylbenzene) **4f**[1]

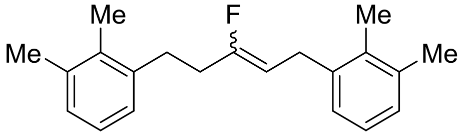

Triethylaluminium (150 *μ*L, 15% in hexane, 0.15 mmol, 1.5 equiv.) was added dropwise to a solution of 3,3'-(3,3-difluoropentane-1,5-diyl)bis(1,2-dimethylbenzene) **1f** (31.6 mg, 0.1 mmol) in dry *n*-hexane (1.0 mL). And the resulting mixture was stirred for 7 hours under nitrogen atmosphere at room temperature. The residue was purified by column chromatography on silica gel (*n*-hexane/CH_2Cl_2 = 9:1) to give **4f** (21.3 mg, 72%) as a colorless oil. The ratio for *Z*/*E* isomers (8.1:1.0) was determined by ^{19}F NMR. (*Z*)-**4f**: ^{1}H NMR (300 MHz, $CDCl_3$) δ 7.06–6.94 (m, 6H), 4.62 (dt, *J* = 37.2, 7.4 Hz, 1H), 3.41 (d, *J* = 7.3 Hz, 2H), 2.89–2.79 (m, 2H), 2.48–2.34 (m, 2H), 2.28 (s, 3H), 2.27 (s, 3H), 2.19 (s, 3H), 2.18 (s, 3H); ^{19}F NMR (282 MHz, $CDCl_3$) δ −109.97–−110.27 (m, 1F). MS (EI, *m/z*) 296 $[M]^+$.

4,4'-(3-Fluoropent-2-ene-1,5-diyl)bis(1,3-dimethylbenzene) **4q**[1]

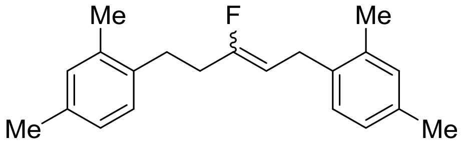

Triethylaluminium (150 *μ*L,15% in hexane, 0.15 mmol, 1.5 equiv.) was added dropwise to a solution of 4,4'-(3,3-difluoropentane-1,5-diyl)bis(1,3-dimethylbenzene) **1q** (31.6 mg, 0.1 mmol) in dry *n*-hexane (1.0 mL). And the resulting mixture was stirred for 7 hours under nitrogen atmosphere at room temperature. The purification by column chromatography on silica gel (*n*-hexane/CH_2Cl_2 = 9:1) to give **4q** (21.9 mg, 74%) as a colorless oil. The ratio for *Z*/*E* isomers (8.2:1.0) was determined by ^{19}F-NMR. (*Z*)-**4q**: ^{1}H NMR (300 MHz, $CDCl_3$) δ 7.04–6.90 (m, 6H), 4.60 (dt, *J* = 37.0, 7.4 Hz, 1H), 3.34 (d, *J* = 7.2 Hz, 2H), 2.81–2.73 (m, 2H), 2.47–2.33 (m, 2H), 2.28 (s, 6H), 2.26 (s, 3H), 2.25 (s, 3H); ^{19}F NMR (282 MHz, $CDCl_3$) δ −110.19–−110.48 (m, 1F). MS (EI, *m/z*) 296 $[M]^+$.

(2-Fluoroprop-1-ene-1,3-diyl)dibenzene **4r**[1,7]

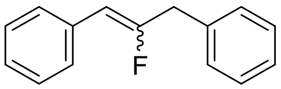

Triethylaluminium (150 *μ*L, 15% in hexane, 0.15 mmol, 1.5 equiv.) was added dropwise to a solution of (2,2-difluoropropane-1,3-diyl)dibenzene **1r** (23.2 mg, 0.1 mmol) in dry CH_2Cl_2 (1.0 mL). And the resulting mixture was stirred for 3 hours under nitrogen atmosphere at room temperature. The residue was purified by column chromatography on silica gel (*n*-hexane/CH_2Cl_2 = 9:1) to give **4r** (13.4 mg, 63%) as a colorless oil. The ratio for *Z*/*E* isomers (6.6:1.0) was

determined by ^{19}F-NMR. (*Z*)-**4r**: ^{1}H NMR (300 MHz, $CDCl_3$) δ 7.49–7.43 (m, 2H), 7.37–7.25 (m, 8H), 5.51 (d, *J* = 38.8 Hz, 1H), 3.64 (d, *J* = 17.0 Hz, 2H); ^{19}F NMR (282 MHz, $CDCl_3$) δ −100.66 (dt, *J* = 38.4, 17.0 Hz, 1F). MS (EI, *m/z*) 212 $[M]^+$

4-Fluoro-1,2,3,6-tetrahydro-1,1'-biphenyl **4t**[1,8]

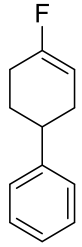

Triethylaluminium (150 *μ*L, 15% in hexane, 0.15 mmol, 1.5 equiv.) was added dropwise to a solution of (4,4-difluorocyclohexyl)benzene **1t** (19.6 mg, 0.1 mmol) in dry CH_2Cl_2 (1.0 mL). And the resulting mixture was stirred for 2 hours under nitrogen atmosphere at room temperature. The residue was purified by column chromatography on silica gel (*n*-hexane/CH_2Cl_2 = 19:1) to give **4t** (6.8 mg, 40%). ^{1}H NMR (300 MHz, $CDCl_3$) δ 7.36–7.28 (m, 2H), 7.26–7.21 (m, 3H), 5.32–5.23 (m, 1H), 2.86–2.73 (m, 1H), 2.44–2.16 (m, 4H), 2.08–1.83 (m, 2H); ^{19}F NMR (282 MHz, $CDCl_3$) δ −103.97–−104.10 (m, 1F). MS (EI, *m/z*) 176 $[M]^+$

1-Fluoro-4-pentylcyclohex-1-ene **4u**[1,8]

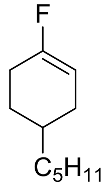

Triethylaluminium (300 *μ*L,15% in hexane, 0.30 mmol, 1.5 equiv.) was added dropwise to a solution of 1,1-difluoro-4-pentylcyclohexane **1u** (38.1 mg, 0.2 mmol) in dry *n*-hexane (2.0 mL). And the resulting mixture was stirred for 7 hours under nitrogen atmosphere at room temperature. The residue was purified by column chromatography on silica gel (pentane) to give **4u** (14.3 mg, 42%) as a colorless oil. ^{1}H NMR (300 MHz, $CDCl_3$) δ 5.19–5.11 (m, 1H), 2.25–2.09 (m, 3H), 1.86–1.80 (m, 1H), 1.73–1.62 (m, 1H), 1.52–1.46 (m, 1H), 1.43–1.20 (m, 9H), 0.88 (t, *J* = 6.8 Hz, 3H); ^{19}F NMR (282 MHz, $CDCl_3$)) δ −103.59–−103.77 (m, 1F); MS (EI, *m/z*) 170 $[M]^+$

1-Fluorocyclopentadec-1-ene **4w**[1]

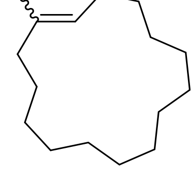

Triethylaluminium (150 μL, 15% in hexane, 0.15 mmol, 1.5 equiv.) was added dropwise to a solution of 1,1-difluorocyclopentadecane **1w** (24.6 mg, 0.1 mmol) in dry *n*-hexane (1.0 mL). And the resulting mixture was stirred for 7 hours under nitrogen atmosphere at room temperature. The purification by column chromatography on silica gel (*n*-hexane) to give **4w** (11.3 mg, 50%) as colorless oil. The ratio for *Z*/*E* isomers (5.1:1.0) was determined by ^{19}F NMR. (*Z*)-**4w**: ^{1}H NMR (300 MHz, $CDCl_3$) δ 4.45 (dt, *J* = 38.5, 7.2 Hz, 1H), 2.26–2.19 (m, 1H), 2.19–2.07 (m, 3H), 1.54–1.45 (m, 3H), 1.43–1.24 (m, 19H); ^{19}F NMR (282 MHz, $CDCl_3$) δ −111.92 (dt, *J* = 38.7, 19.3 Hz, 1F). MS (EI, *m/z*) 226 $[M]^+$

Supplementary Table 1. Optimization of $AlEt_3$ induced defluorinative elimination to afford monofluoroalkene **4**, related to **Table 1**

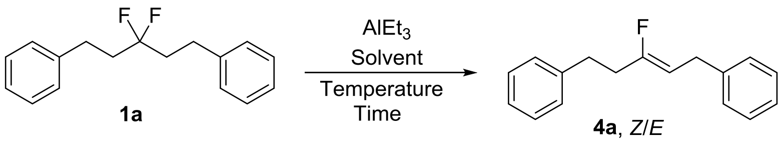

Entry	$AlEt_3$ (equiv.)	Solvent	Concentration (M)	T (°C)	*t* (h)	Yield[a]	*Z*/*E*[a]
1	2.2	CH_2Cl_2	0.1	RT	0.5	51	7.3:1.0
2	2.2	CH_2Cl_2	0.1	0	2	29	6.7:1.0
3	2.2	CH_2Cl_2	0.1	40	10 min	47	6.1:1.0
4	2.2	hexane	0.1	RT	5	68	9.1:1.0
5	2.2	hexane/CH_2Cl_2 (1:1)	0.1	RT	2	63	8.2:1.0
6	2.2	1,4-$C_6H_4F_2$	0.1	RT	2	62	10:1
7	2.2	$ClCH_2CH_2Cl$	0.1	RT	1	53	9.2:1.0
8	2.2	Et_2O	0.1	RT	8	NR	---
9	1.5	hexane	0.1	RT	7	85	8.9:1.0
10	1.5	hexane	0.1	55	7	70	9.2:1.0
11	1.5	hexane	0.05	RT	9	79	9.0:1.0
12	1.1	hexane	0.1	RT	7	59	8.6:1.0

[a]Yields and ratio (*Z/E*) of two isomers were determined by ^{19}F NMR analysis of crude reaction mixture.

Supplementary References

1. Wang, J., Ogawa, Y. & Shibata, N. Activation of Saturated Fluorocarbons to Synthesize Spirobiindanes, Monofluoroalkenes, and Indane Derivatives. *iScience* **17**, .132-143 (2019).
2. Lan, K., Shan, Z. & Fan, S. Synthesis of spirobiindanes via bis-cyclization reaction of the 1,5-diaryl-3-pentanones catalyzed by heteropoly acids. *Tetrahedron Lett.* **47**, 4343-4345 (2006).
3. Saputra, M. A., Ngo, L. & Kartika, R. Synthesis of Vinyl Chlorides via Triphosgene–Pyridine Activation of Ketones. *J. Org. Chem.* **80**, 8815-8820 (2015).
4. Mukaiyama, T., Hayashi, M. & Narasaka, K. Convenient method for the reduction of sulfides and vinylic or aromatic halides by using titanium chloride and lithium aluminum hydride. *Chem. Lett.* **3**, 291-294 (1973).
5. Ebule, R., Liang, S., Hammond, G. B. & Xu, B. Chloride-Tolerant Gold(I)-Catalyzed Regioselective Hydrochlorination of Alkynes. *ACS Catal.* **7**, 6798-6801 (2017).
6. Nagendrappa, G. Synthesis of 1-Trimethylsilylcycloalkenes from 1-Bromocycloalkenes by Wurtz-type Coupling. *Synthesis*, 704-706 (1980).
7. Nahra, F. et al. Hydrofluorination of Alkynes Catalysed by Gold Bifluorides. *ChemCatChem* **7**, 240-244 (2015).
8. Vandamme, M. & Paquin, J.-F. Eliminative Deoxofluorination Using XtalFluor-E: A One-Step Synthesis of Monofluoroalkenes from Cyclohexanone Derivatives. *Org. Lett.* **19**, 3604-3607 (2017).

Supplementary Figure 1. ^{1}H NMR spectrum of unknown compounds **2e**, related to **Figure 2**

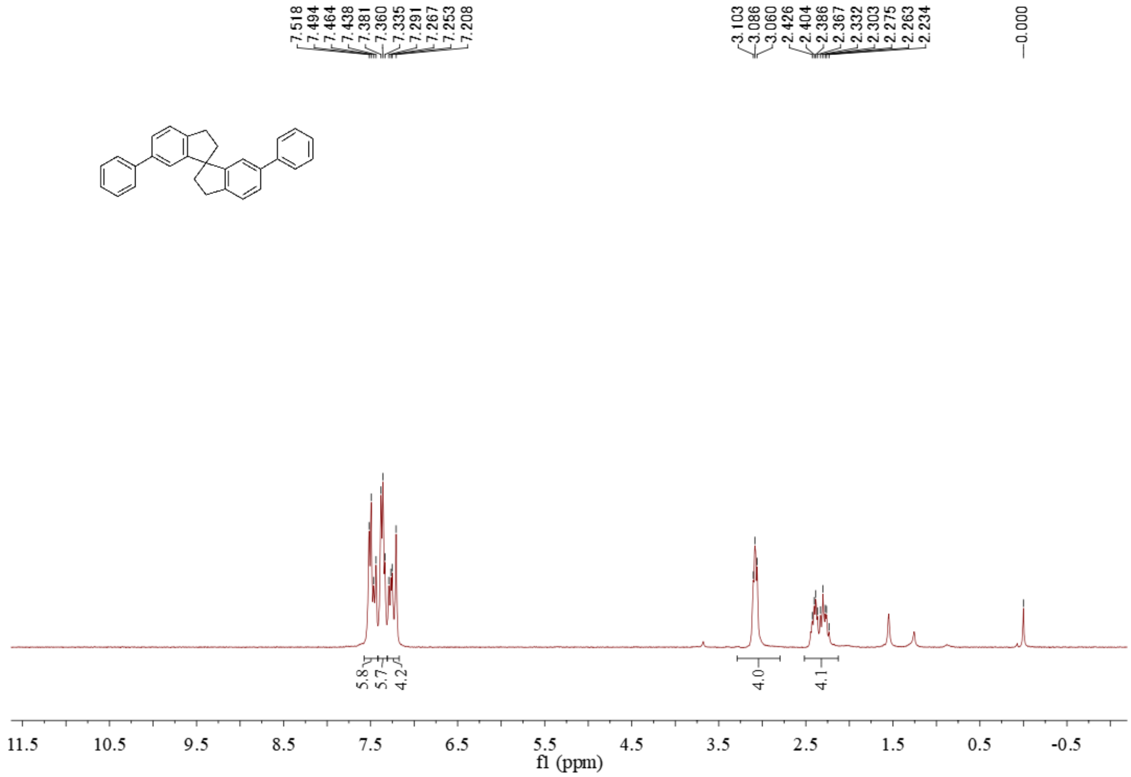

Supplementary Figure 2. ^{13}C NMR spectrum of unknown compounds **2e**, related to **Figure 2**

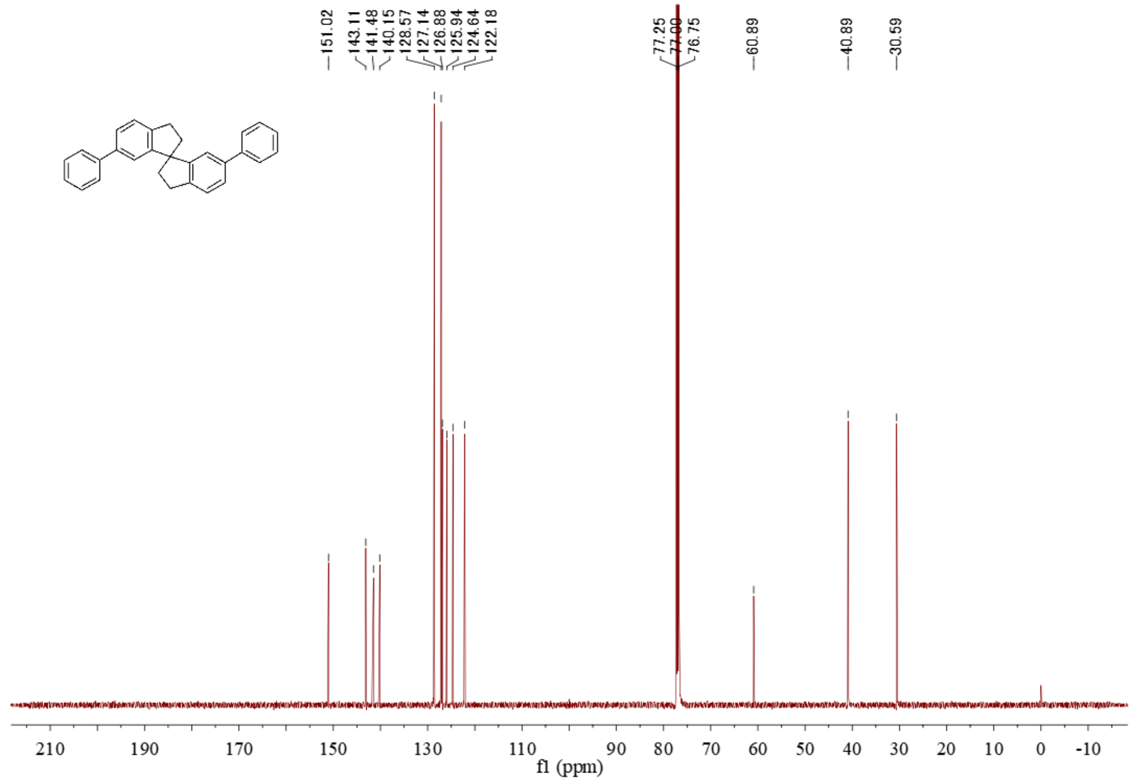

Supplementary Figure 3. ^{1}H NMR spectrum of unknown compounds **2j**, related to **Figure 2**

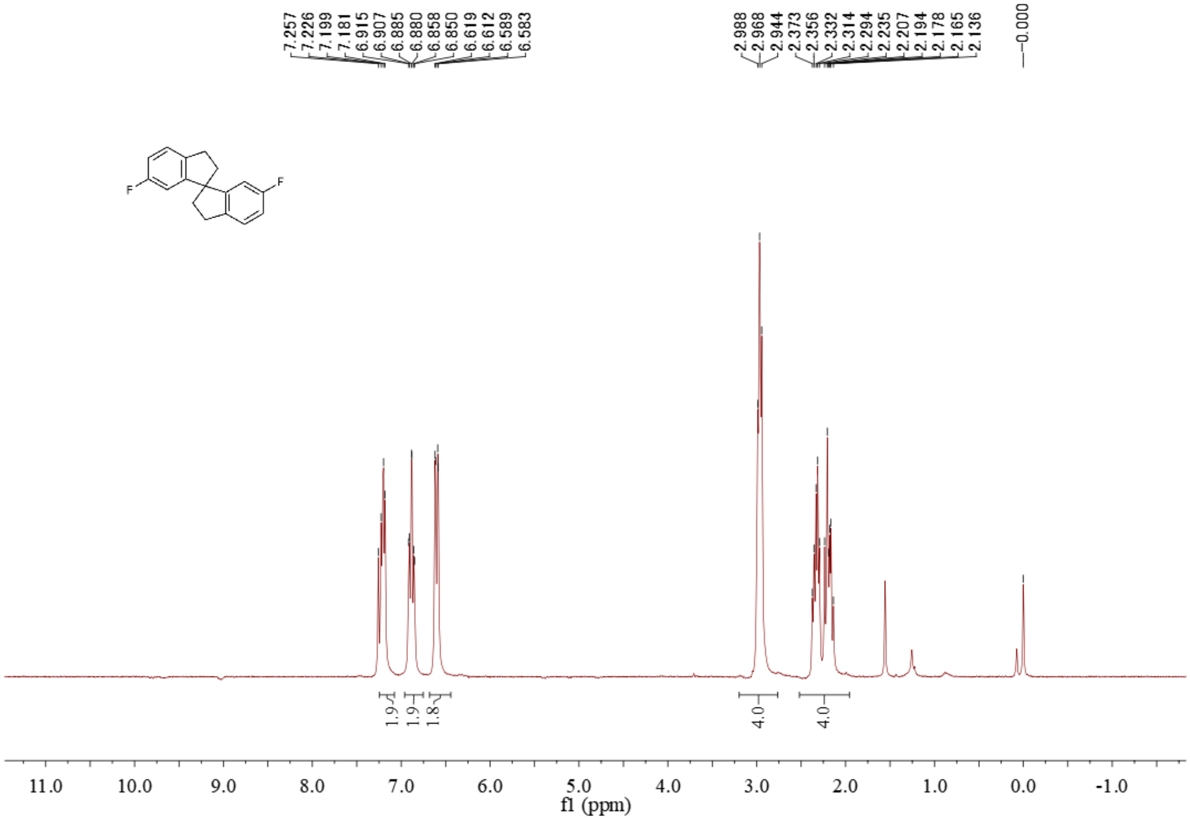

Supplementary Figure 4. ^{13}C NMR spectrum of unknown compounds **2j**, related to **Figure 2**

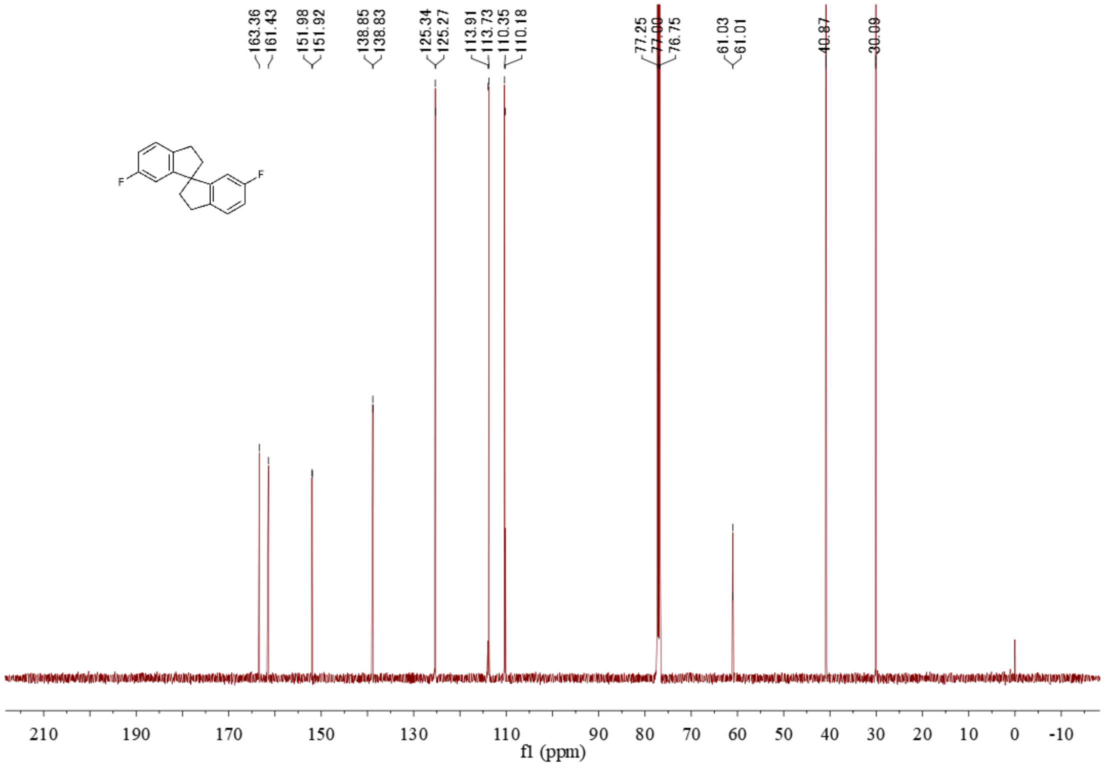

Supplementary Figure 5. ^{1}H NMR spectrum of unknown compounds **3g**, related to **Figure 2**

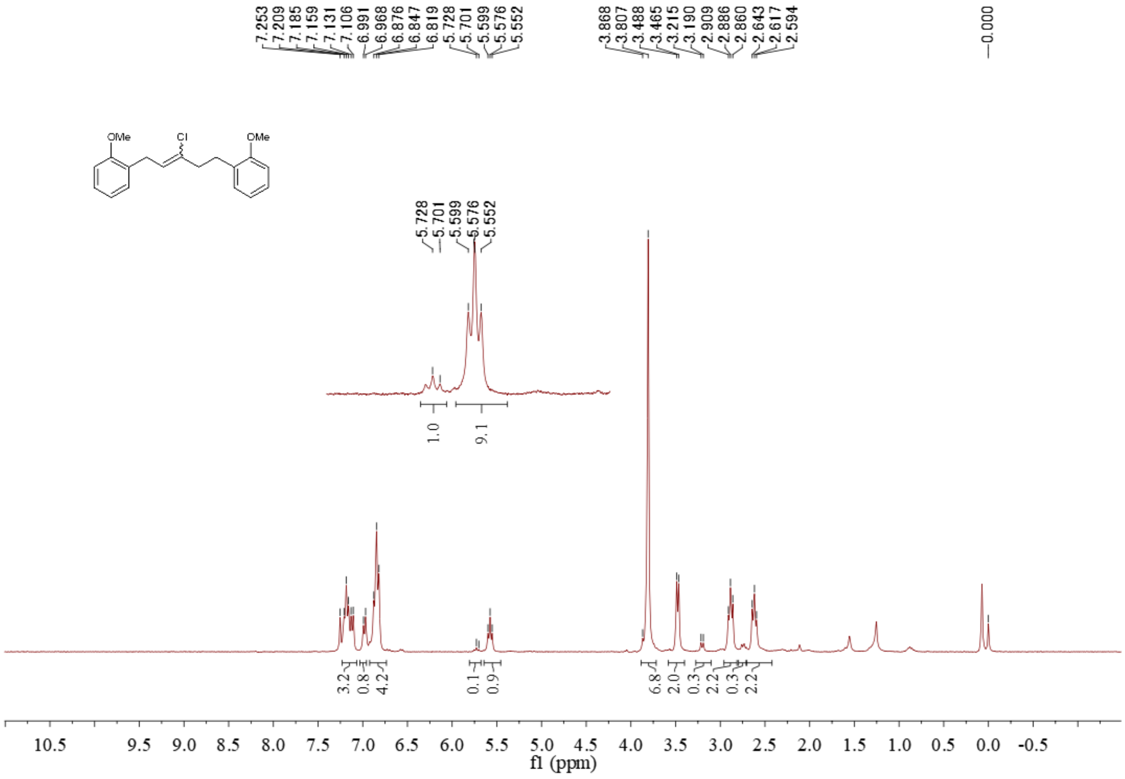

Supplementary Figure 6. ^{13}C NMR spectrum of unknown compounds **3g**, related to **Figure 2**

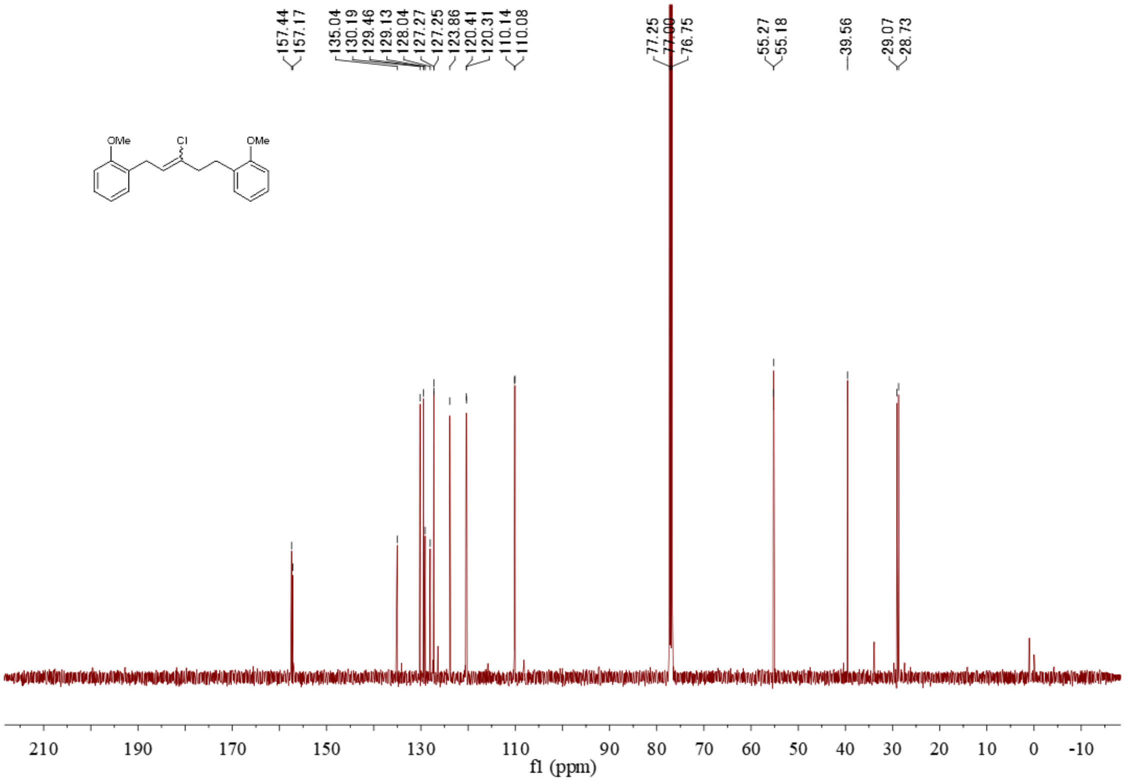

Supplementary Figure 7. ^{1}H NMR spectrum of known compounds **2a**, related to **Figure 2**

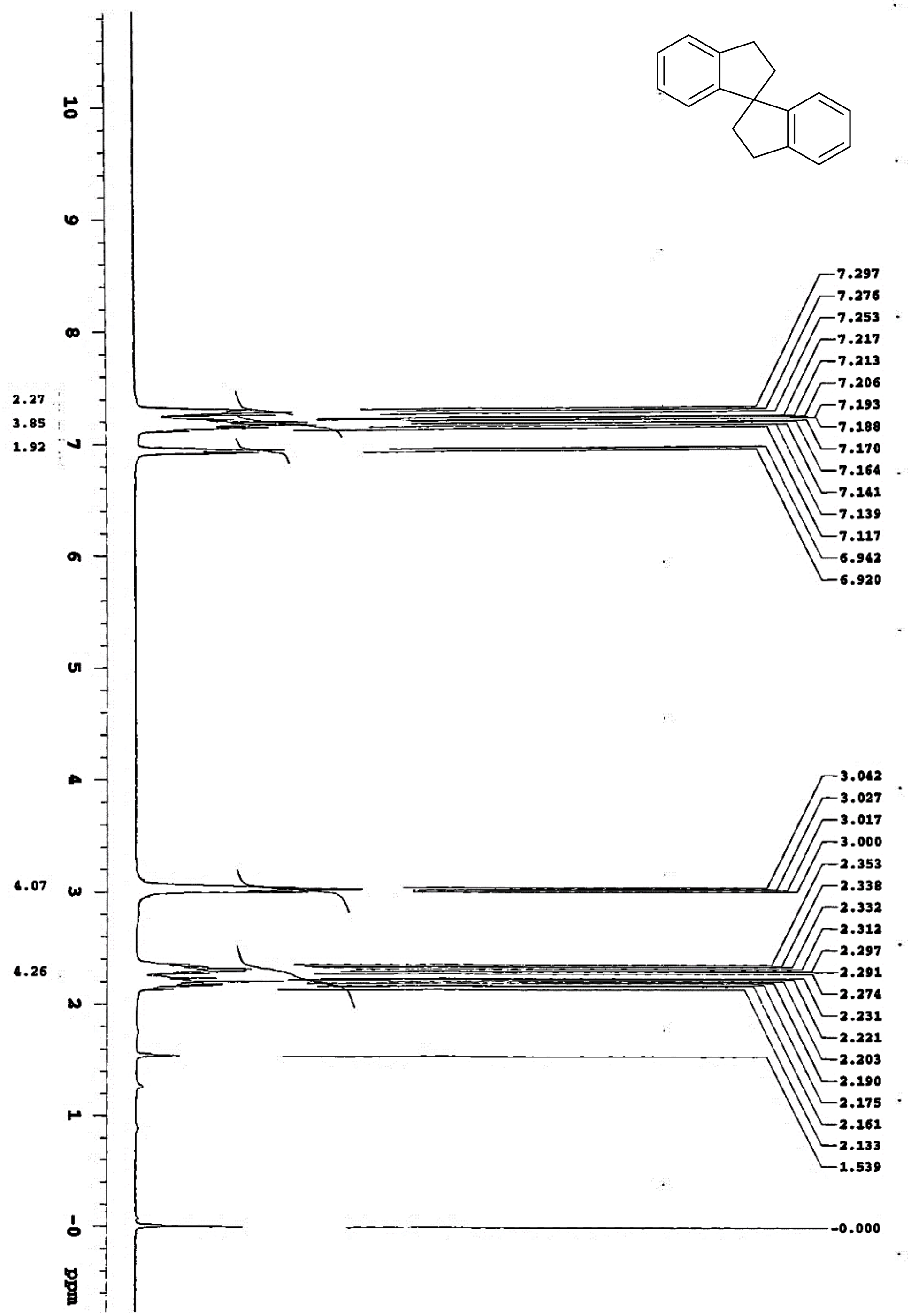

Supplementary Figure 8. ^{1}H NMR spectrum of known compounds **2b**, related to **Figure 2**

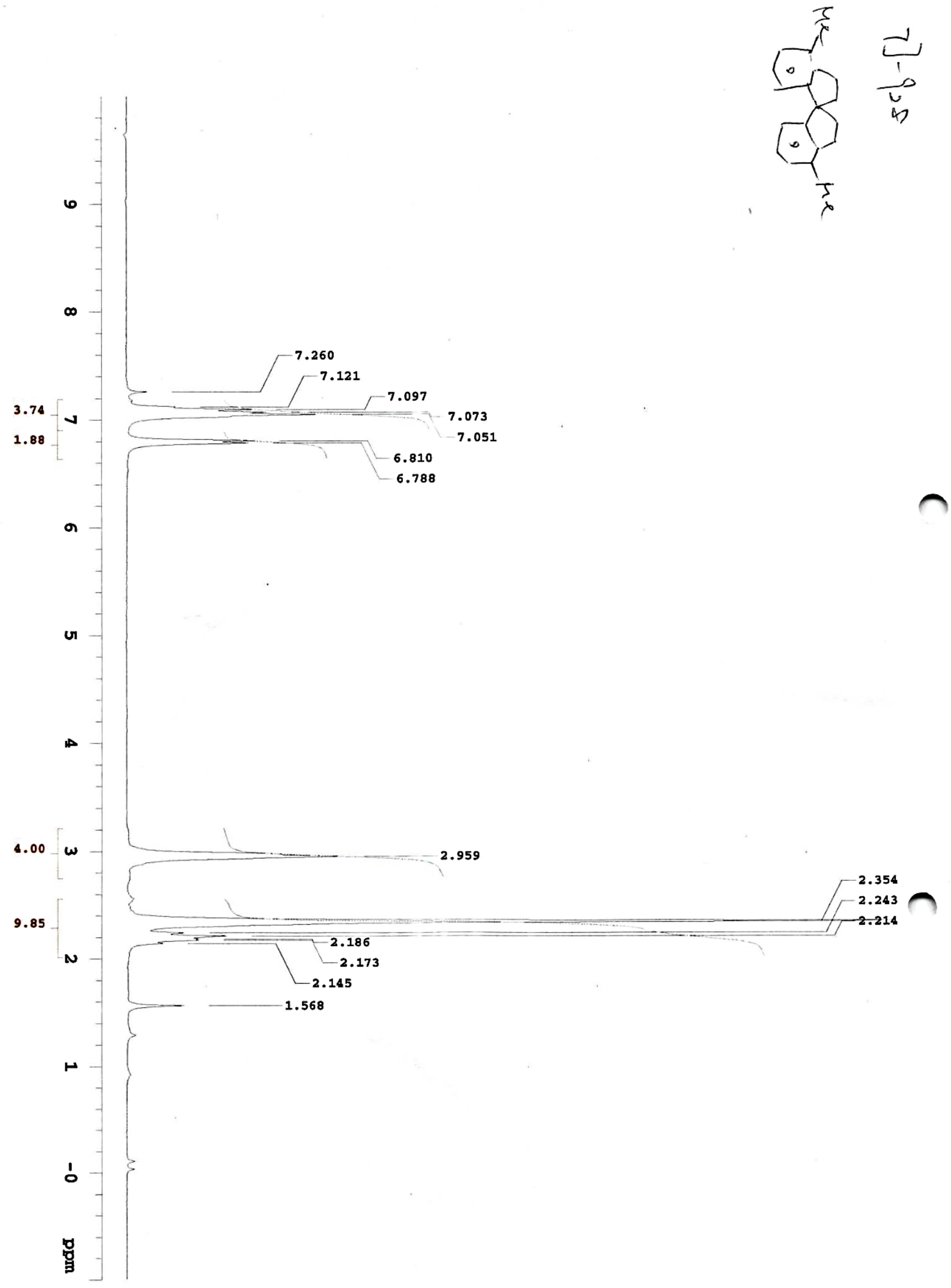

Supplementary Figure 9. ^{1}H NMR spectrum of known compounds **2c**, related to **Figure 2**

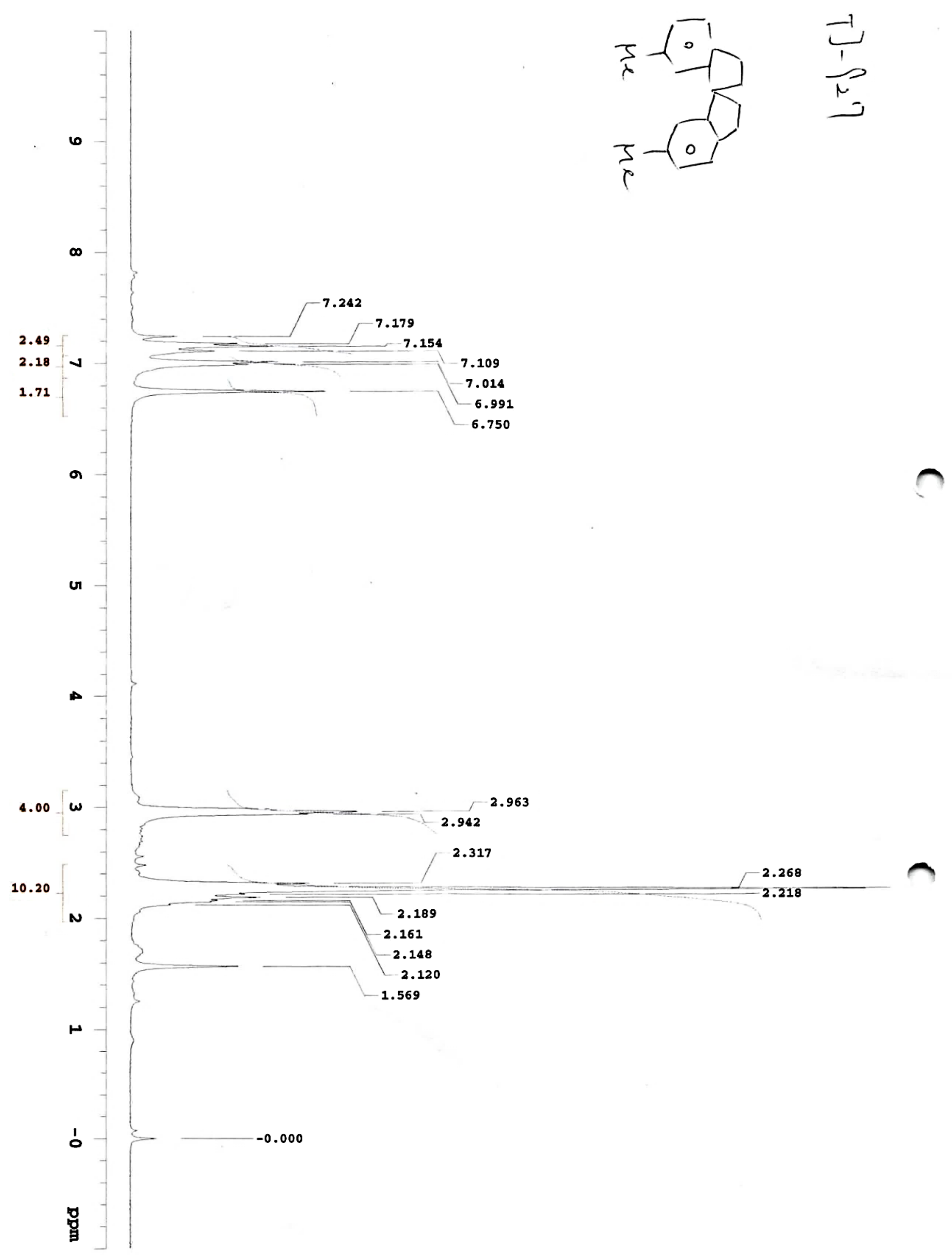

Supplementary Figure 10. ^{1}H NMR spectrum of known compounds **2d**, related to **Figure 2**

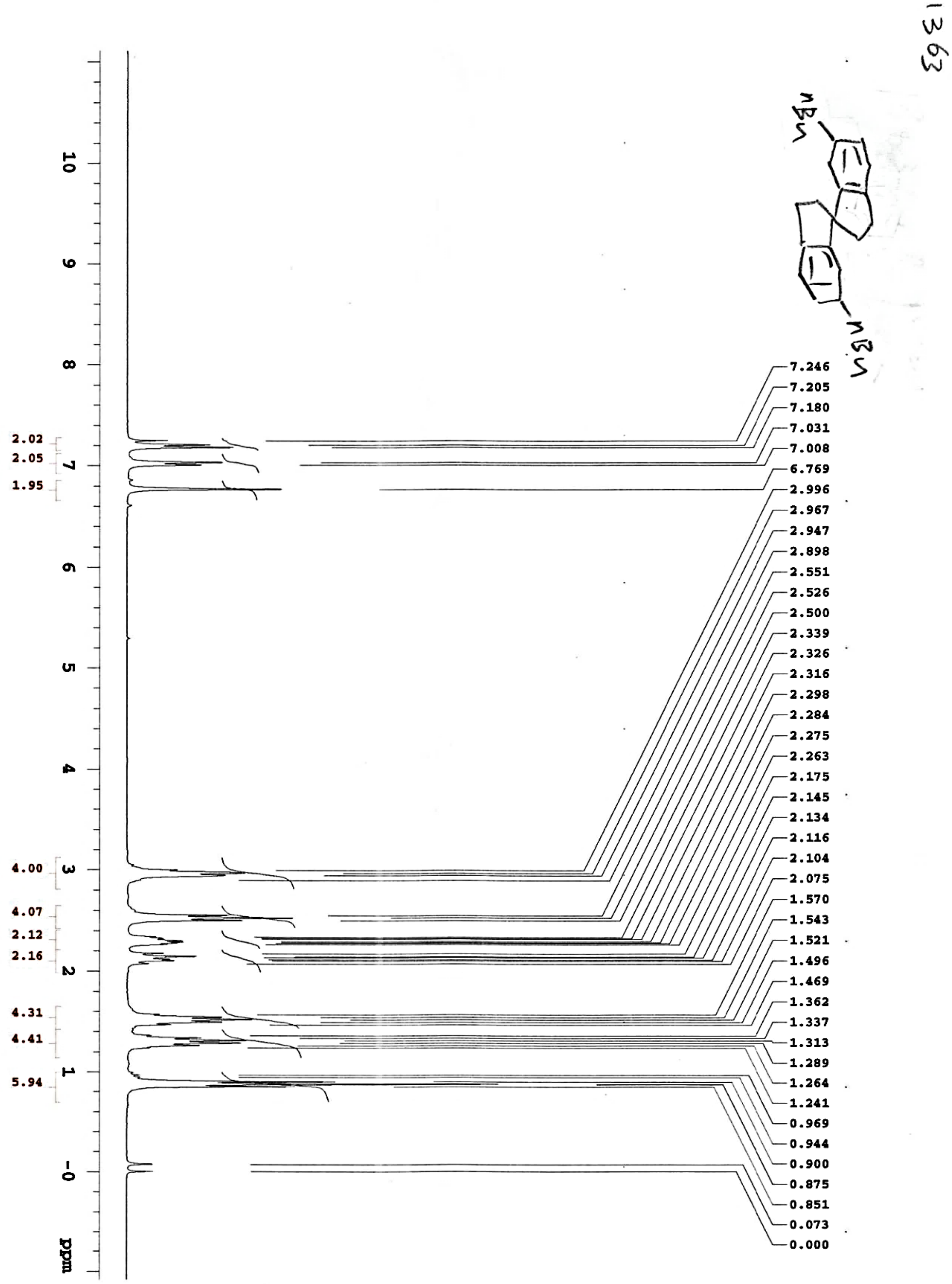

Supplementary Figure 11. ^{1}H NMR spectrum of known compounds **2f**, related to **Figure 2**

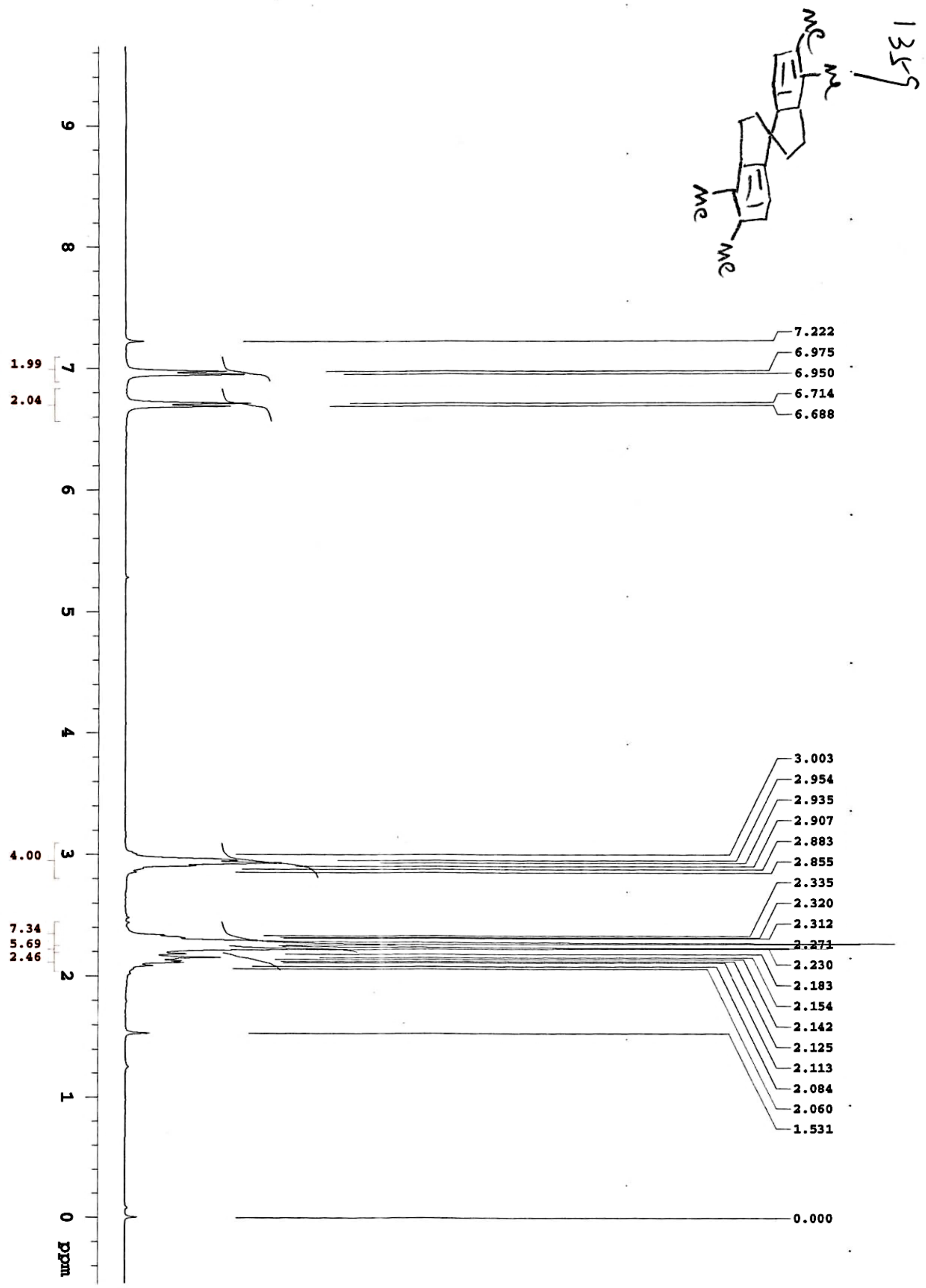

Supplementary Figure 12. ^{1}H NMR spectrum of known compounds **2h**, related to **Figure 2**

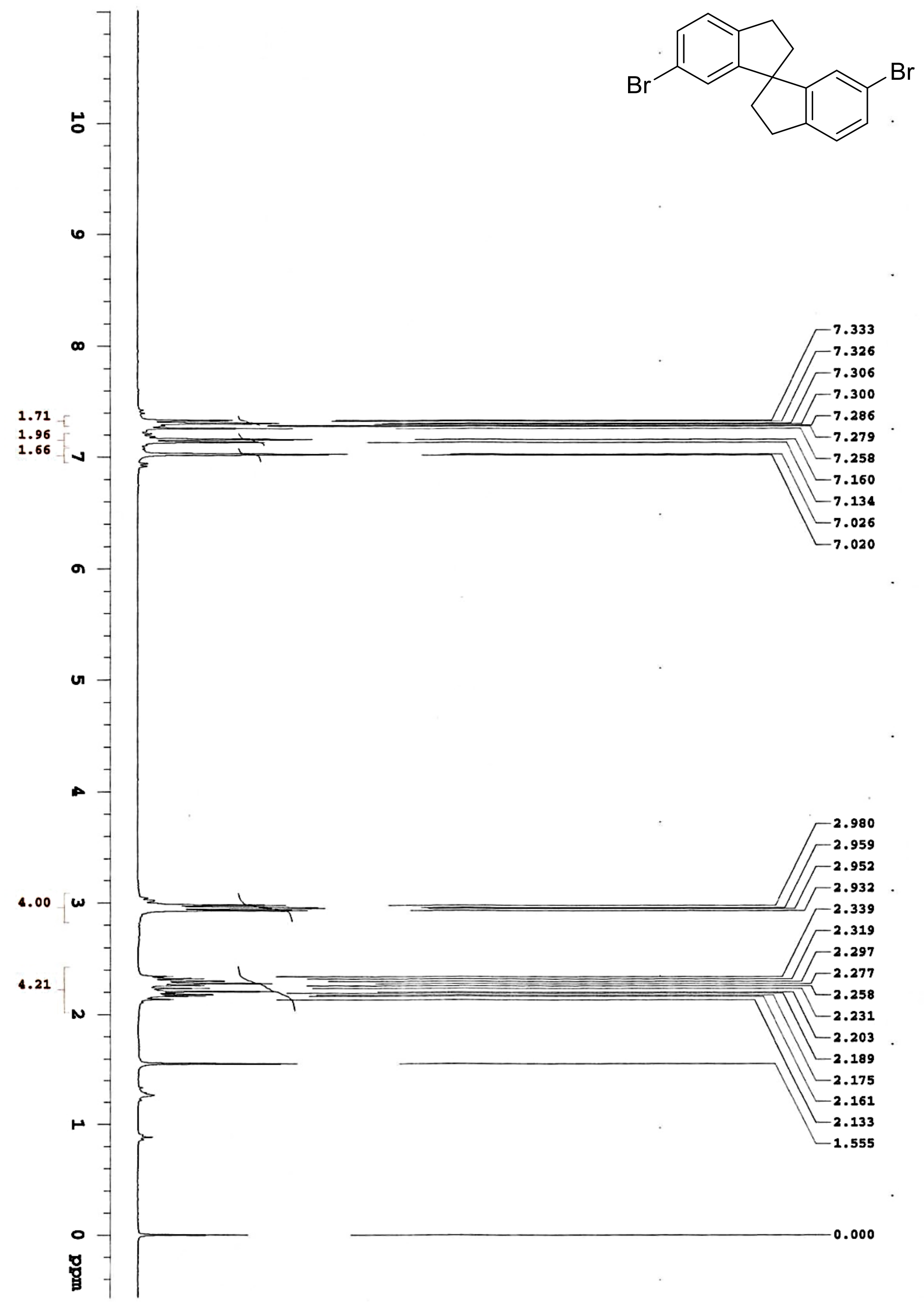

Supplementary Figure 13. ^{1}H NMR spectrum of known compounds **2i**, related to **Figure 2**

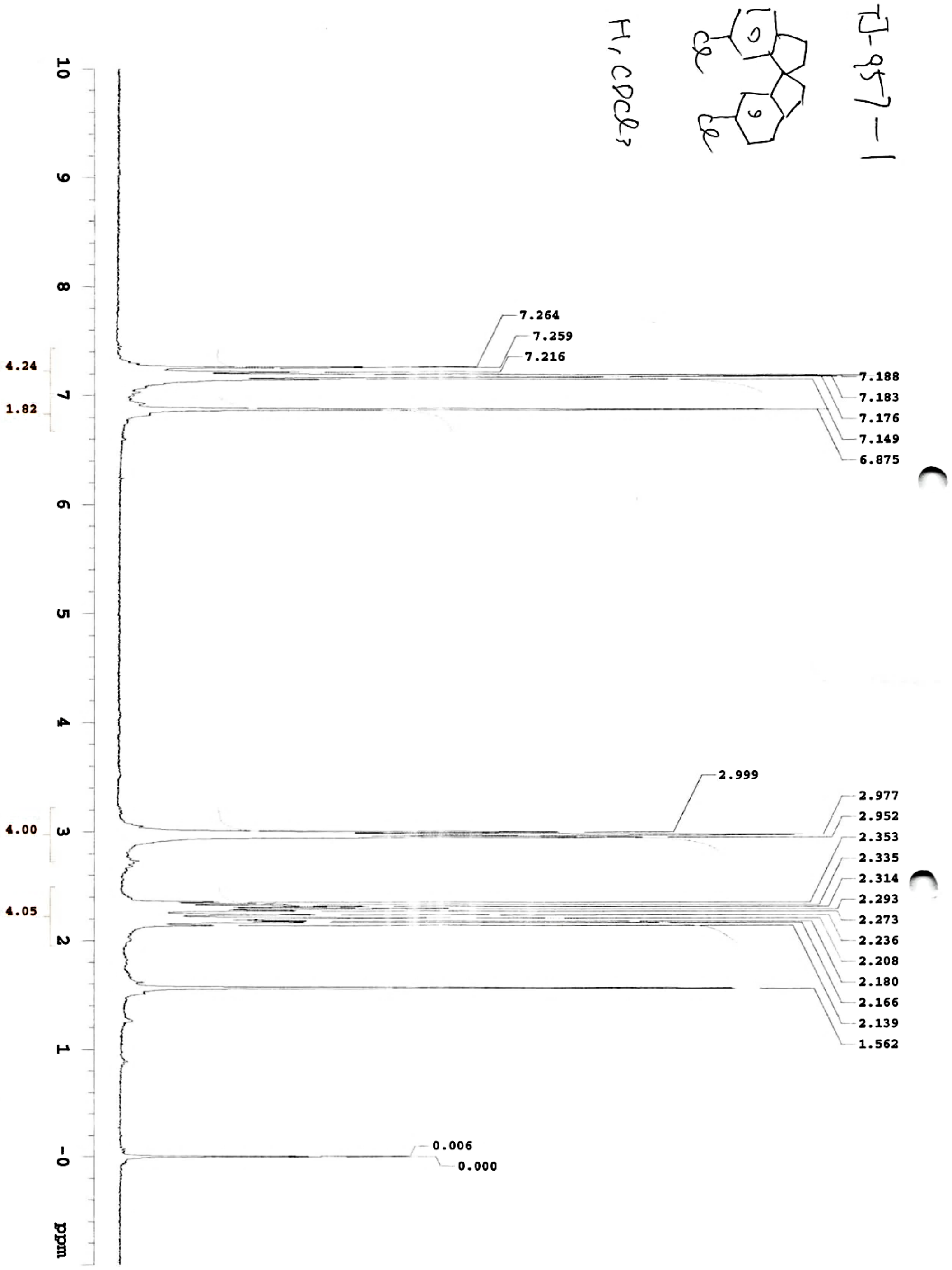

Supplementary Figure 14. ^{1}H NMR spectrum of known compounds **2k**, related to **Figure 2**

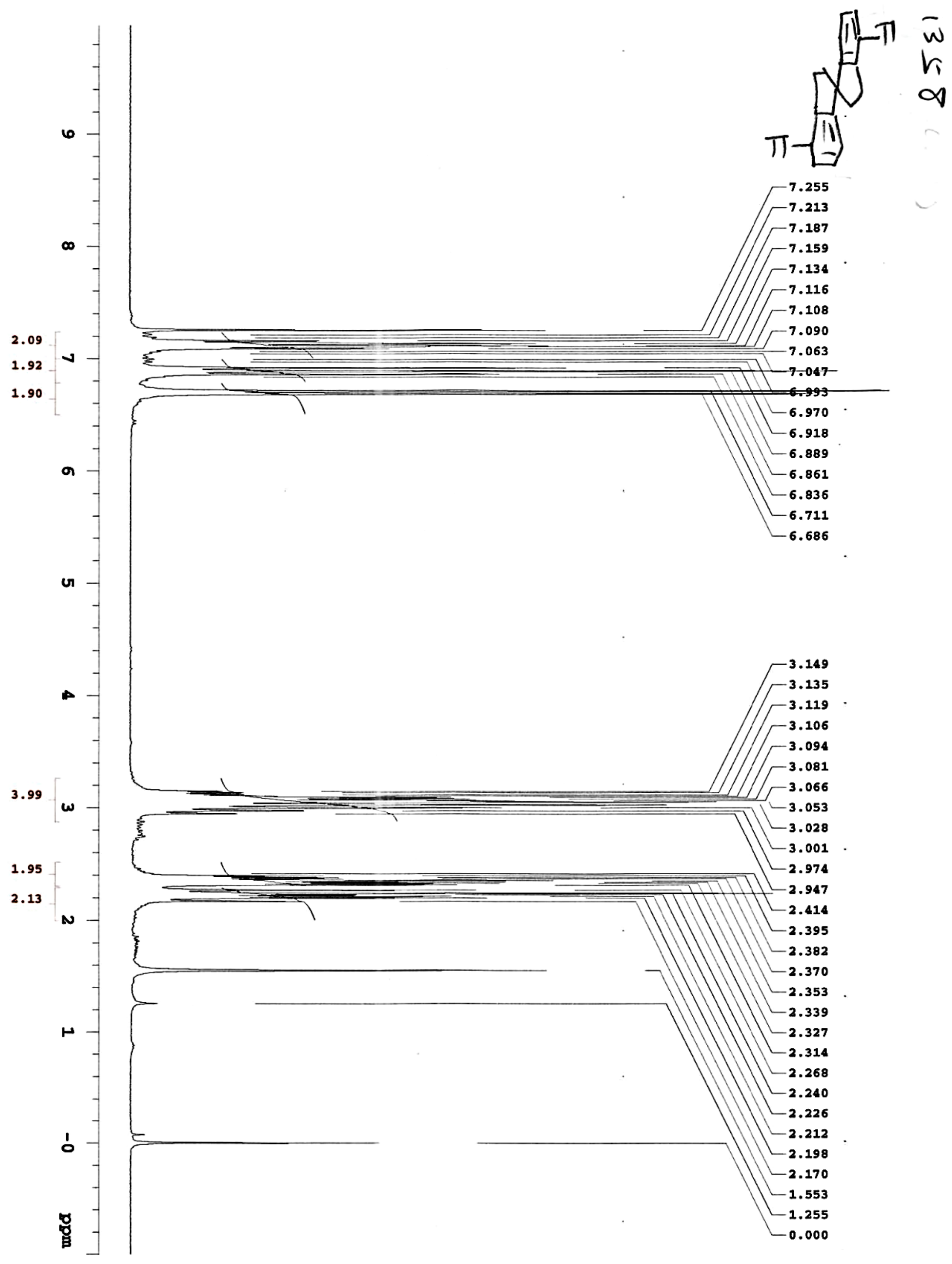

Supplementary Figure 15. ^{1}H NMR spectrum of known compounds **2l**, related to **Figure 2**

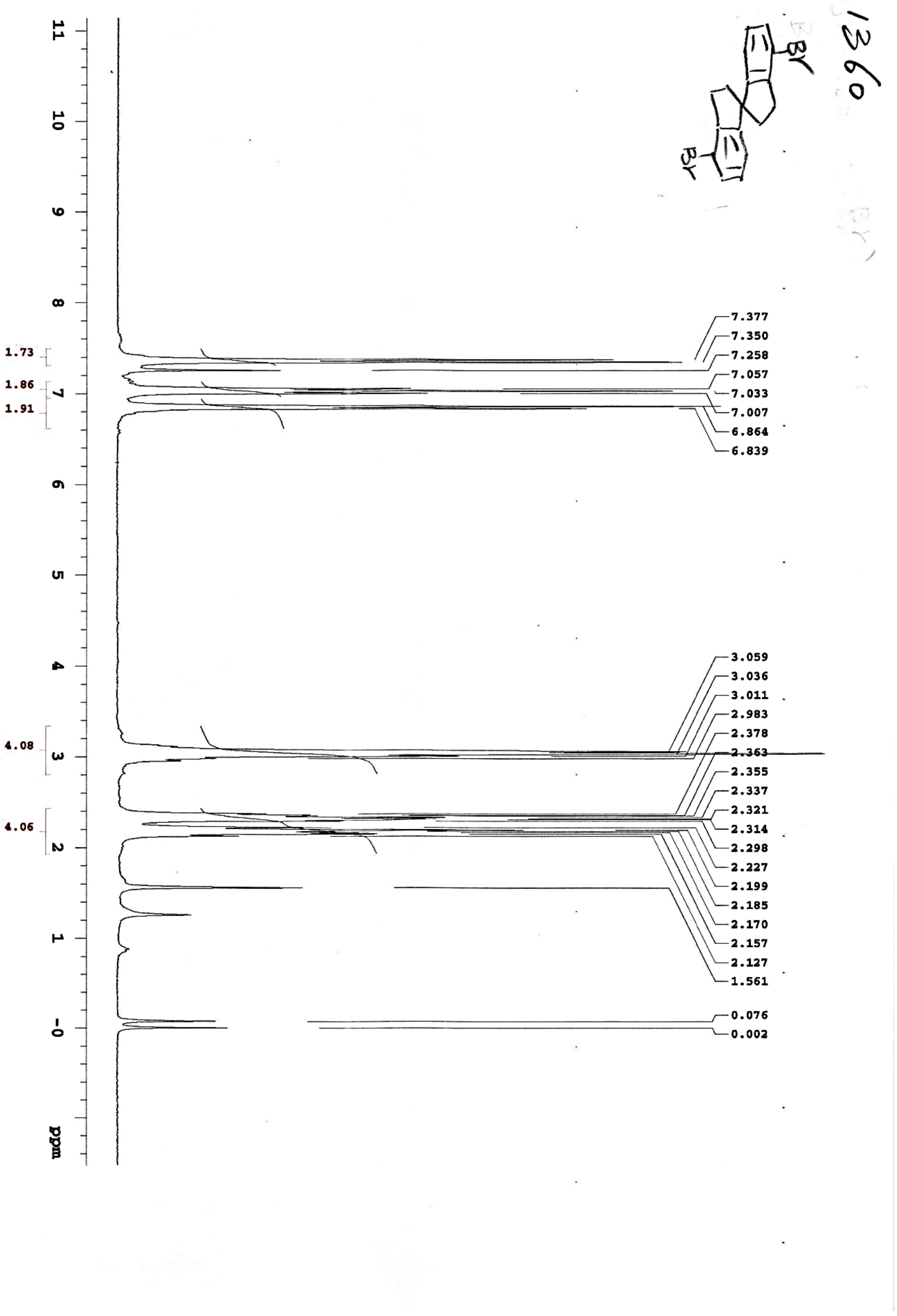

Supplementary Figure 16. ^{1}H NMR spectrum of known compounds **2m**, related to **Figure 2**

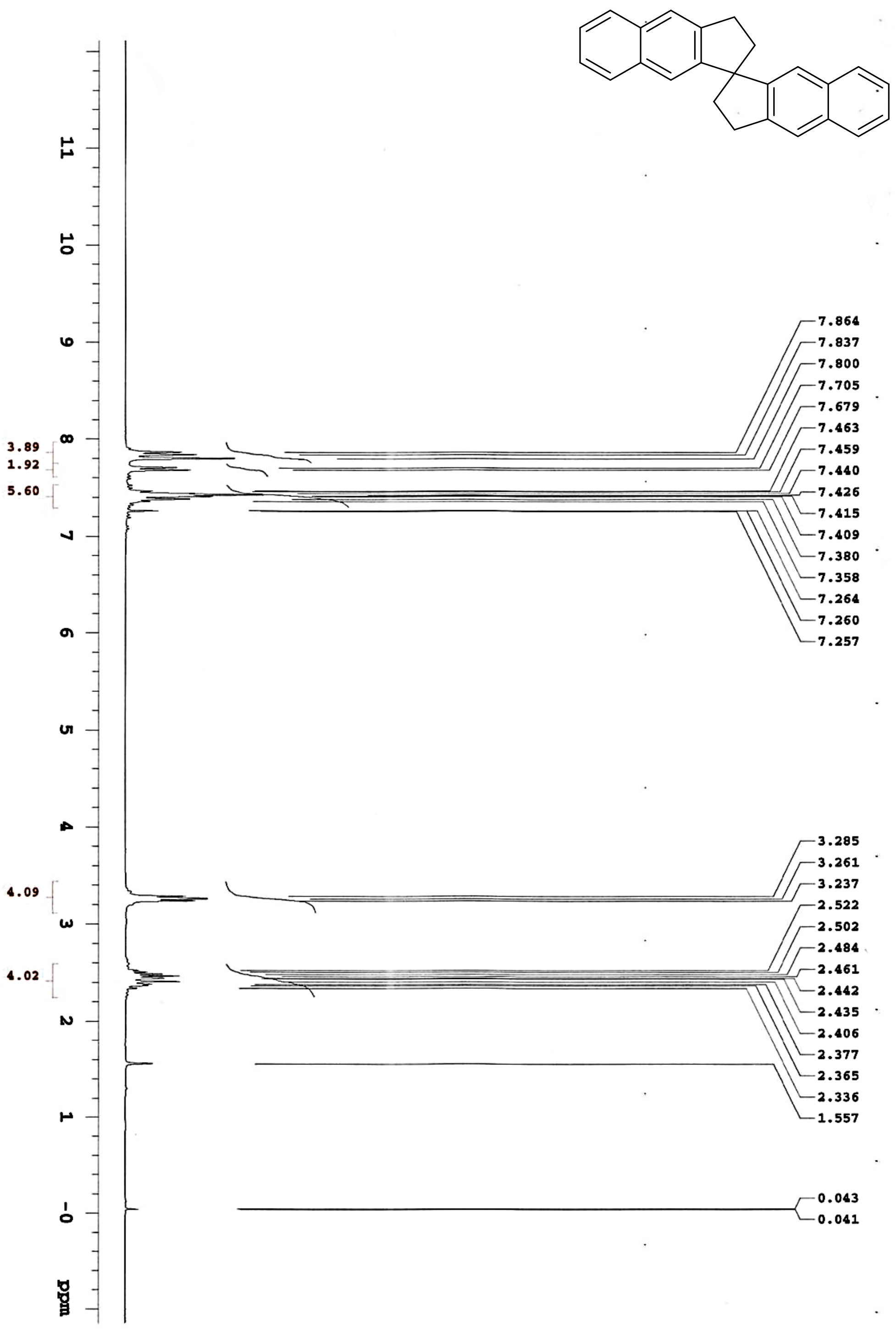

Supplementary Figure 17. ^{1}H NMR spectrum of known compounds **2n**, related to **Figure 2**

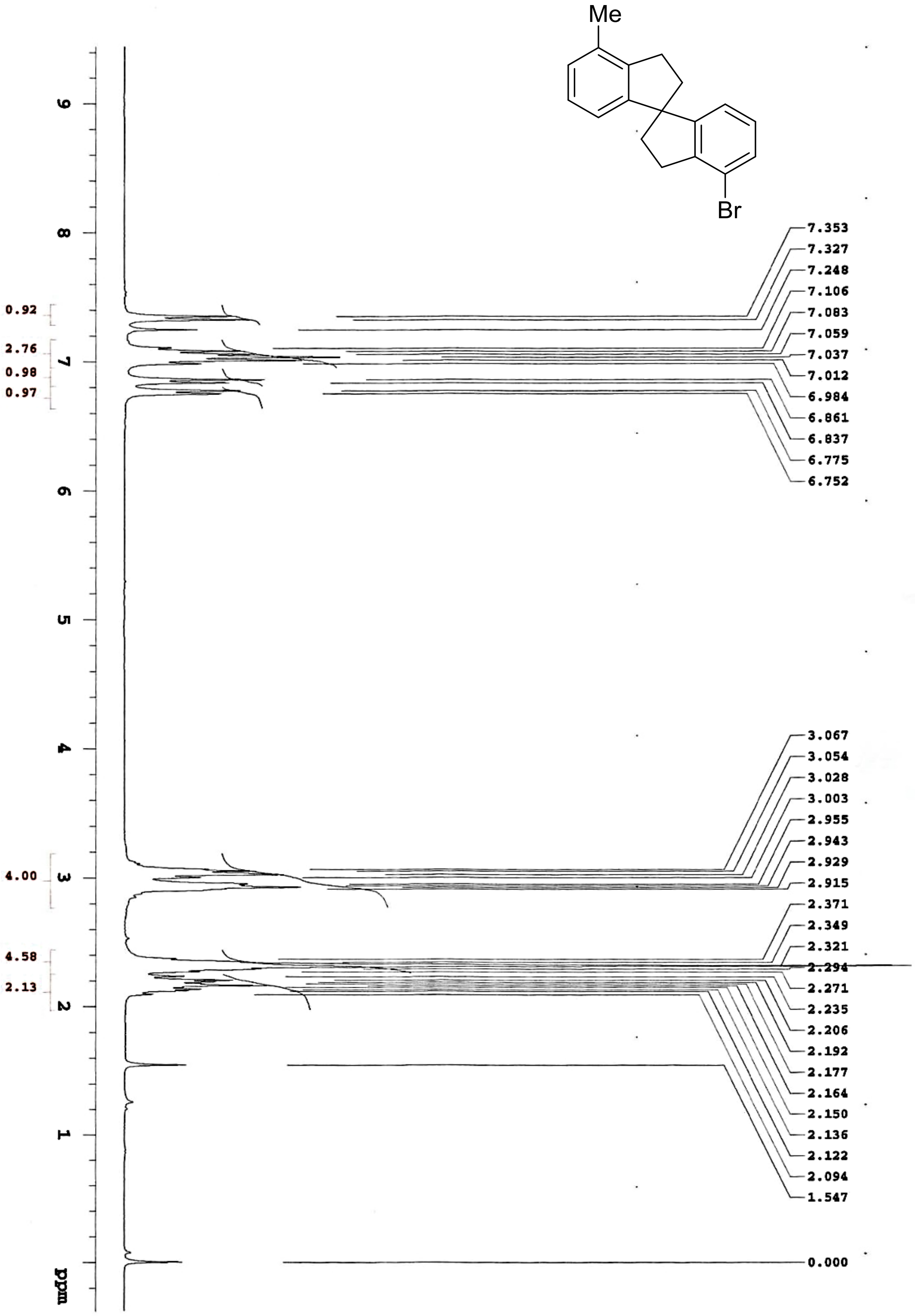

Supplementary Figure 18. ^{1}H NMR spectrum of known compounds **2o**, related to **Figure 2**

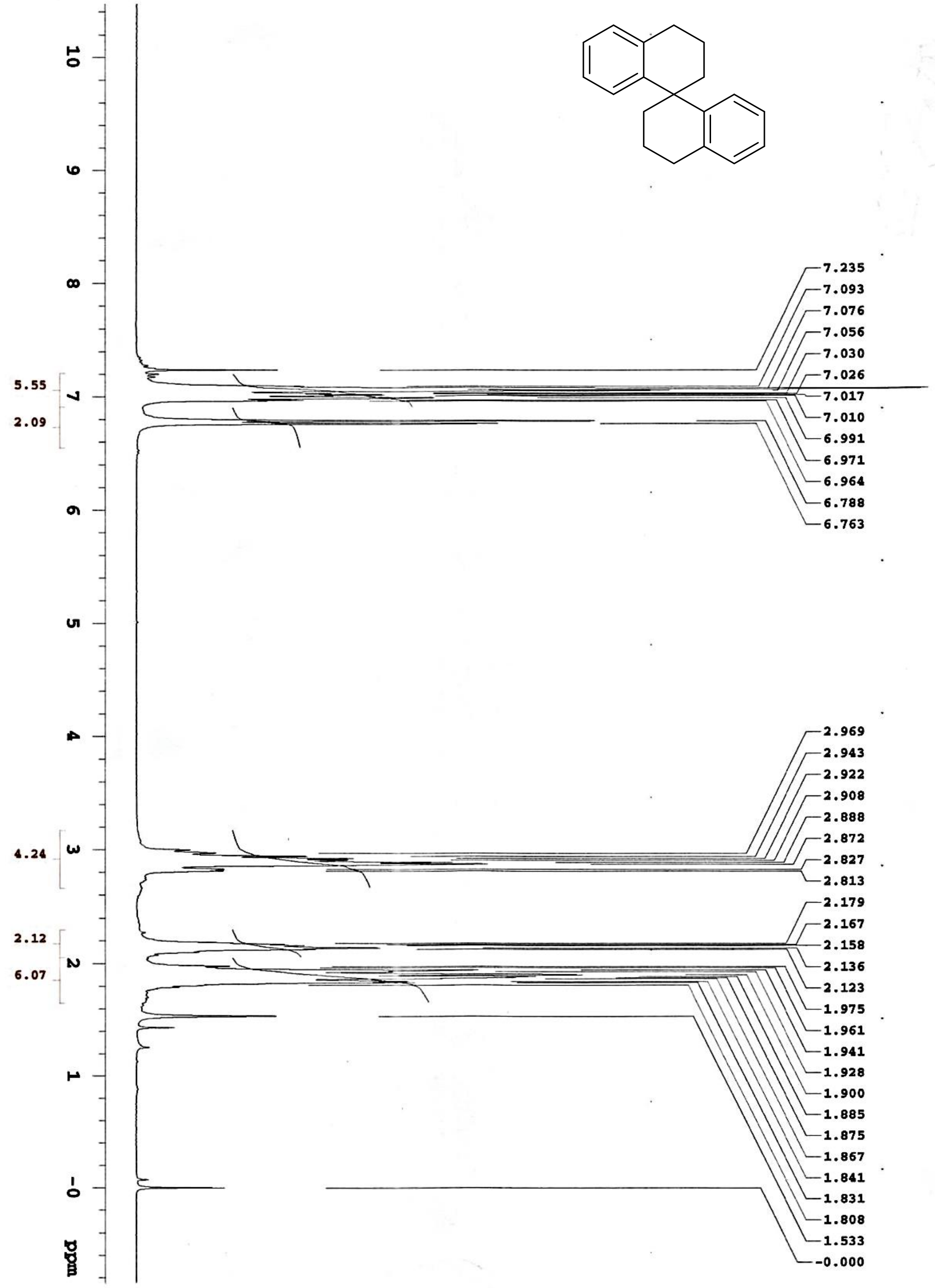

Supplementary Figure 19. ^{1}H NMR spectrum of unknown compounds **3b**, related to **Figure 3**

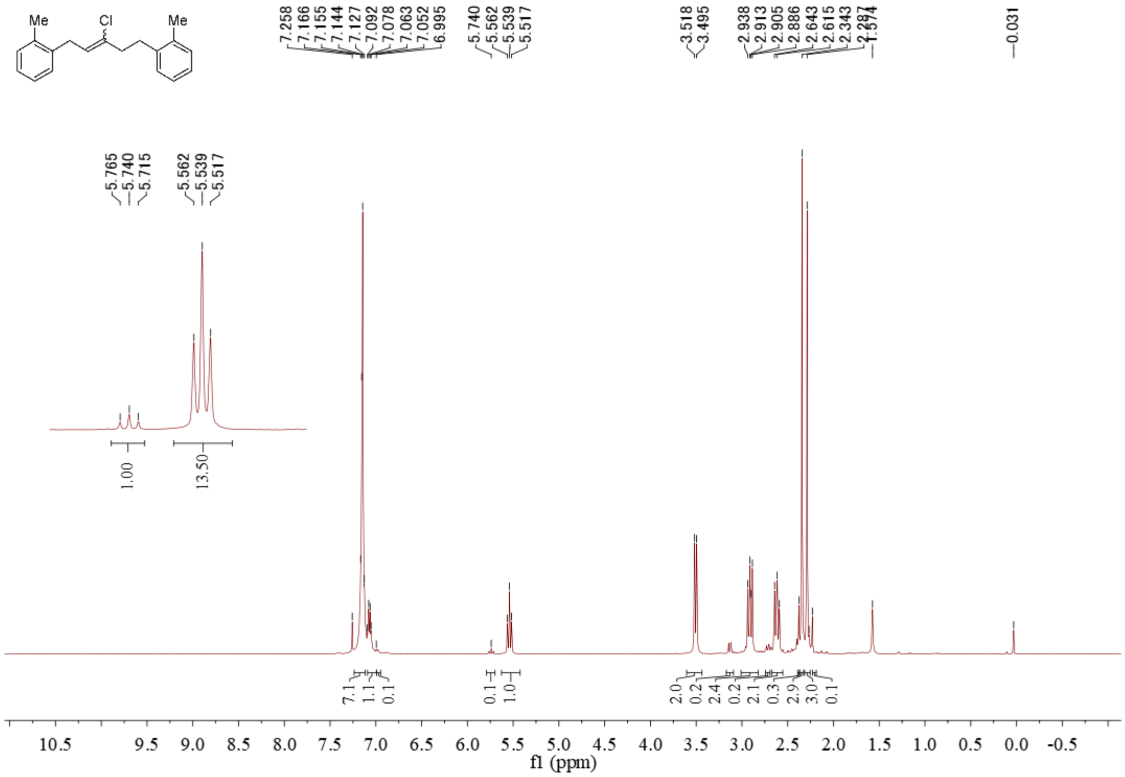

Supplementary Figure 20. ^{13}C NMR spectrum of unknown compounds **3b**, related to **Figure 3**

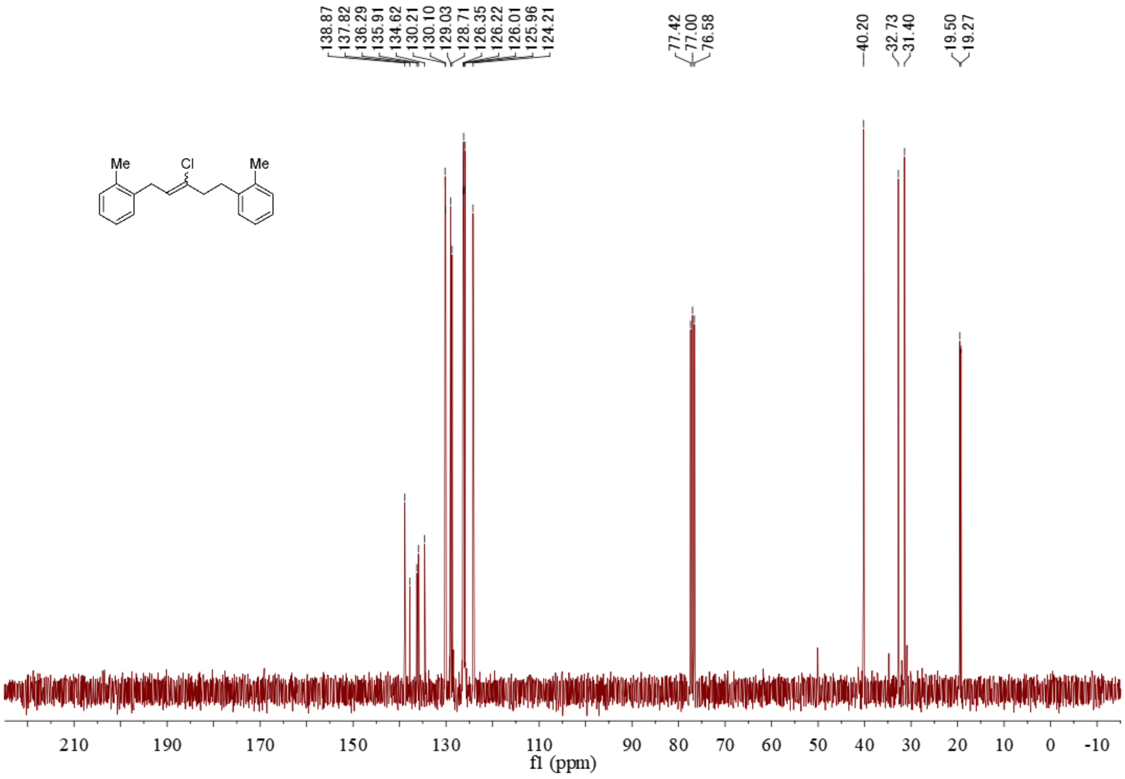

Supplementary Figure 21. ^{1}H NMR spectrum of unknown compounds **3p**, related to **Figure 3**

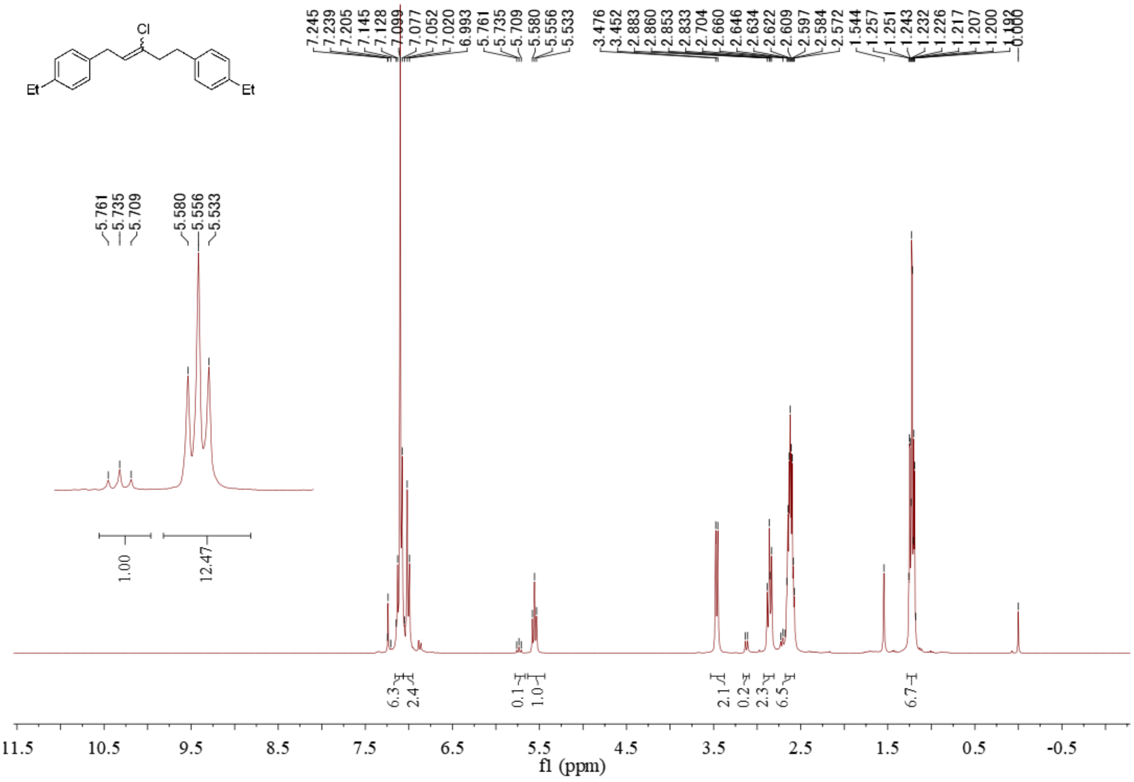

Supplementary Figure 22. ^{13}C NMR spectrum of unknown compounds **3p**, related to **Figure 3**

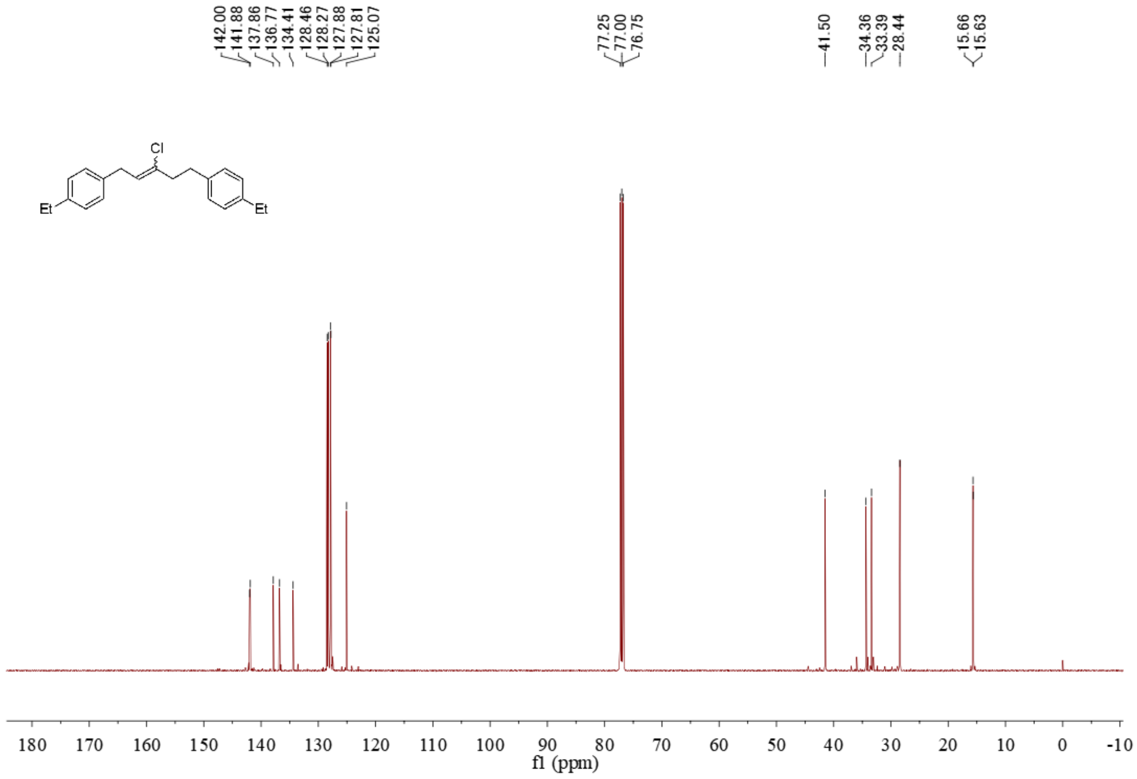

Supplementary Figure 23. ^{1}H NMR spectrum of unknown compounds **3d**, related to **Figure 3**

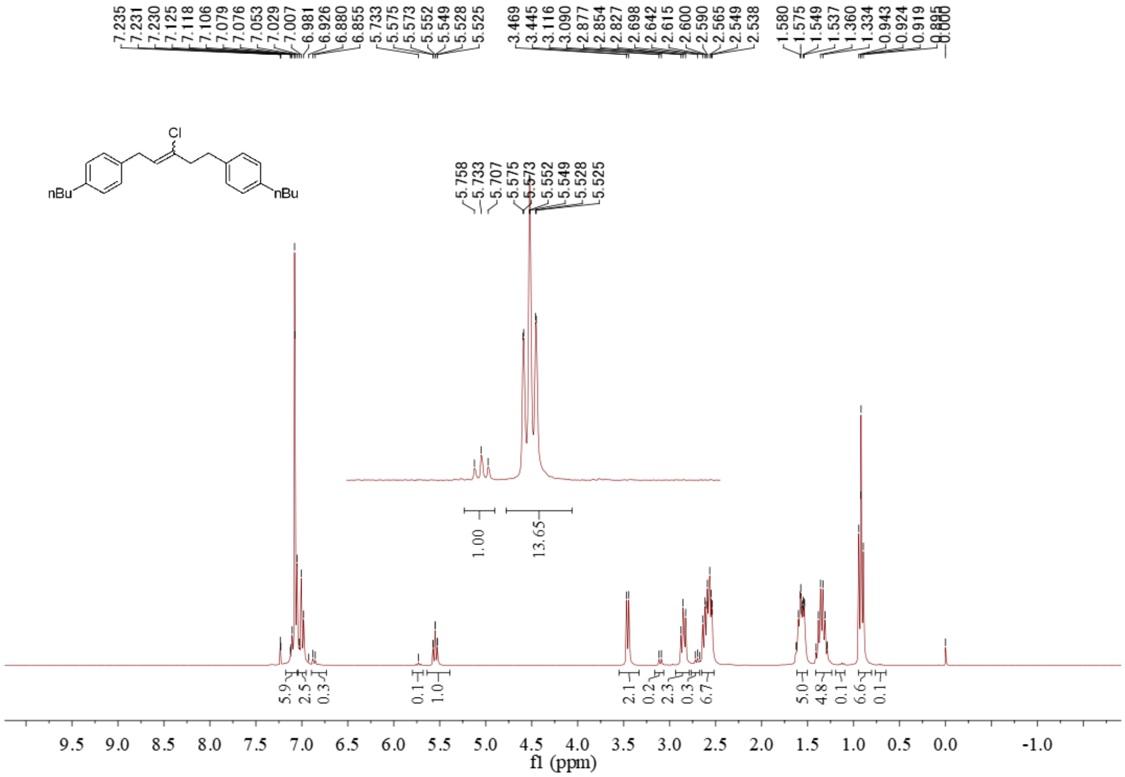

Supplementary Figure 24. ^{13}C NMR spectrum of unknown compounds **3d**, related to **Figure 3**

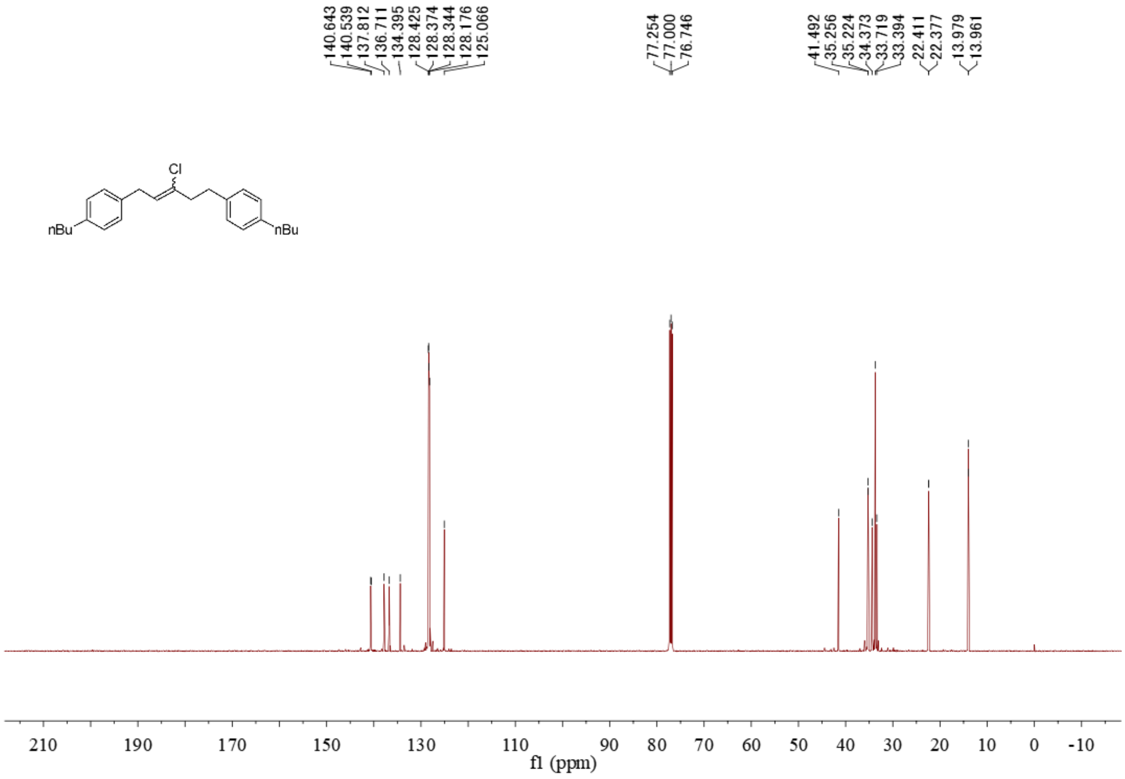

Supplementary Figure 25. ^{1}H NMR spectrum of unknown compounds **3h**, related to **Figure 3**

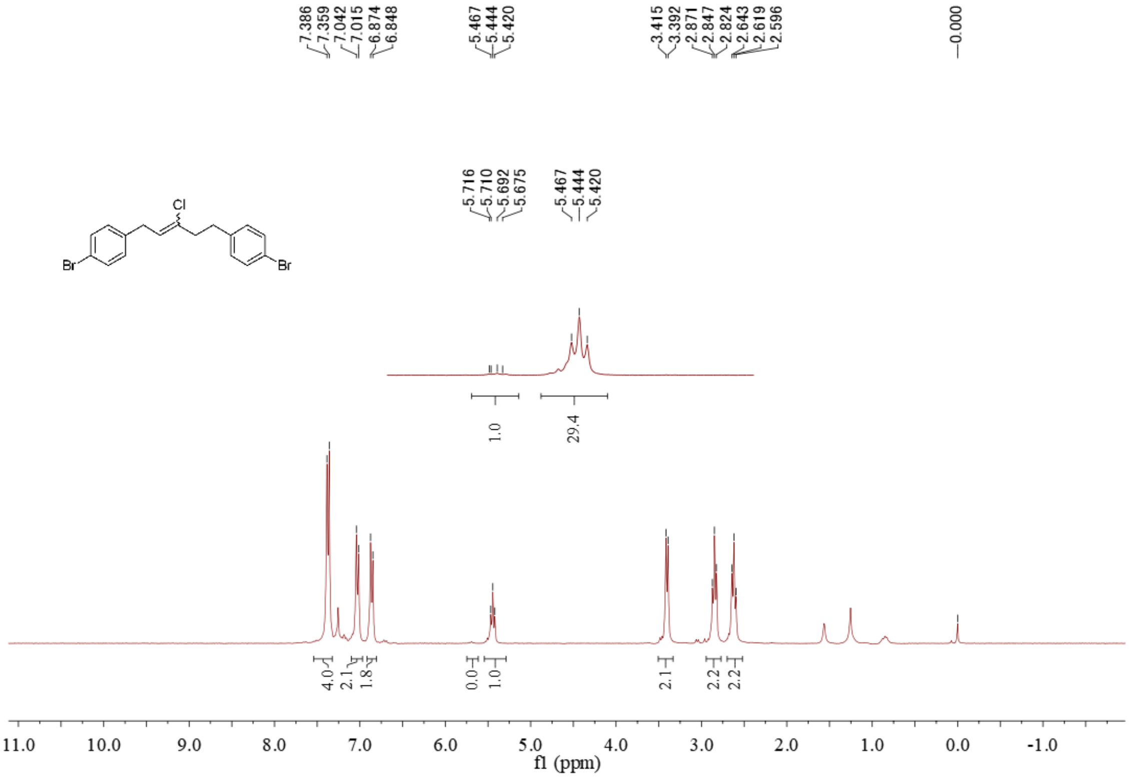

Supplementary Figure 26. ^{13}C NMR spectrum of unknown compounds **3h**, related to **Figure 3**

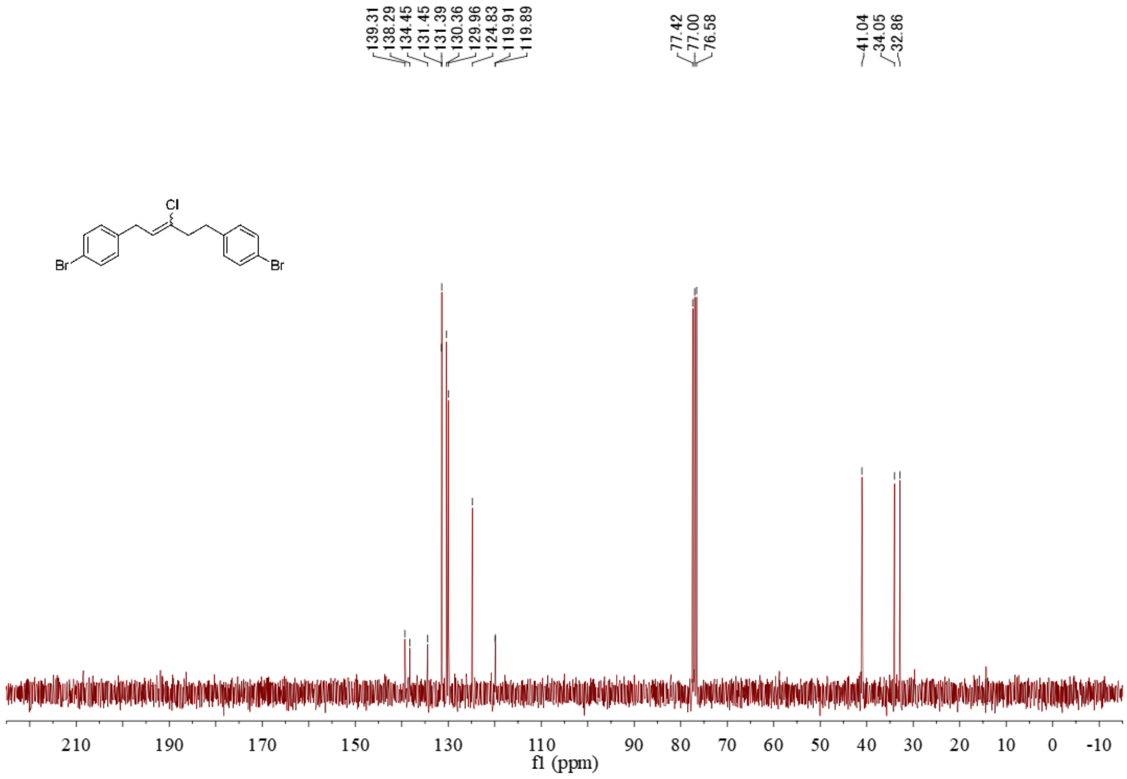

Supplementary Figure 27. ^{1}H NMR spectrum of unknown compounds **3i**, related to **Figure 3**

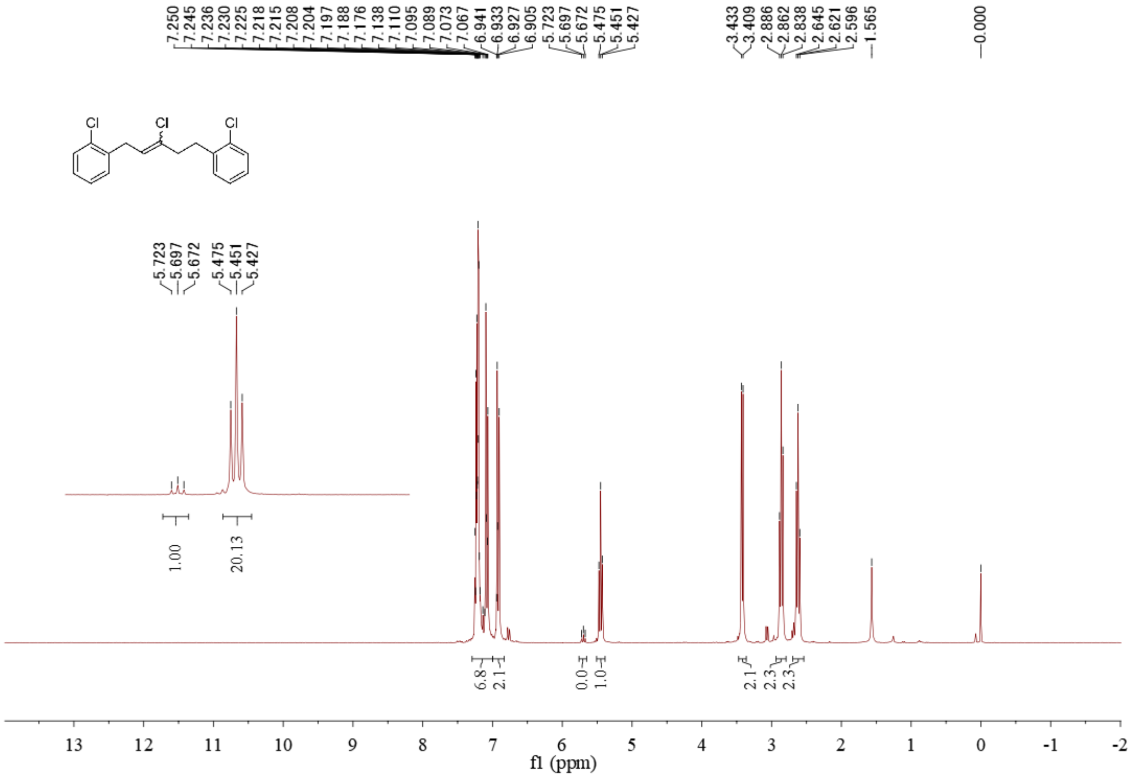

Supplementary Figure 28. ^{13}C NMR spectrum of unknown compounds **3i**, related to **Figure 3**

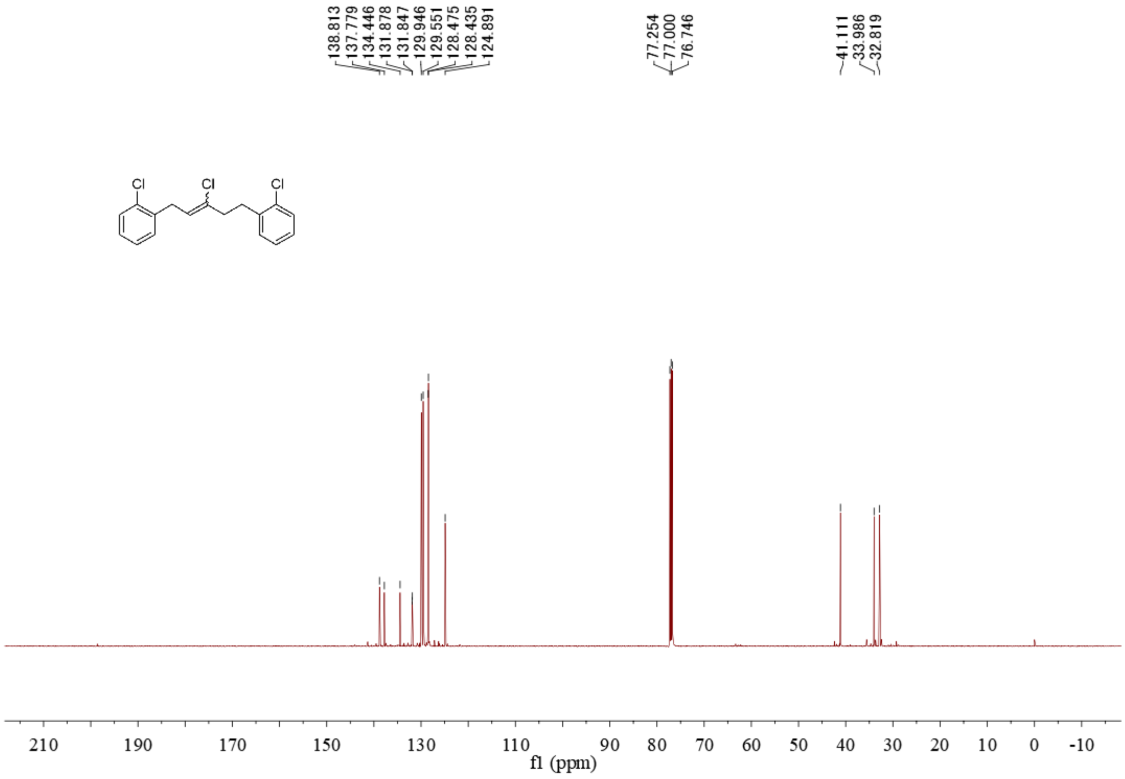

Supplementary Figure 29. ^{1}H NMR spectrum of unknown compounds **3k**, related to **Figure 3**

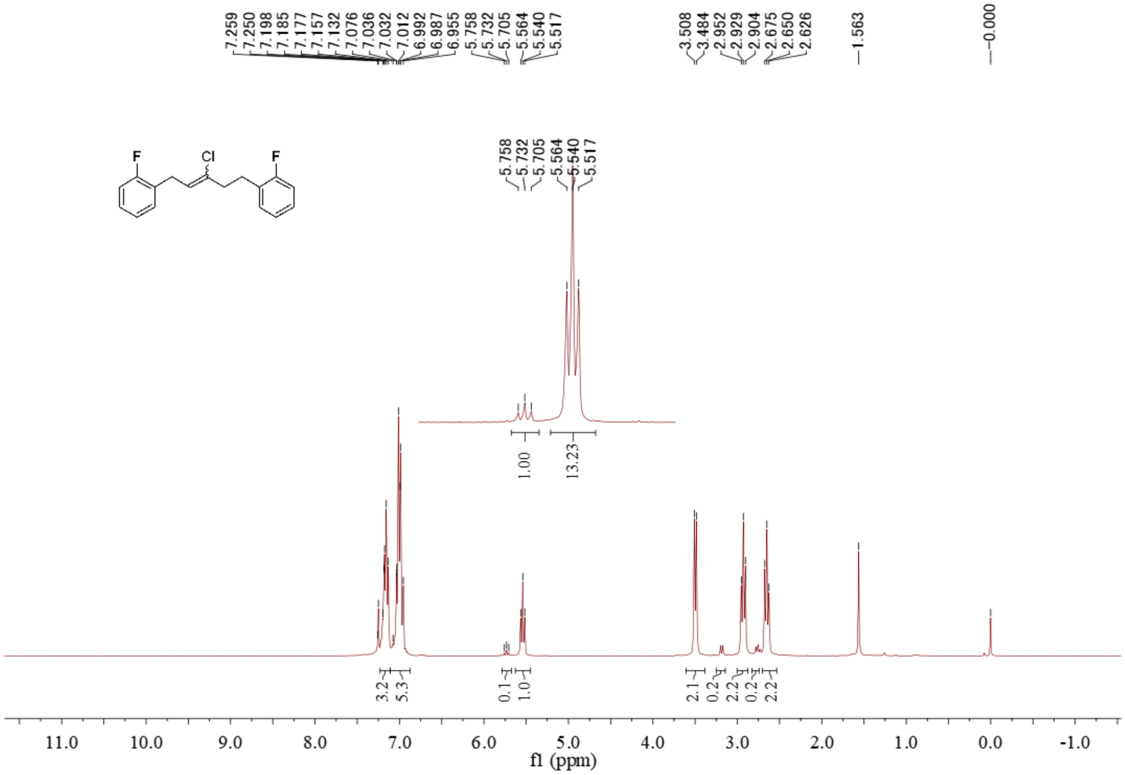

Supplementary Figure 30. ^{13}C NMR spectrum of unknown compounds **3k**, related to **Figure 3**

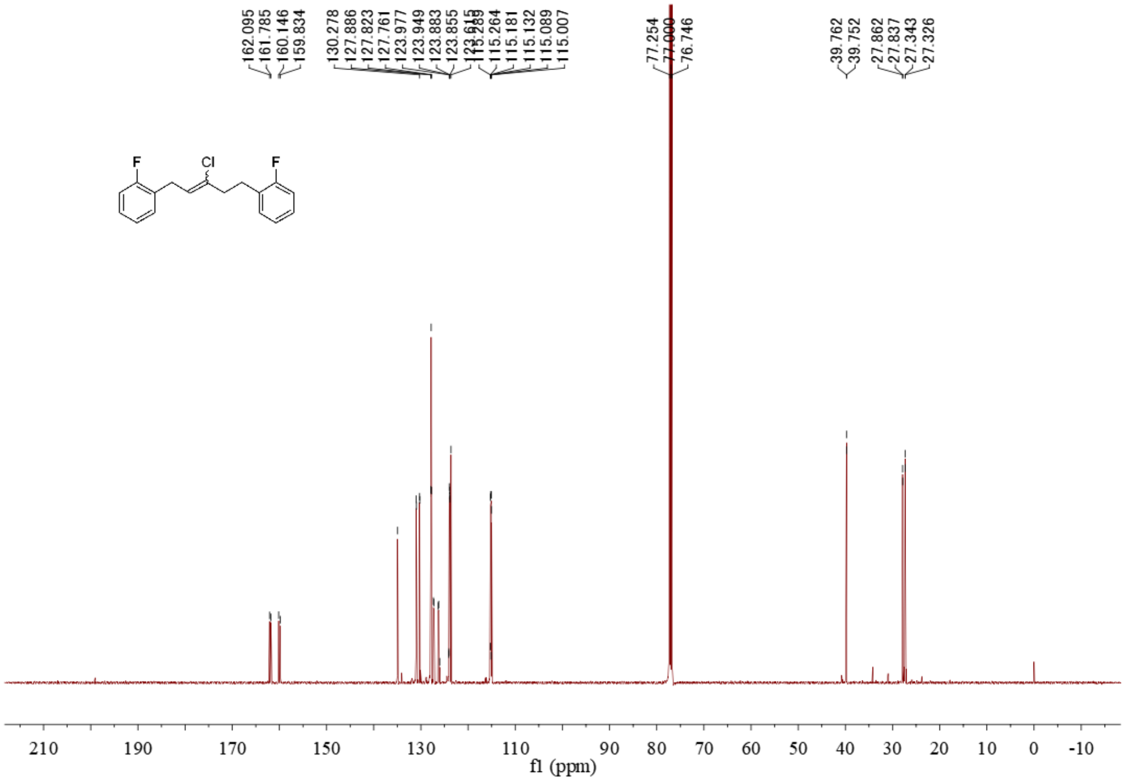

Supplementary Figure 31. ^{1}H NMR spectrum of unknown compounds **3q**, related to **Figure 3**

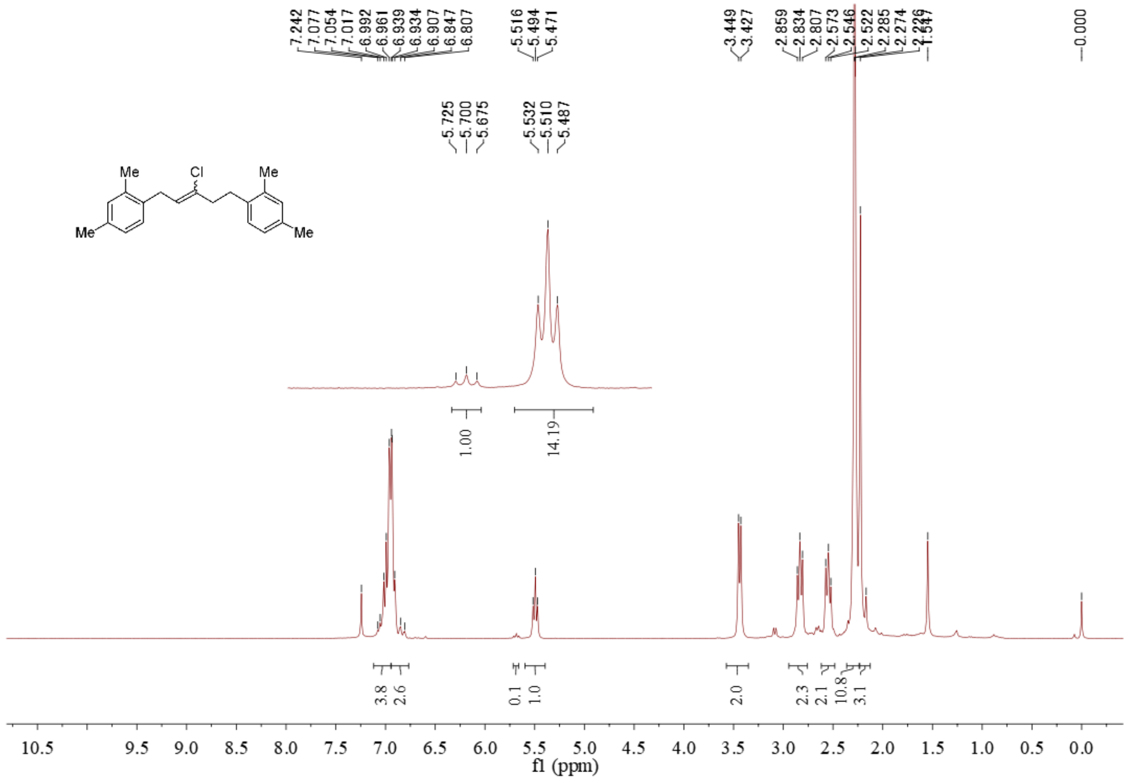

Supplementary Figure 32. ^{13}C NMR spectrum of unknown compounds **3q**, related to **Figure 3**

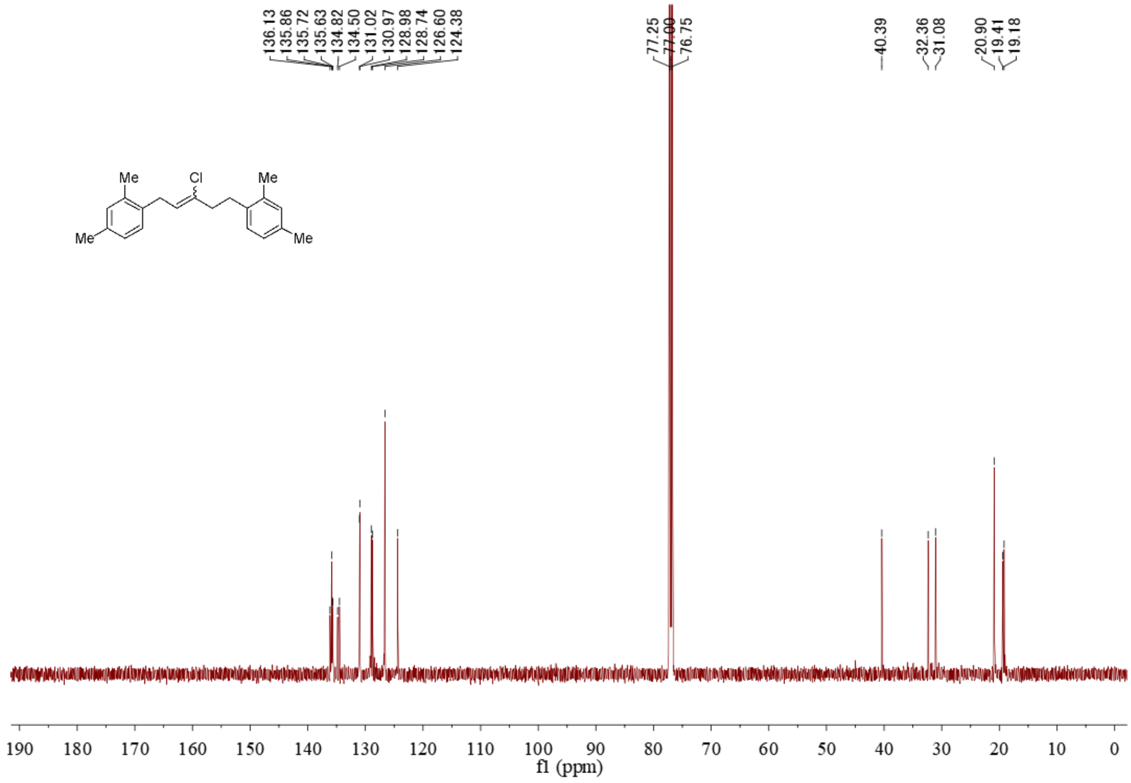

Supplementary Figure 33. ^{1}H NMR spectrum of unknown compounds **3u**, related to **Figure 3**

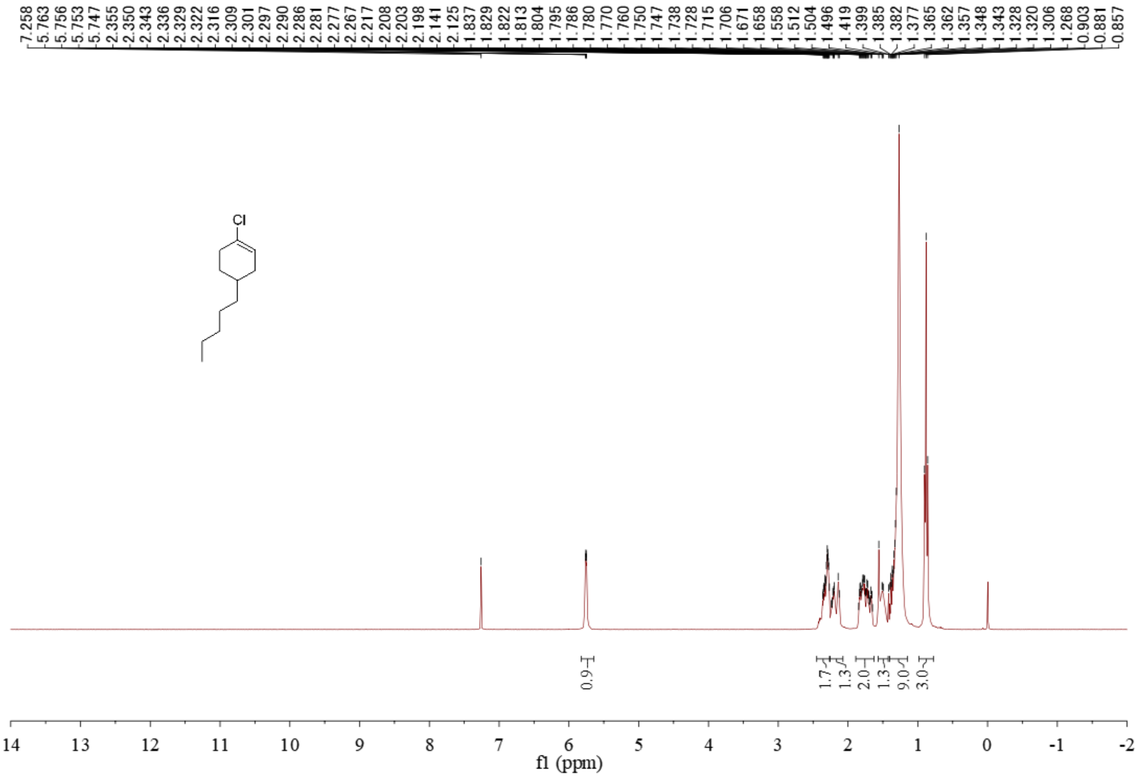

Supplementary Figure 34. ^{13}C NMR spectrum of unknown compounds **3u**, related to **Figure 3**

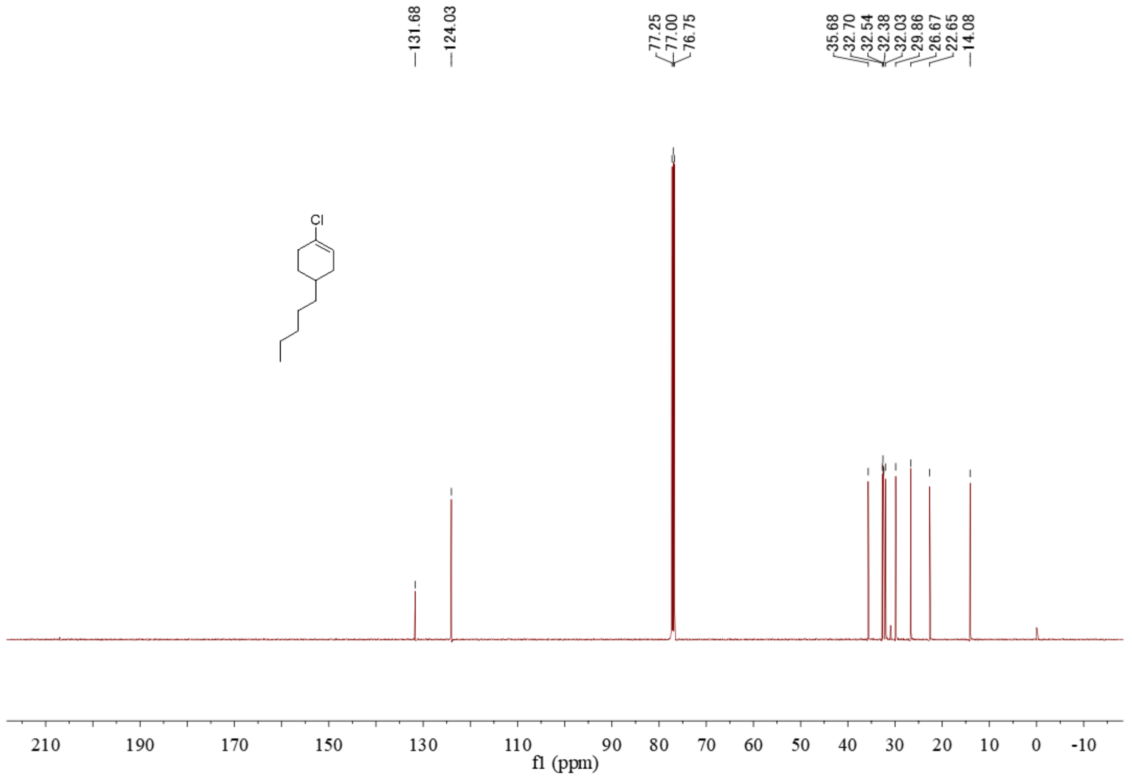

Supplementary Figure 35. ^{1}H NMR spectrum of known compounds **3a**, related to **Figure 3**

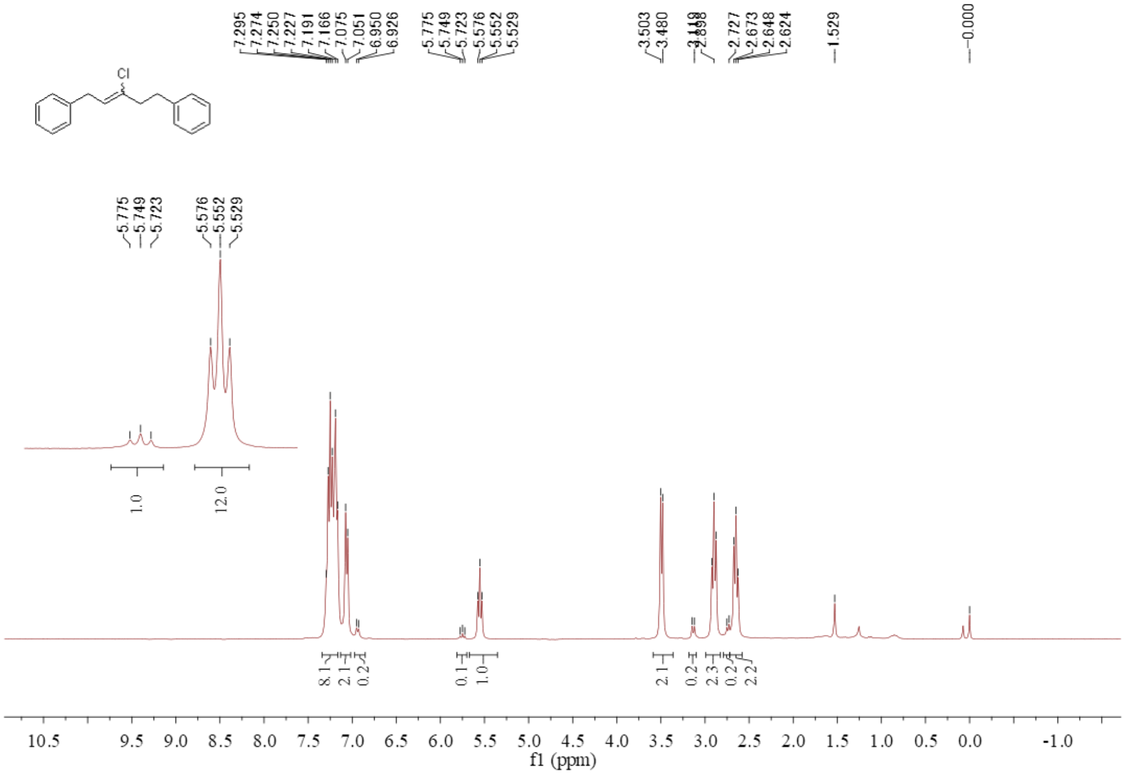

Supplementary Figure 36. ^{13}C NMR spectrum of known compounds **3a**, related to **Figure 3**

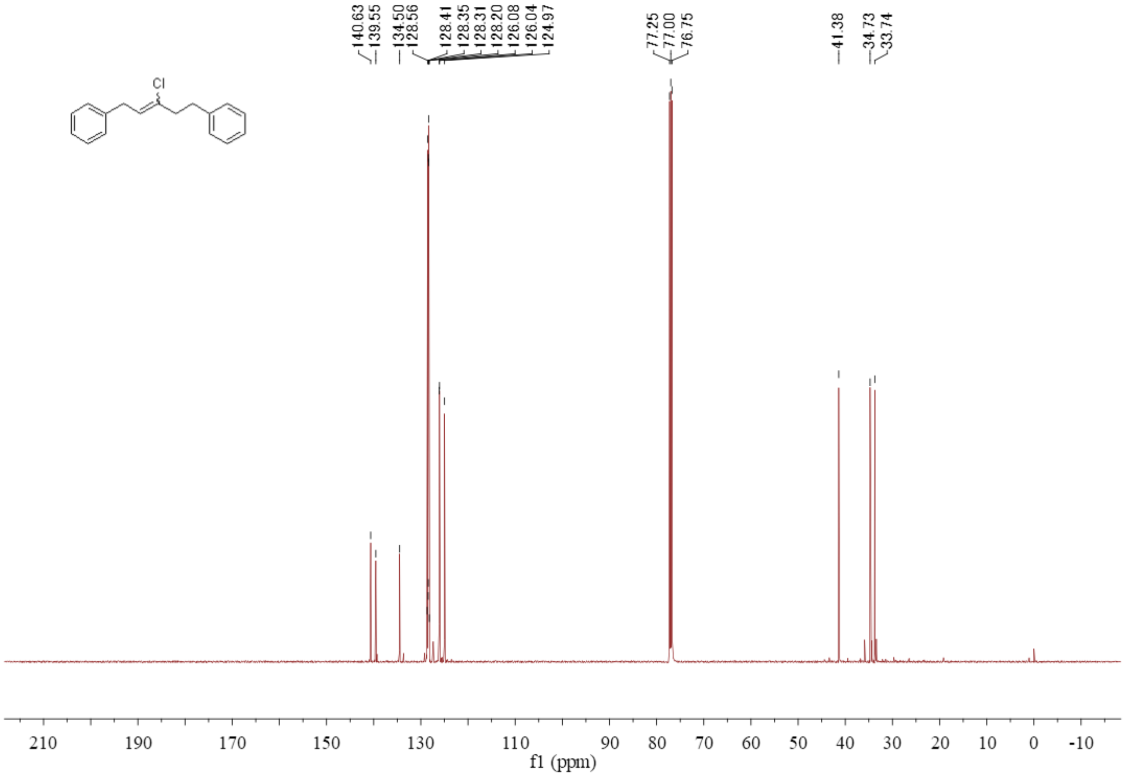

Supplementary Figure 37. ^{1}H NMR spectrum of known compounds **(*Z*)-3r**, related to **Figure 3**

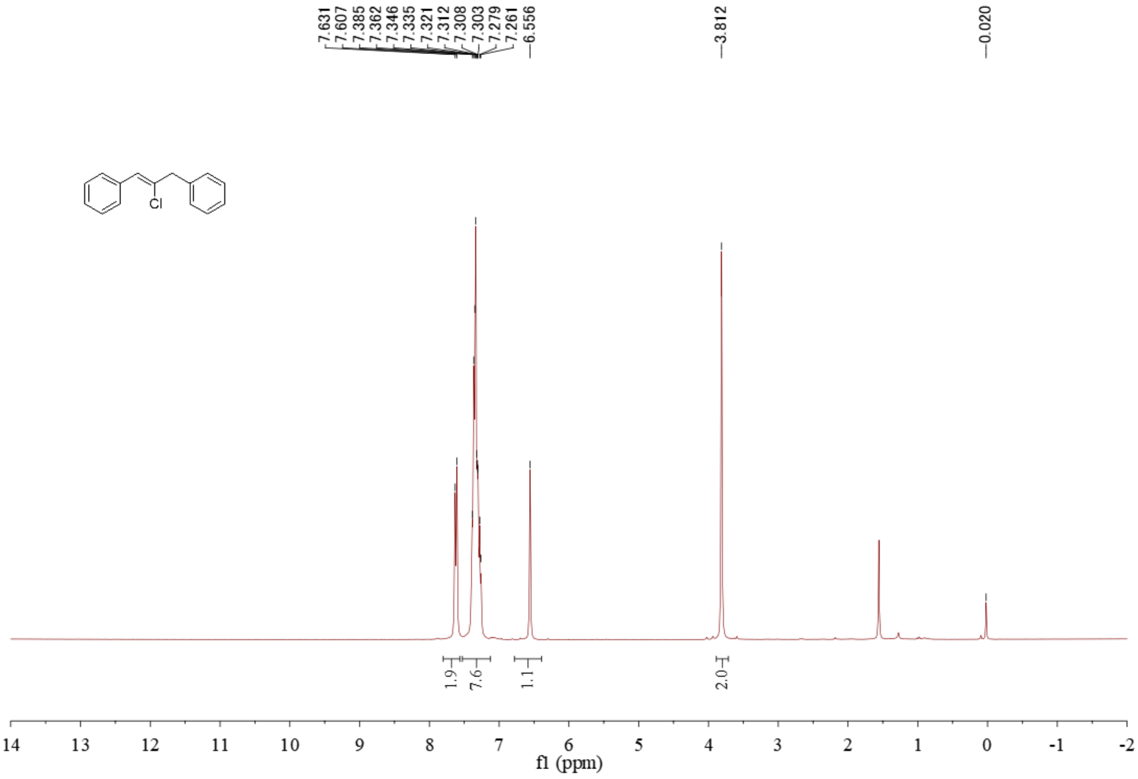

Supplementary Figure 38. ^{13}C NMR spectrum of known compounds **(*Z*)-3r**, related to **Figure 3**

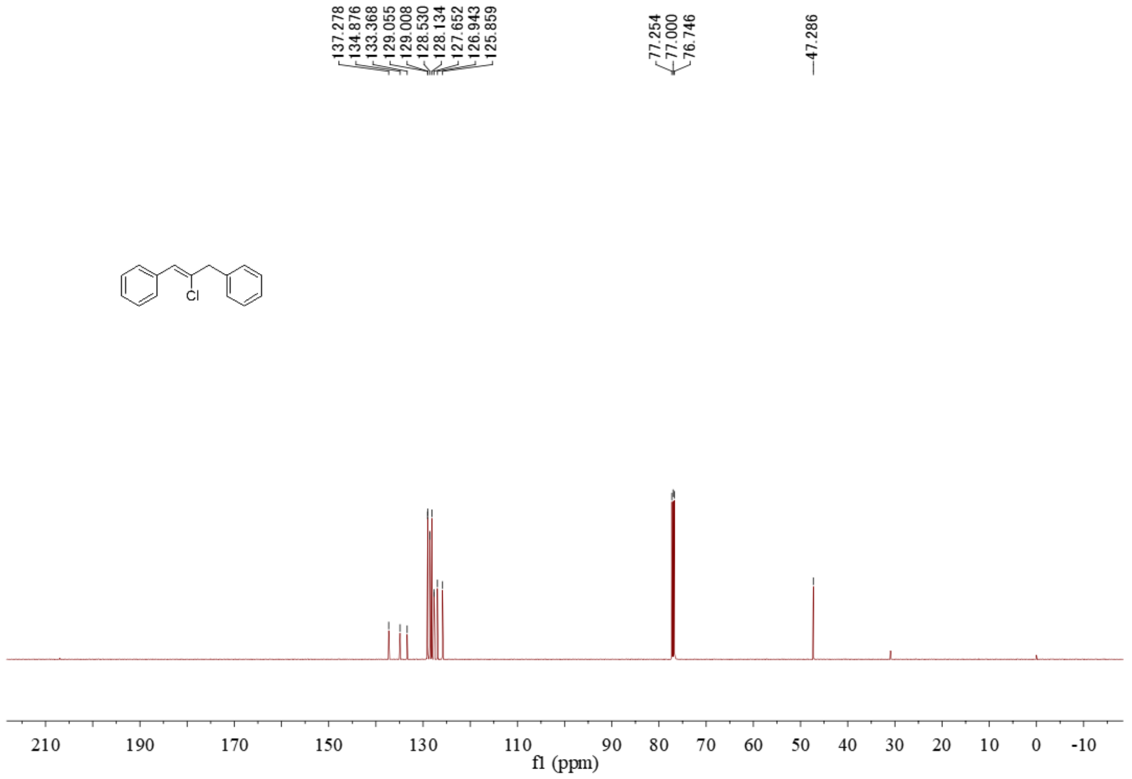

Supplementary Figure 39. ^{1}H NMR spectrum of known compounds **(*E*)-3r**, related to **Figure 3**

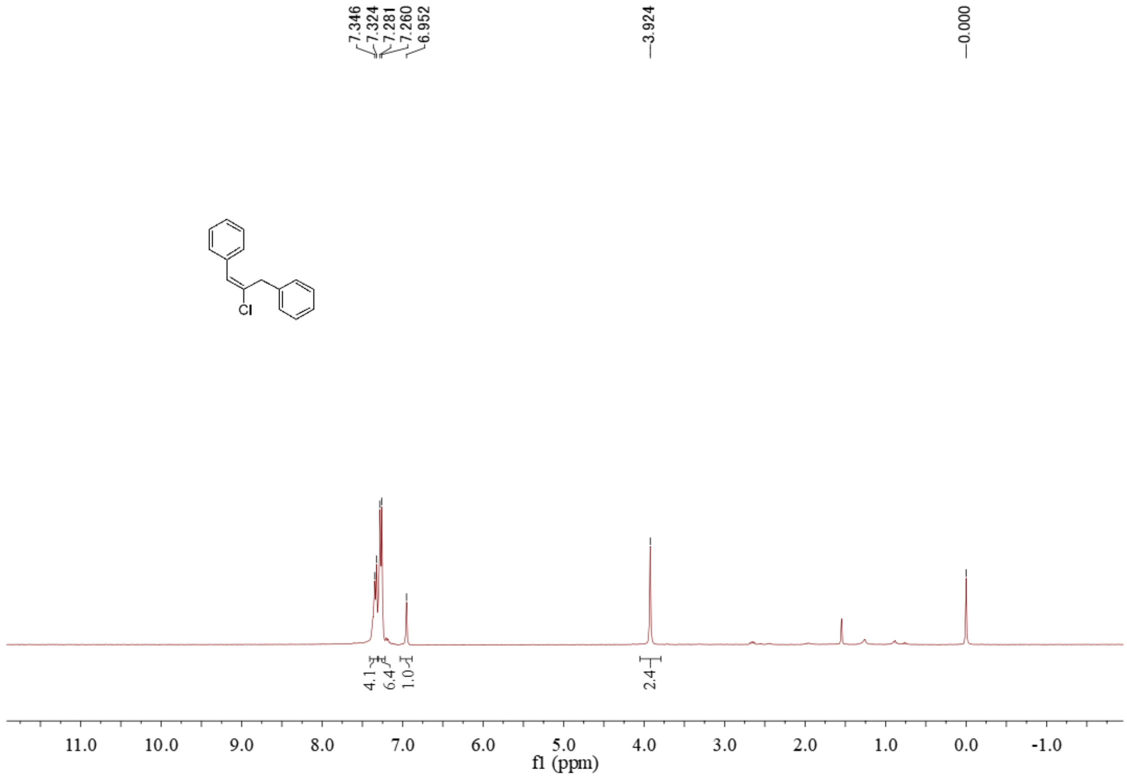

Supplementary Figure 40. ^{13}C NMR spectrum of known compounds **(*E*)-3r**, related to **Figure 3**

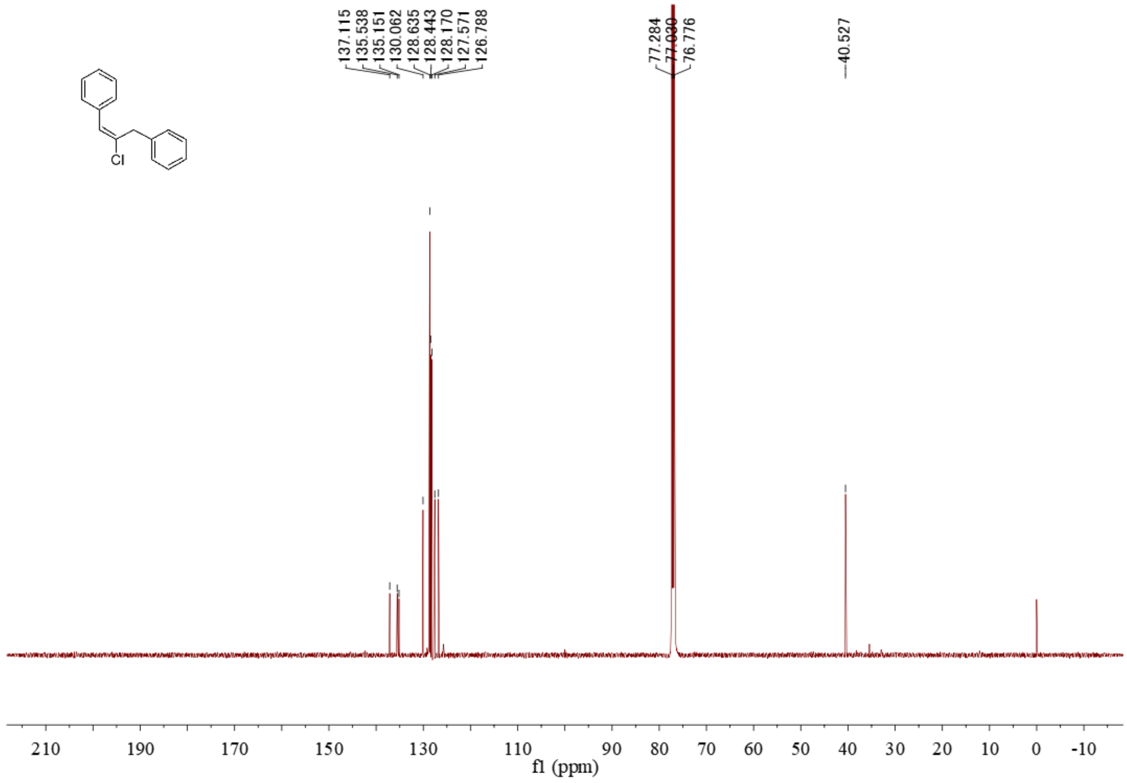

Supplementary Figure 41. ^{1}H NMR spectrum of known compounds **3s**, related to **Figure 3**

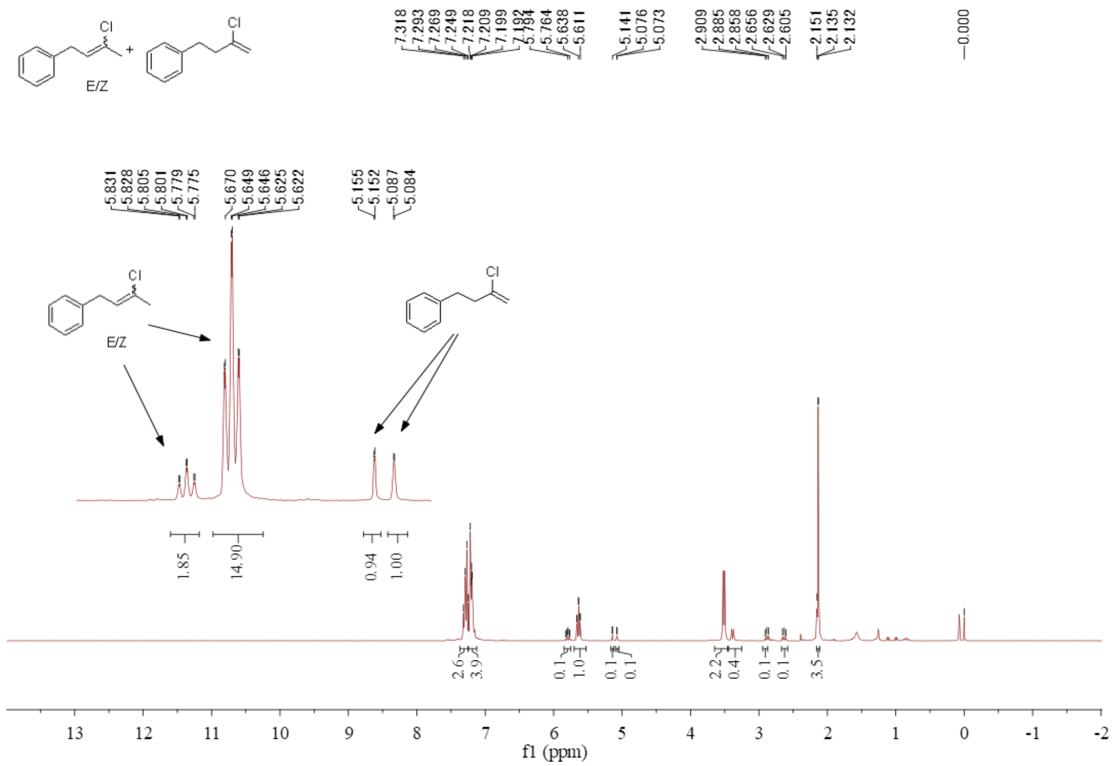

Supplementary Figure 42. ^{13}C NMR spectrum of known compounds **3s**, related to **Figure 3**

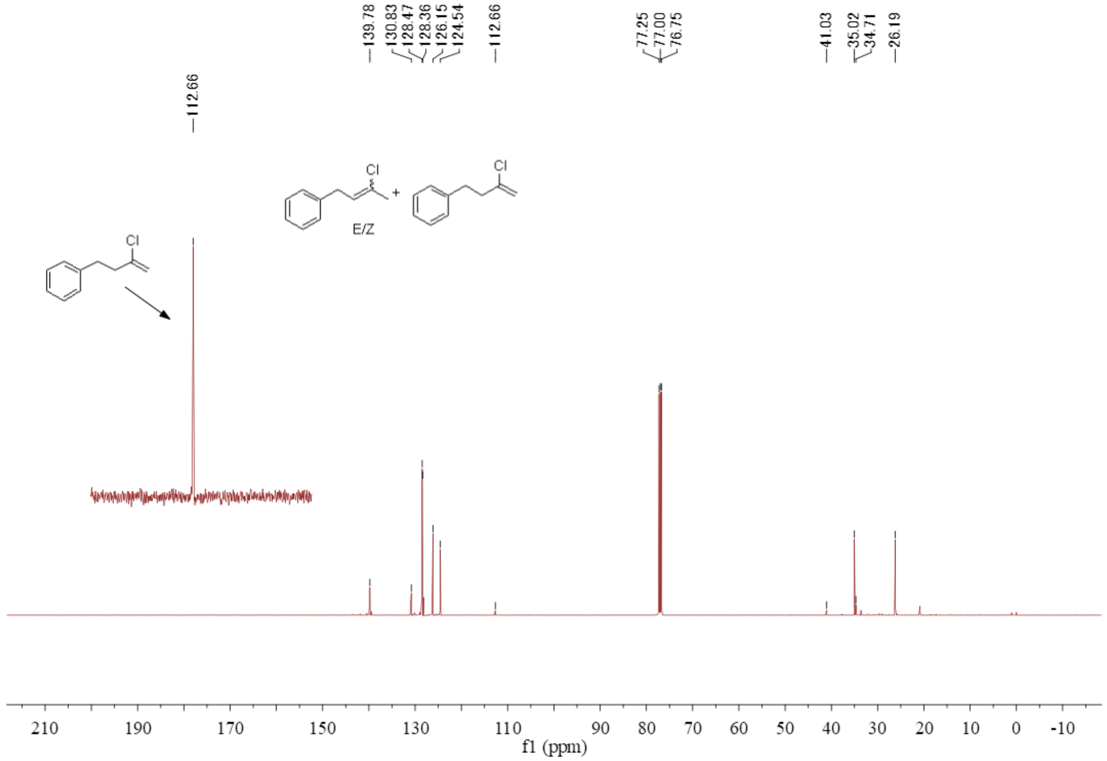

Supplementary Figure 43. ^{1}H NMR spectrum of known compounds **3t**, related to **Figure 3**

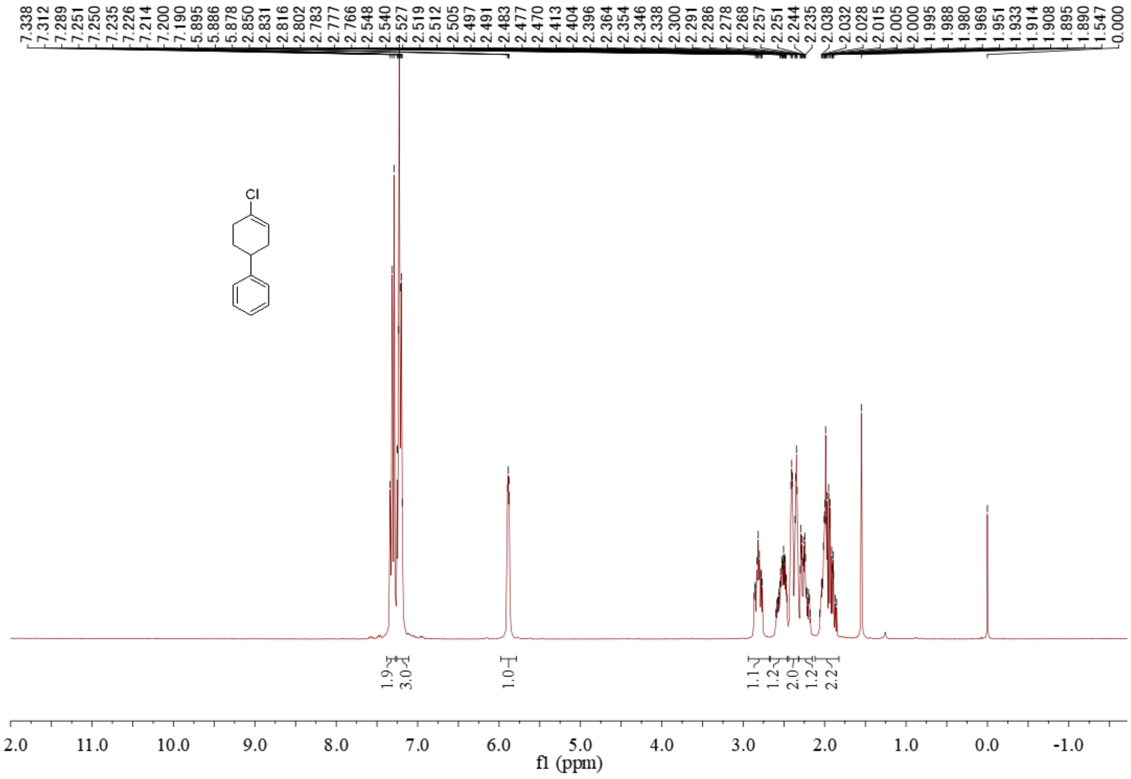

Supplementary Figure 44. ^{13}C NMR spectrum of known compounds **3t**, related to **Figure 3**

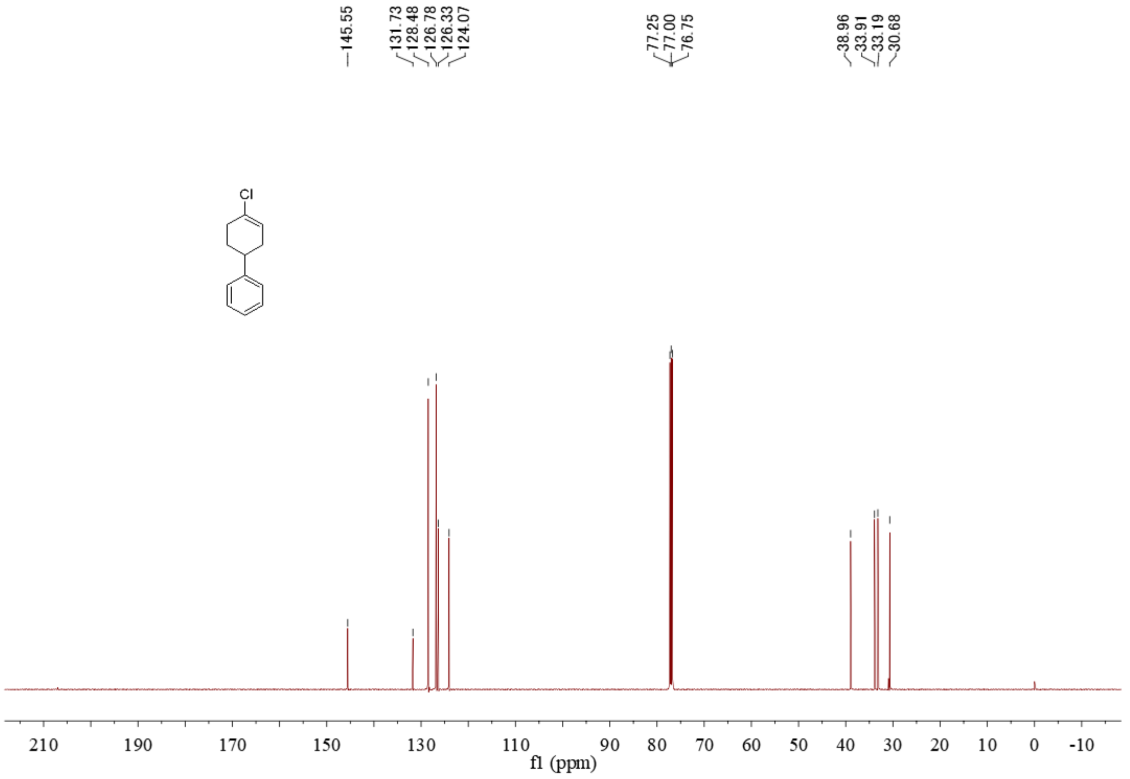

Supplementary Figure 45. ^{1}H NMR spectrum of known compounds **3v**, related to **Figure 3**

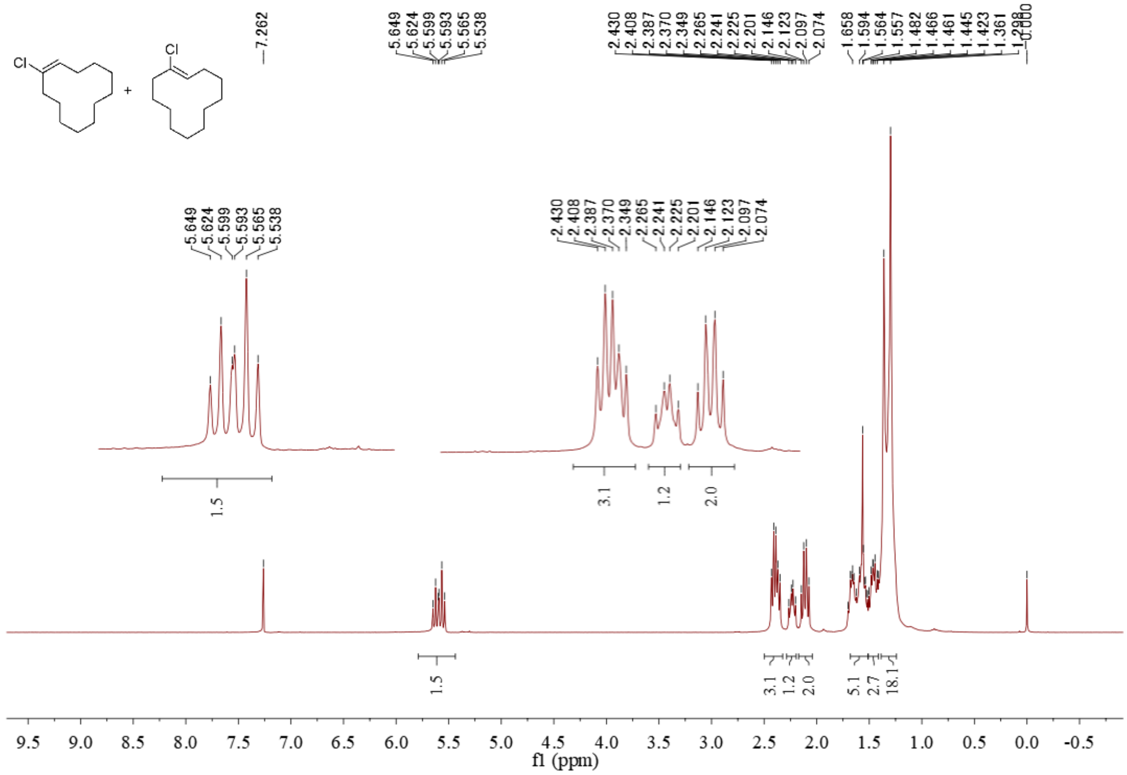

Supplementary Figure 46. ^{13}C NMR spectrum of known compounds **3v**, related to **Figure 3**

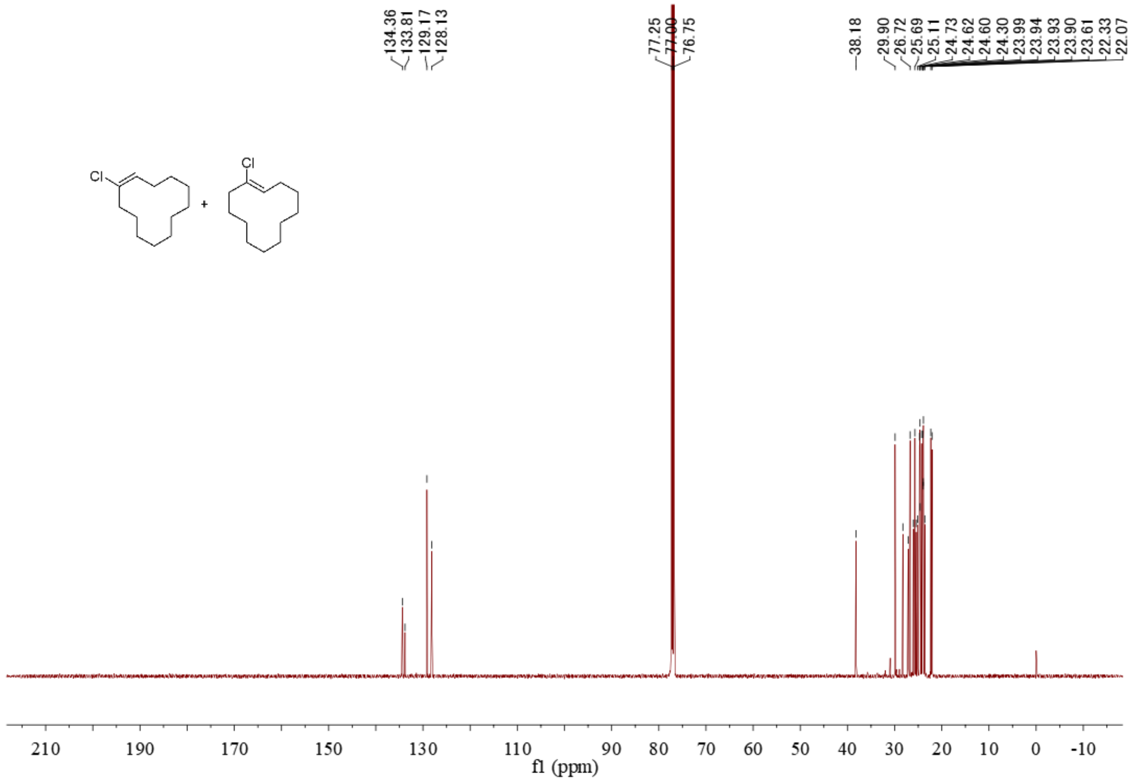

Supplementary Figure 47. ^{1}H NMR spectrum of known compounds **4a**, related to **Figure 4**

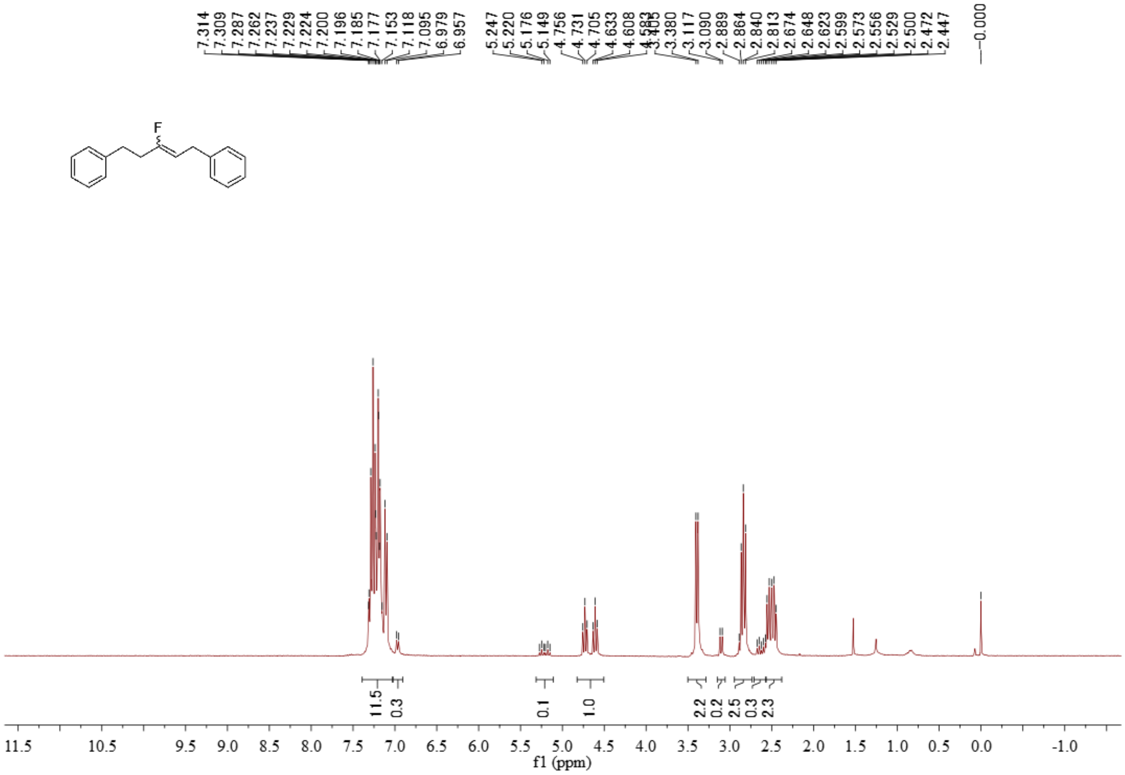

Supplementary Figure 48. ^{19}F NMR spectrum of known compounds **4a**, related to **Figure 4**

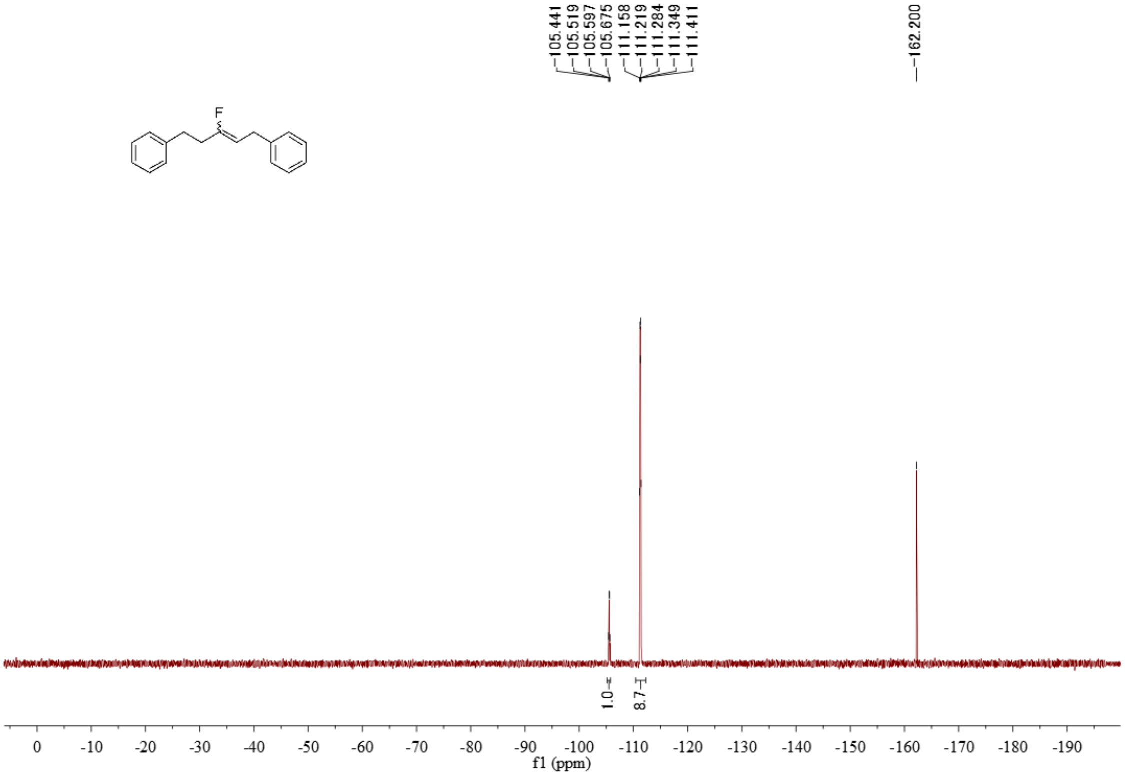

Supplementary Figure 49. ^{1}H NMR spectrum of known compounds **4b**, related to **Figure 4**

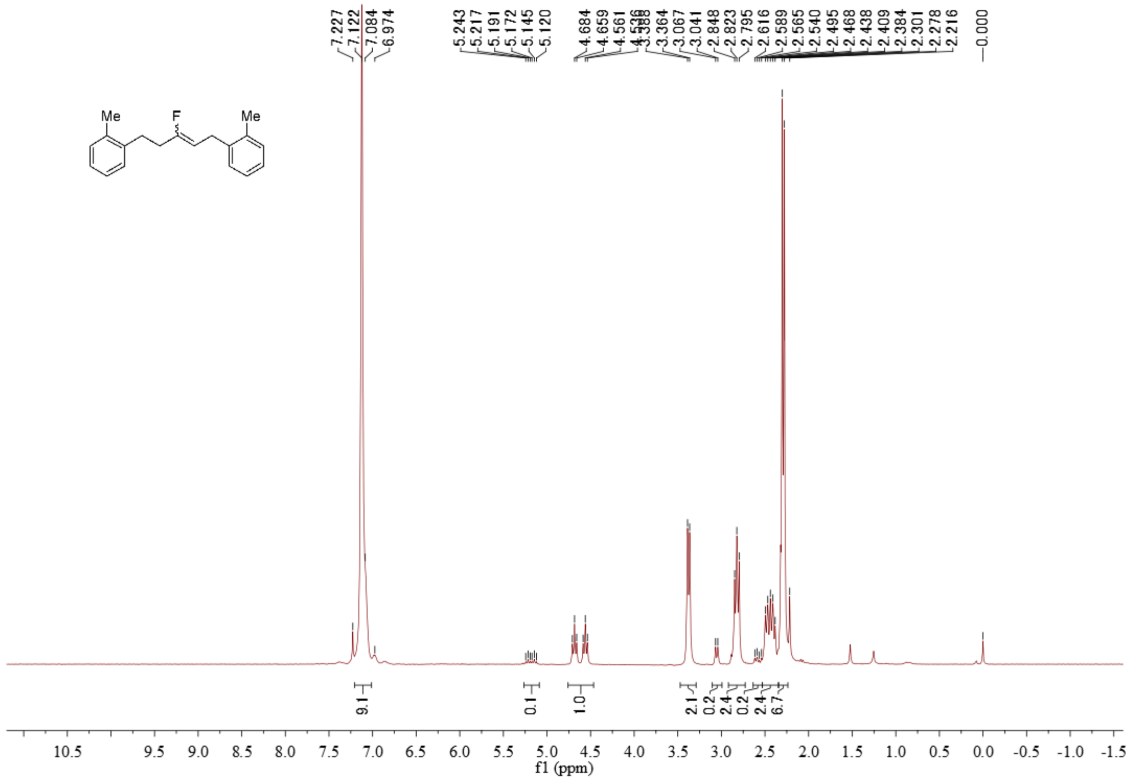

Supplementary Figure 50. ^{19}F NMR spectrum of known compounds **4b**, related to **Figure 4**

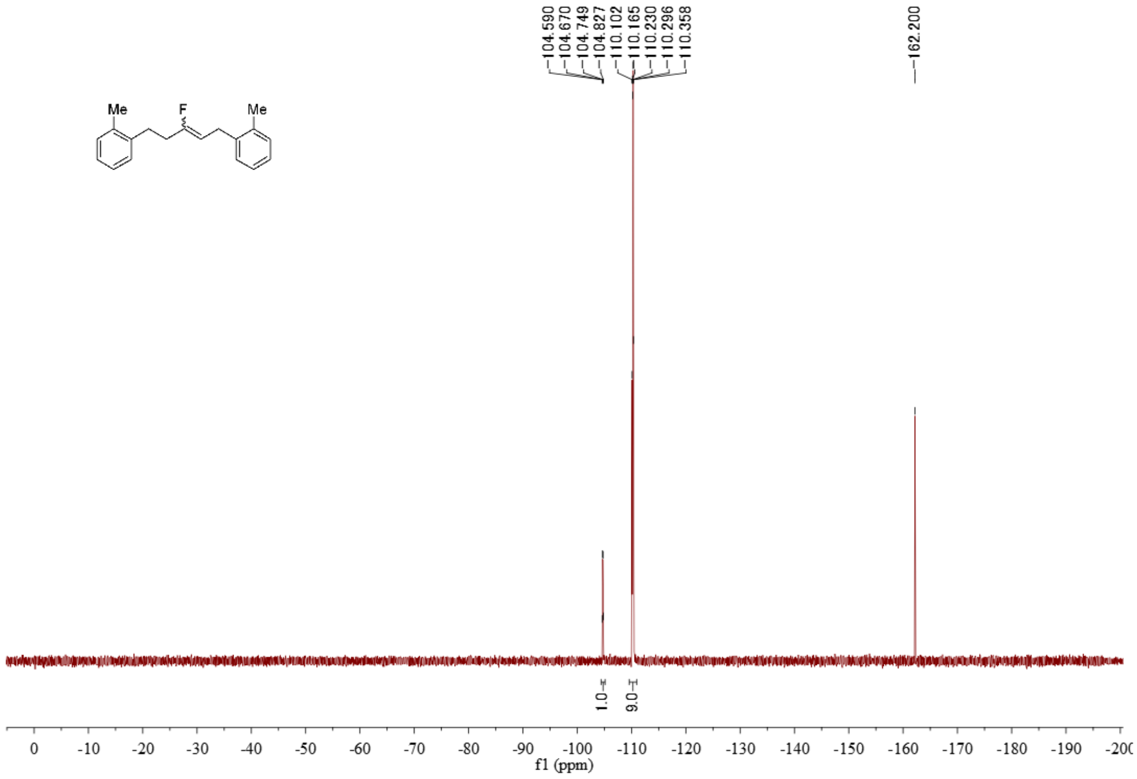

Supplementary Figure 51. ^{1}H NMR spectrum of known compounds **4d**, related to **Figure 4**

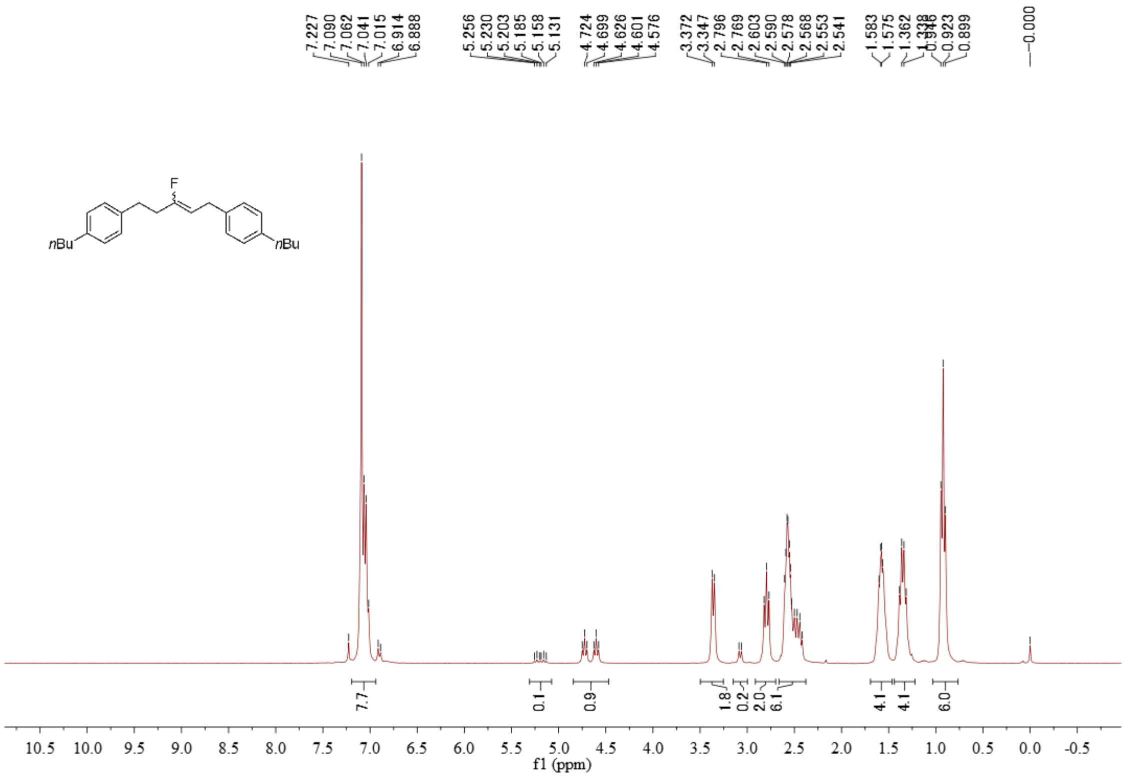

Supplementary Figure 52. ^{19}F NMR spectrum of known compounds **4d**, related to **Figure 4**

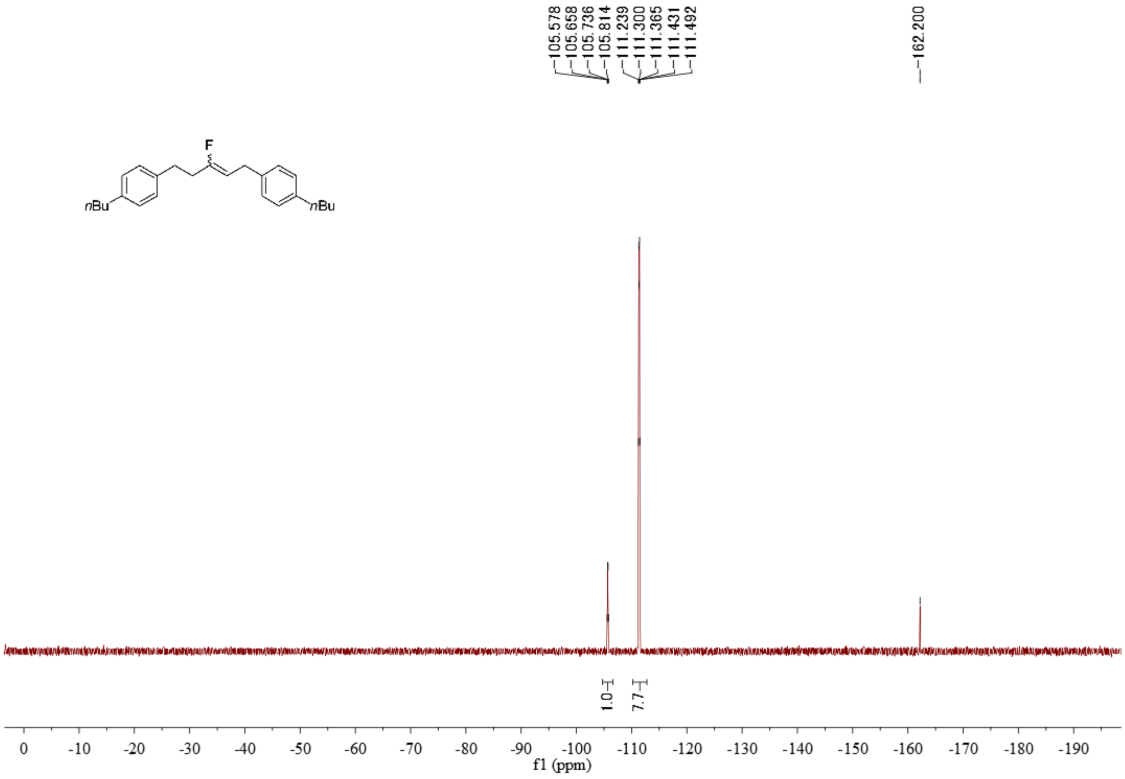

Supplementary Figure 53. ^{1}H NMR spectrum of known compounds **(*Z*)-4i**, related to **Figure 4**

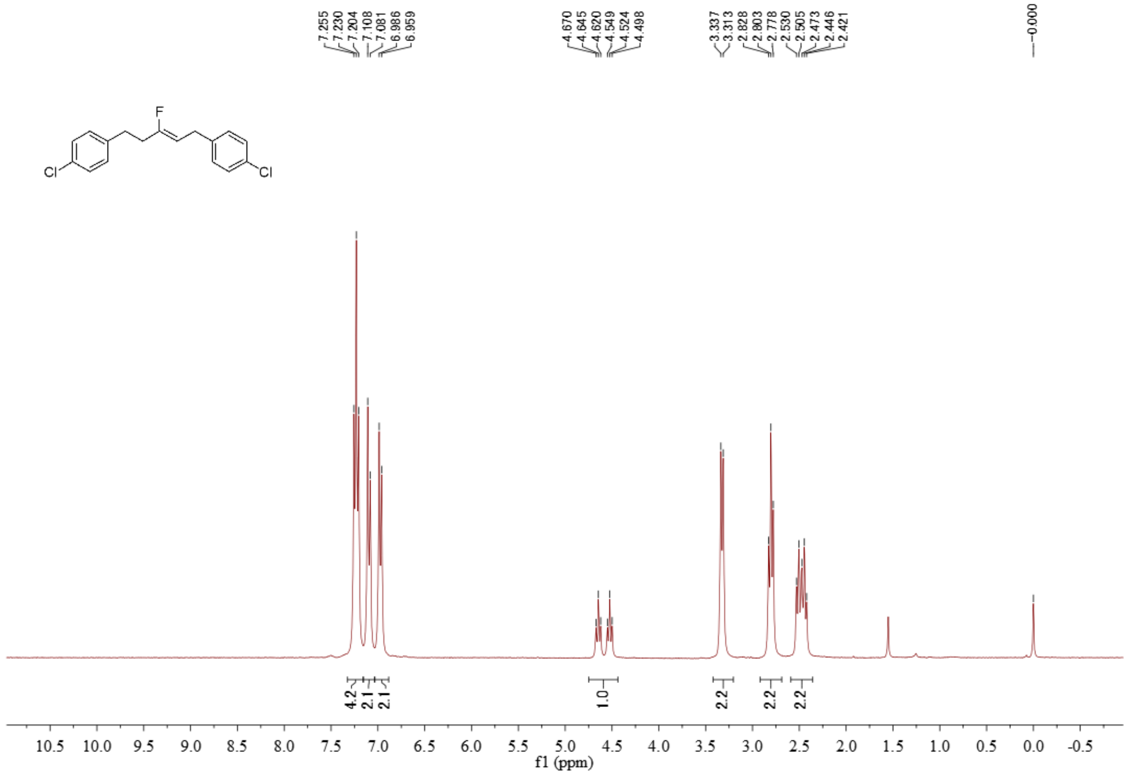

Supplementary Figure 54. ^{19}F NMR spectrum of known compounds **(*Z*)-4i**, related to **Figure 4**

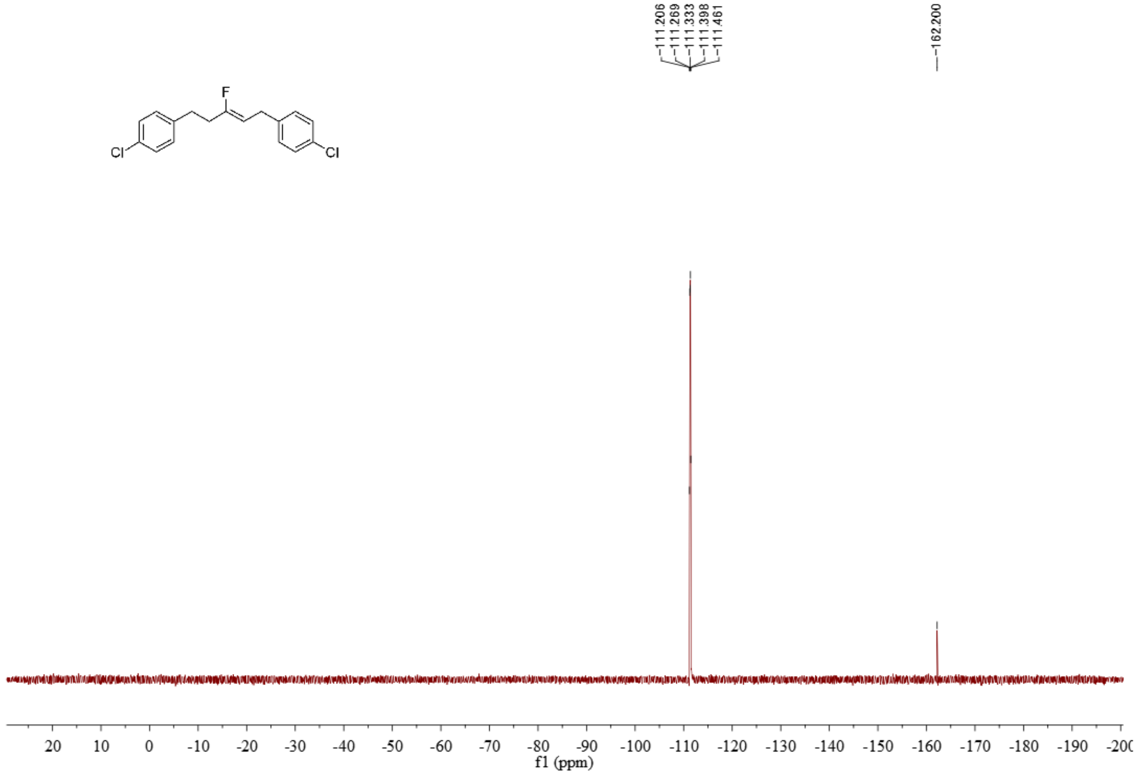

Supplementary Figure 55. ^{1}H NMR spectrum of known compounds **(*E*)-4i**, related to **Figure 4**

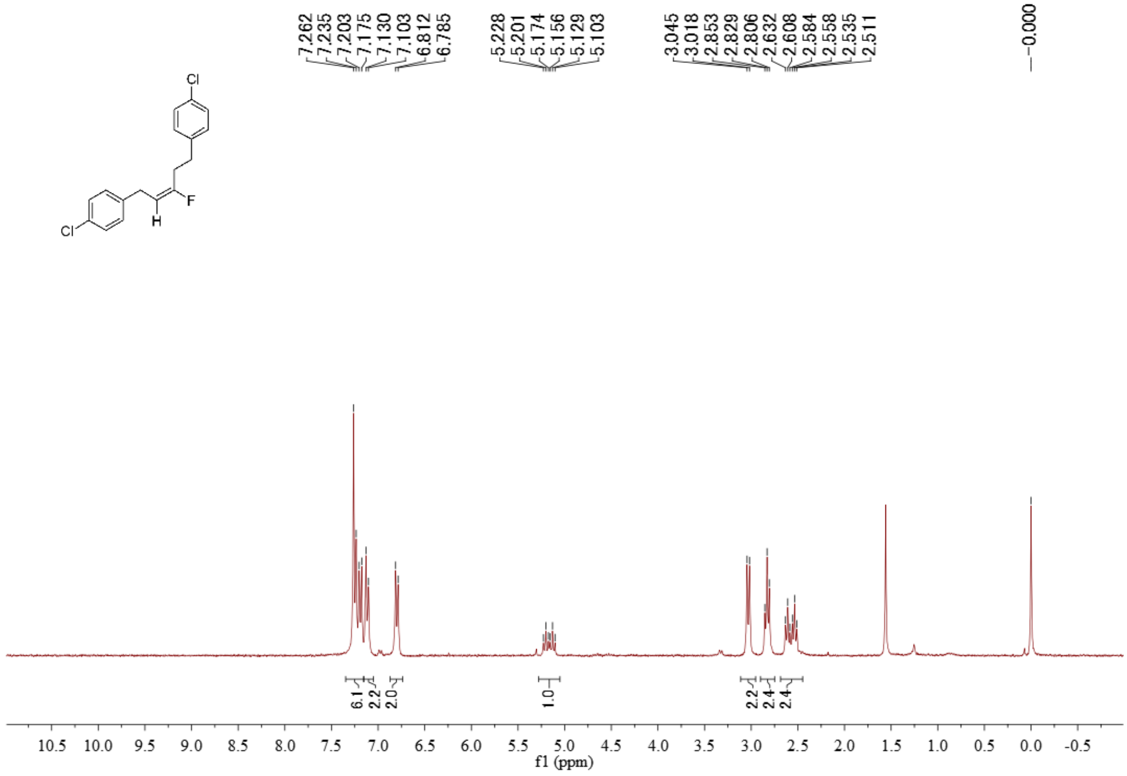

Supplementary Figure 56. ^{19}F NMR spectrum of known compounds **(*E*)-4i**, related to **Figure 4**

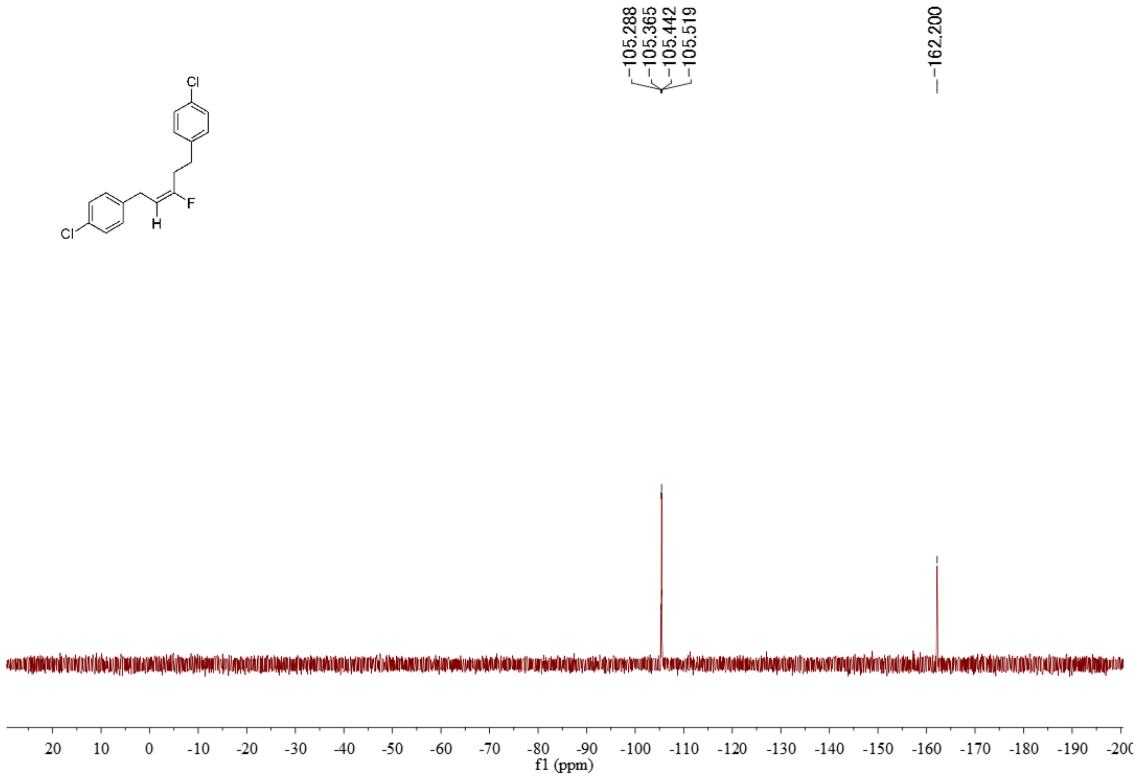

Supplementary Figure 57. ^{1}H NMR spectrum of known compounds **(*Z*)-4j**, related to **Figure 4**

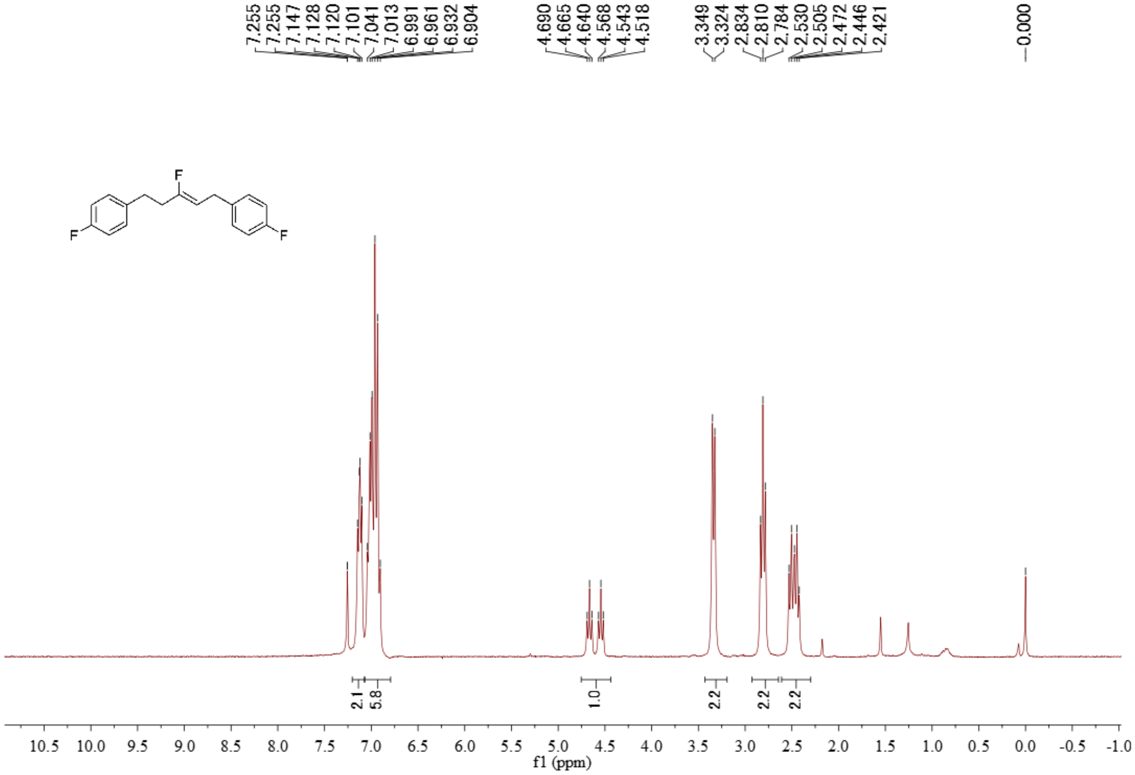

Supplementary Figure 58. ^{19}F NMR spectrum of known compounds **(*Z*)-4j**, related to **Figure 4**

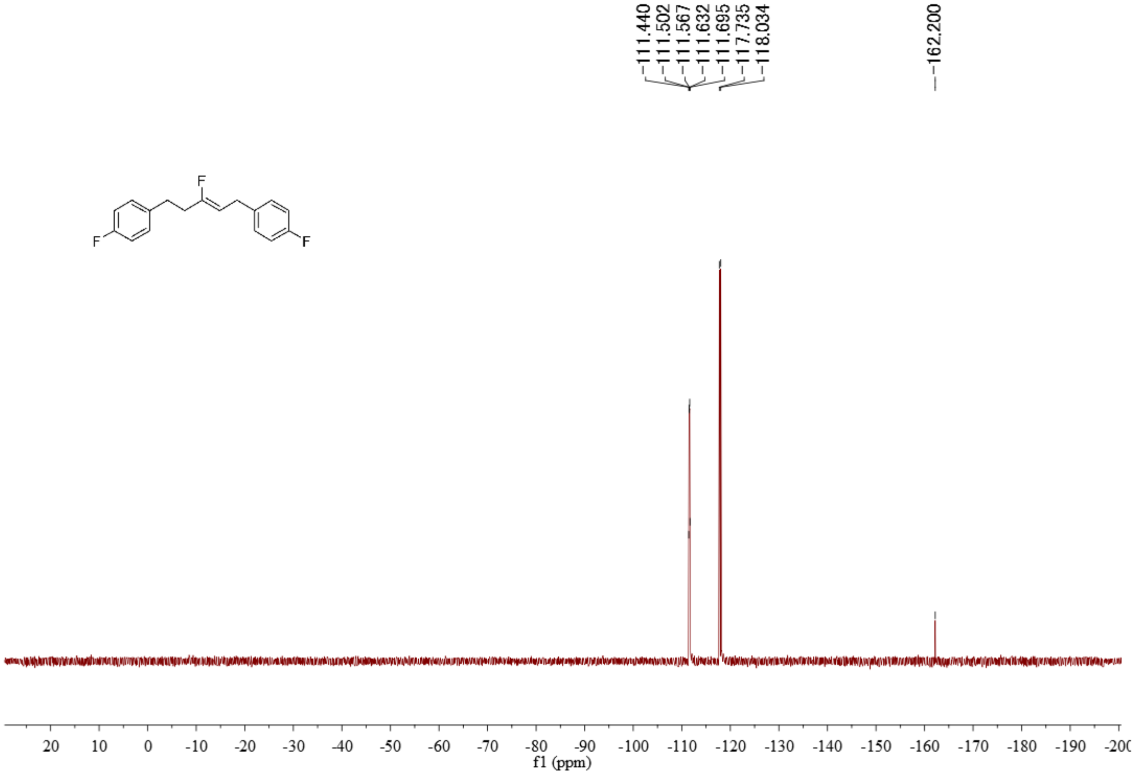

Supplementary Figure 59. ^{1}H NMR spectrum of known compounds ***(E)*-4j**, related to **Figure 4**

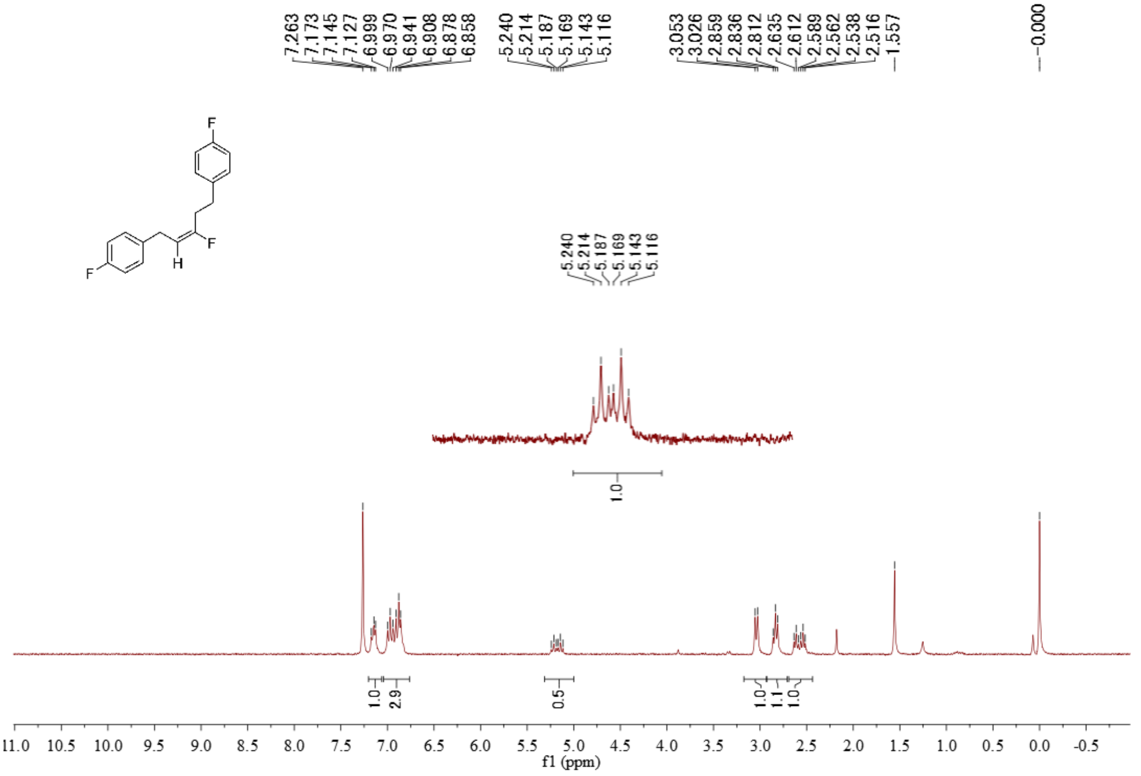

Supplementary Figure 60. ^{19}F NMR spectrum of known compounds ***(E)*-4j**, related to **Figure 4**

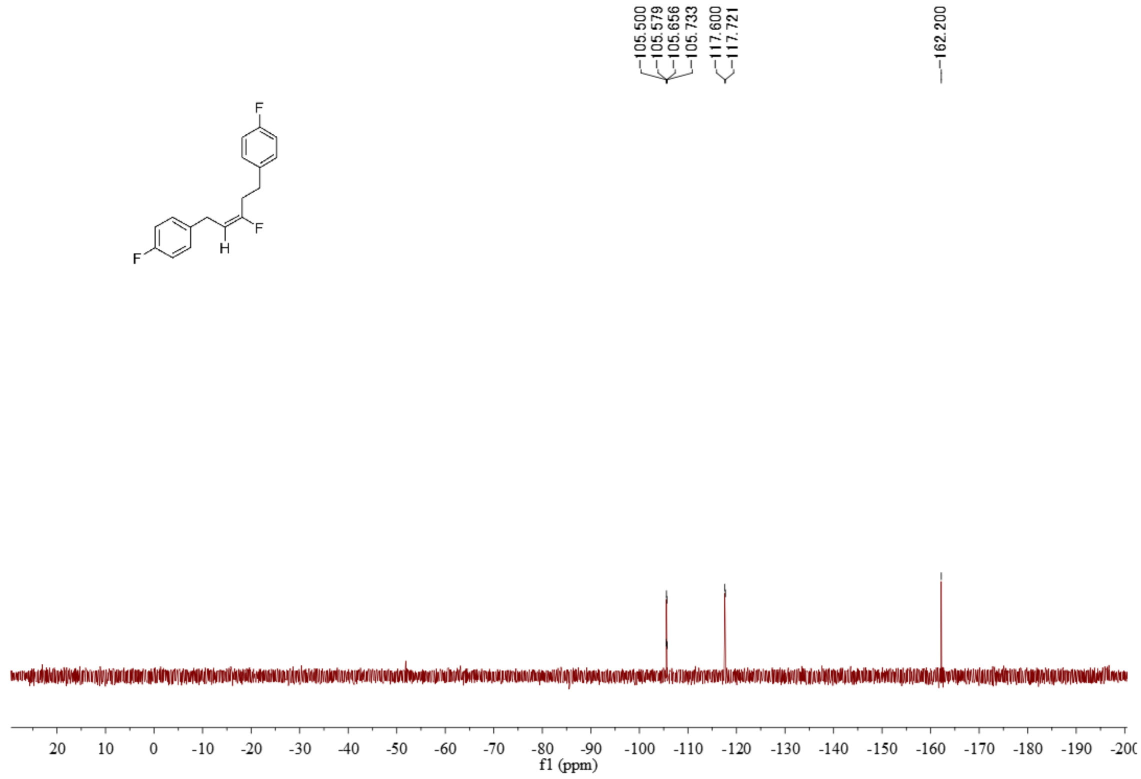

Supplementary Figure 61. ^{1}H NMR spectrum of known compounds **4f**, related to **Figure 4**

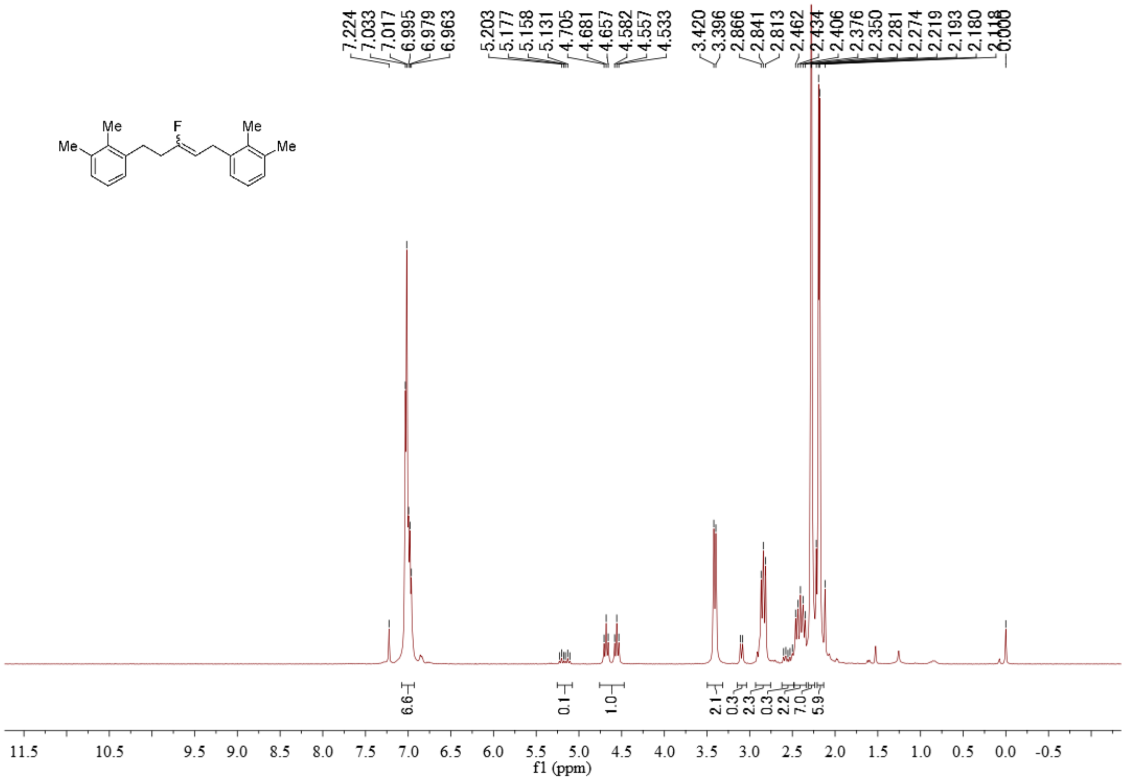

Supplementary Figure 62. ^{19}F NMR spectrum of known compounds **4f**, related to **Figure 4**

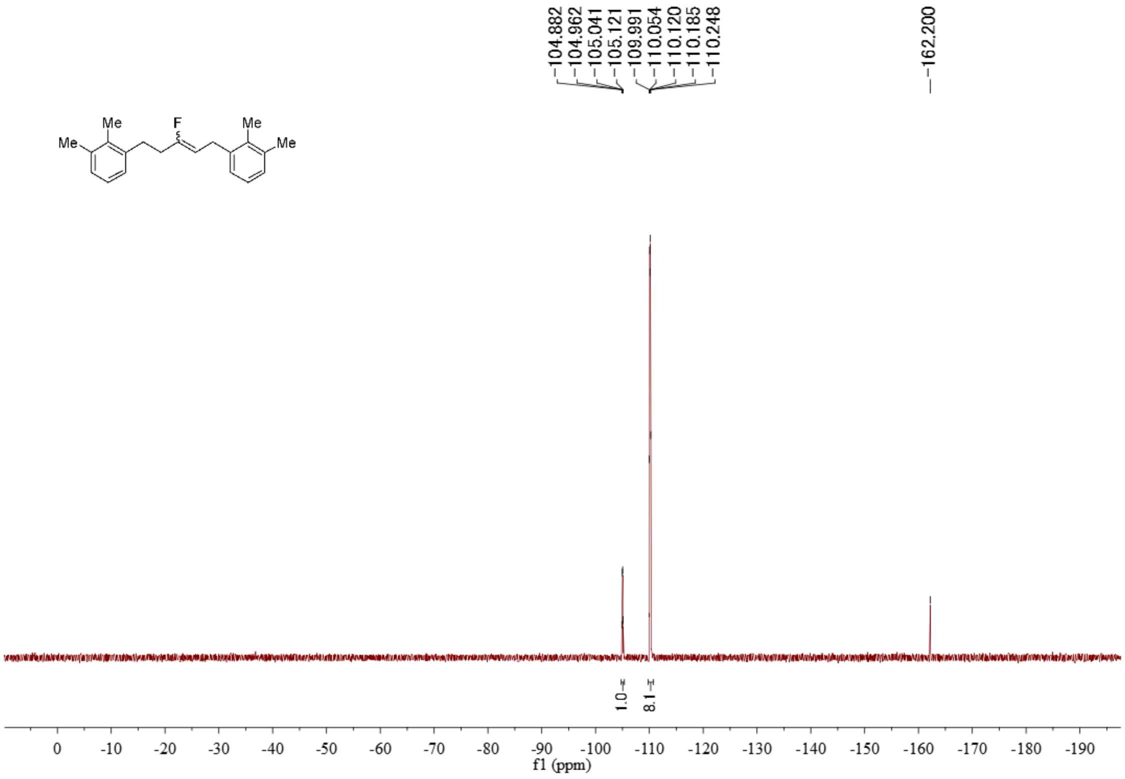

Supplementary Figure 63. ^{1}H NMR spectrum of known compounds **4q**, related to **Figure 4**

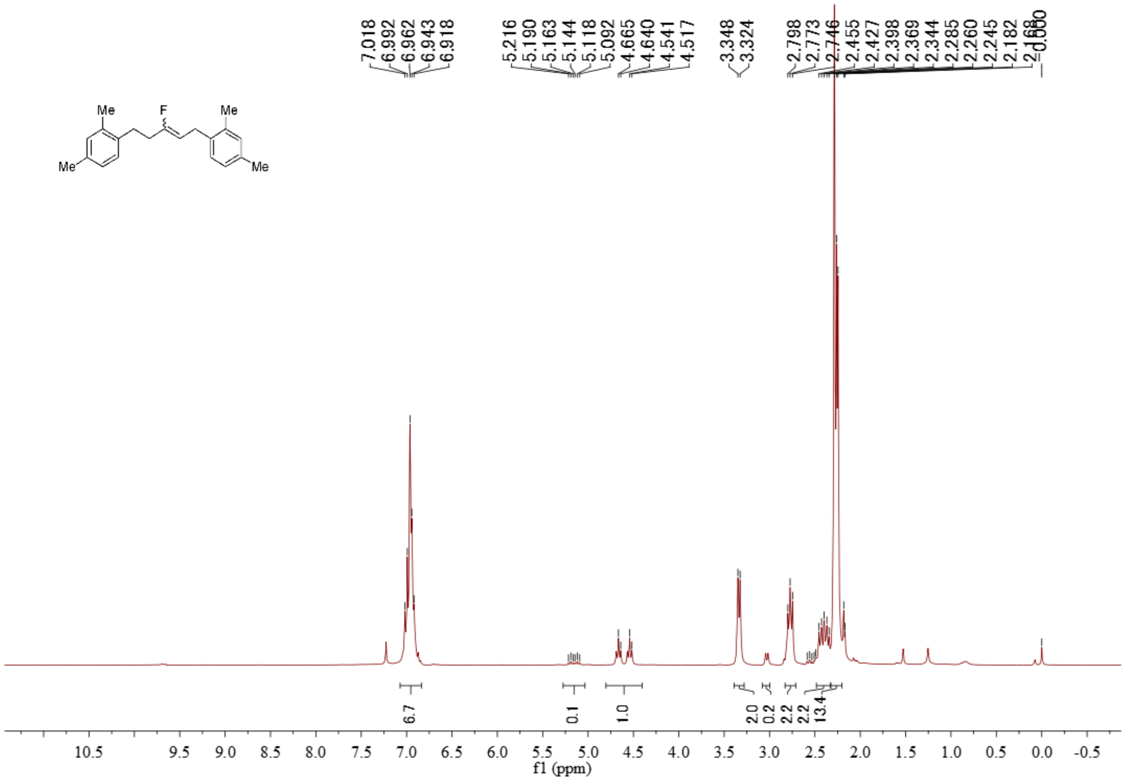

Supplementary Figure 64. ^{19}F NMR spectrum of known compounds **4q**, related to **Figure 4**

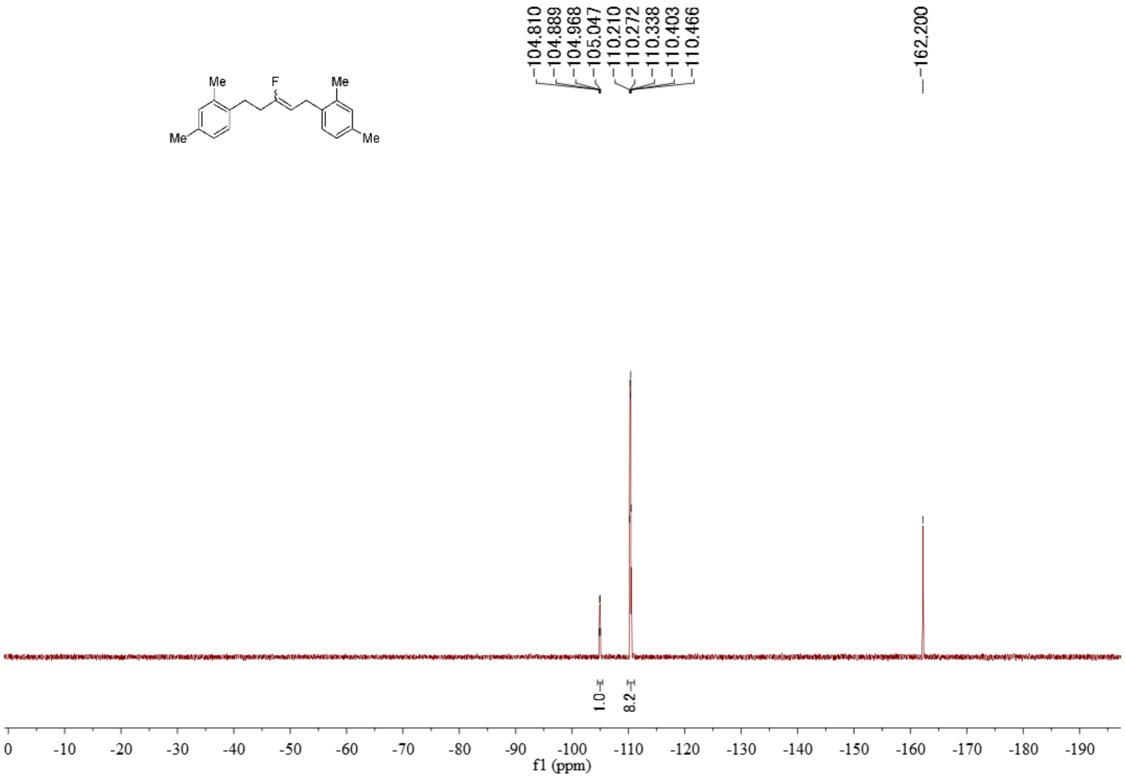

Supplementary Figure 65. ^{1}H NMR spectrum of known compounds **4r**, related to **Figure 4**

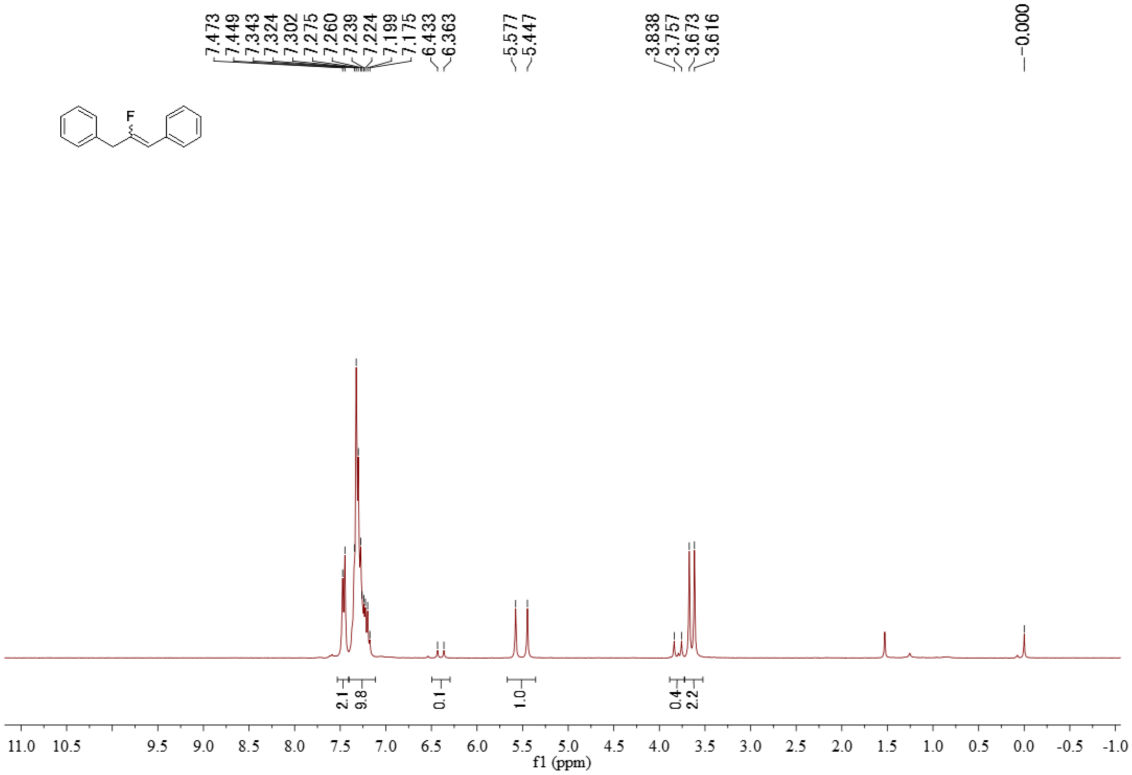

Supplementary Figure 66. ^{19}F NMR spectrum of known compounds **4r**, related to **Figure 4**

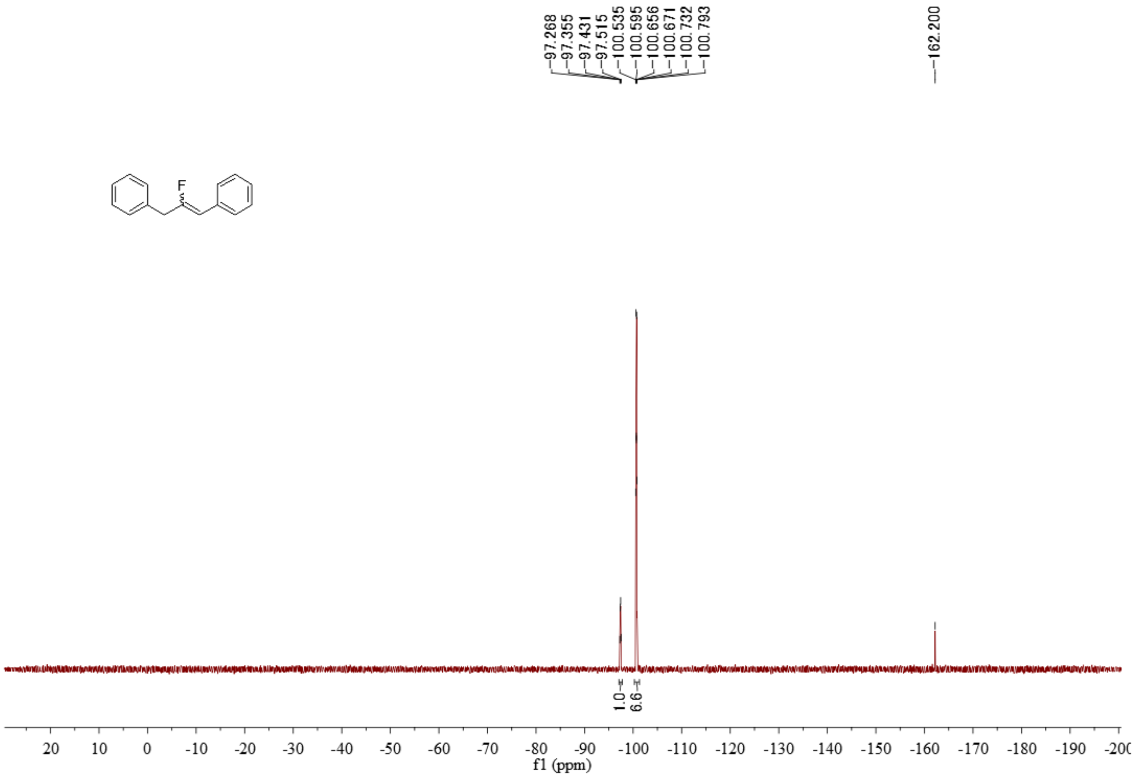

Supplementary Figure 67. ^{1}H NMR spectrum of known compounds **4t**, related to **Figure 4**

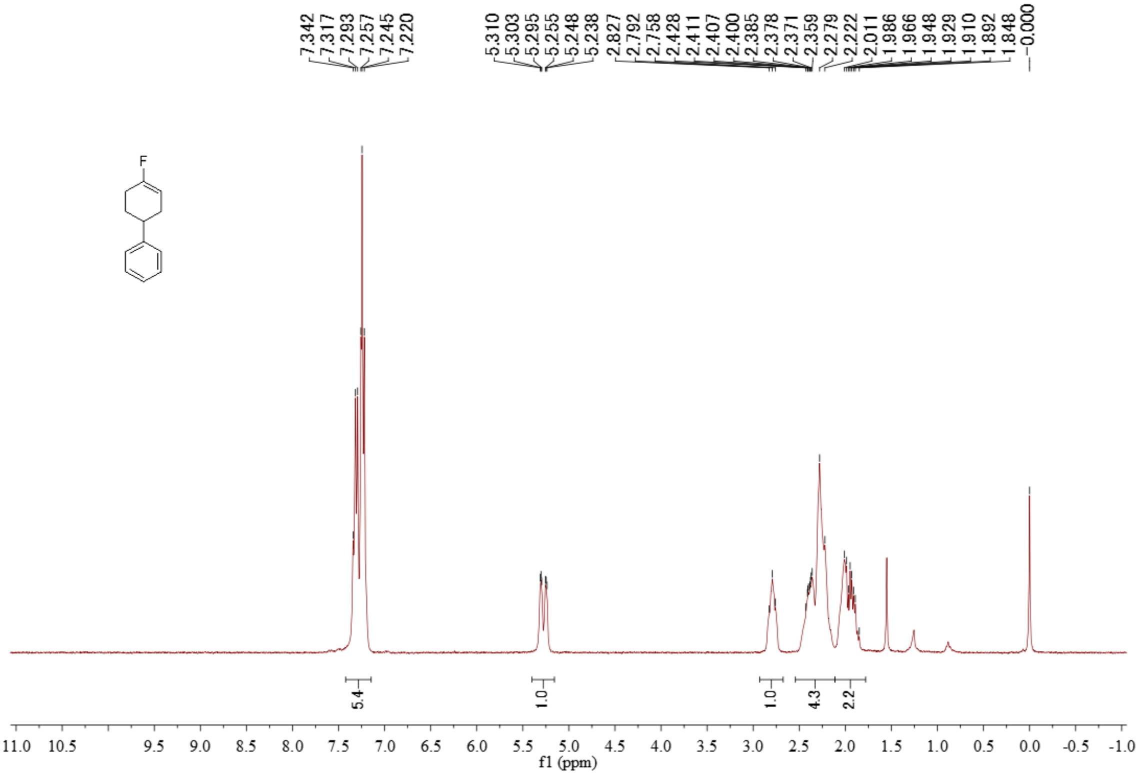

Supplementary Figure 68. ^{19}F NMR spectrum of known compounds **4t**, related to **Figure 4**

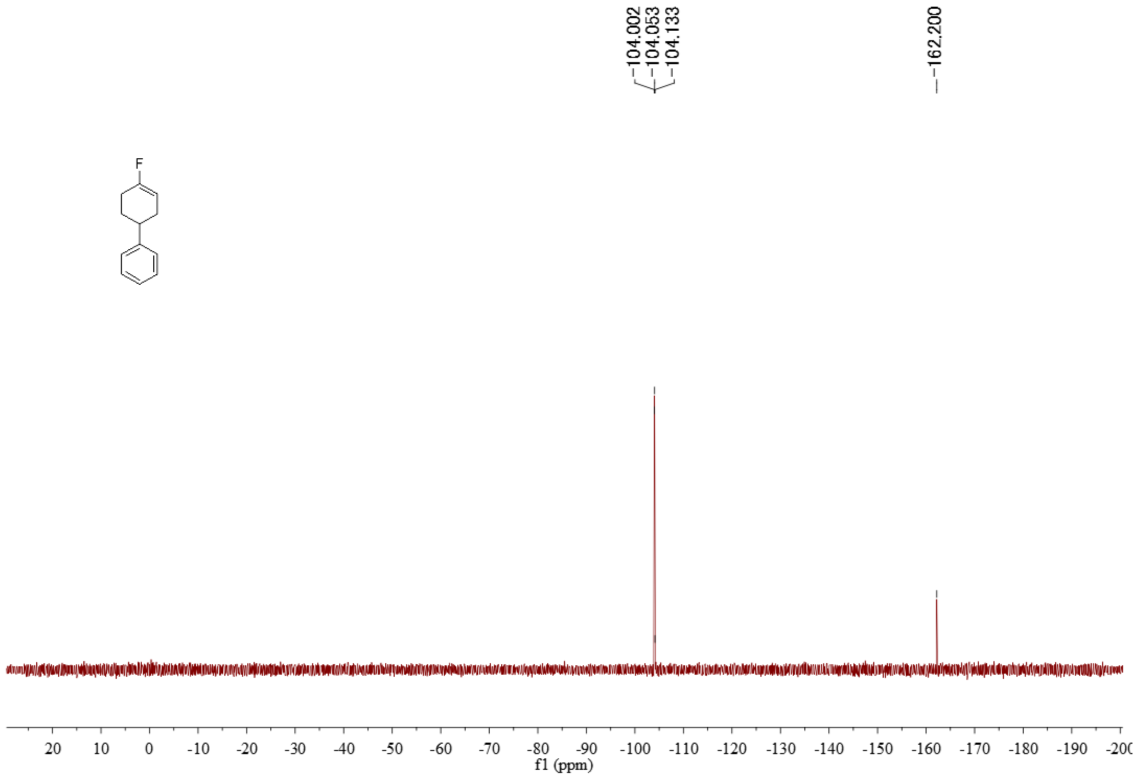

Supplementary Figure 69. ^{1}H NMR spectrum of known compounds **4u**, related to **Figure 4**

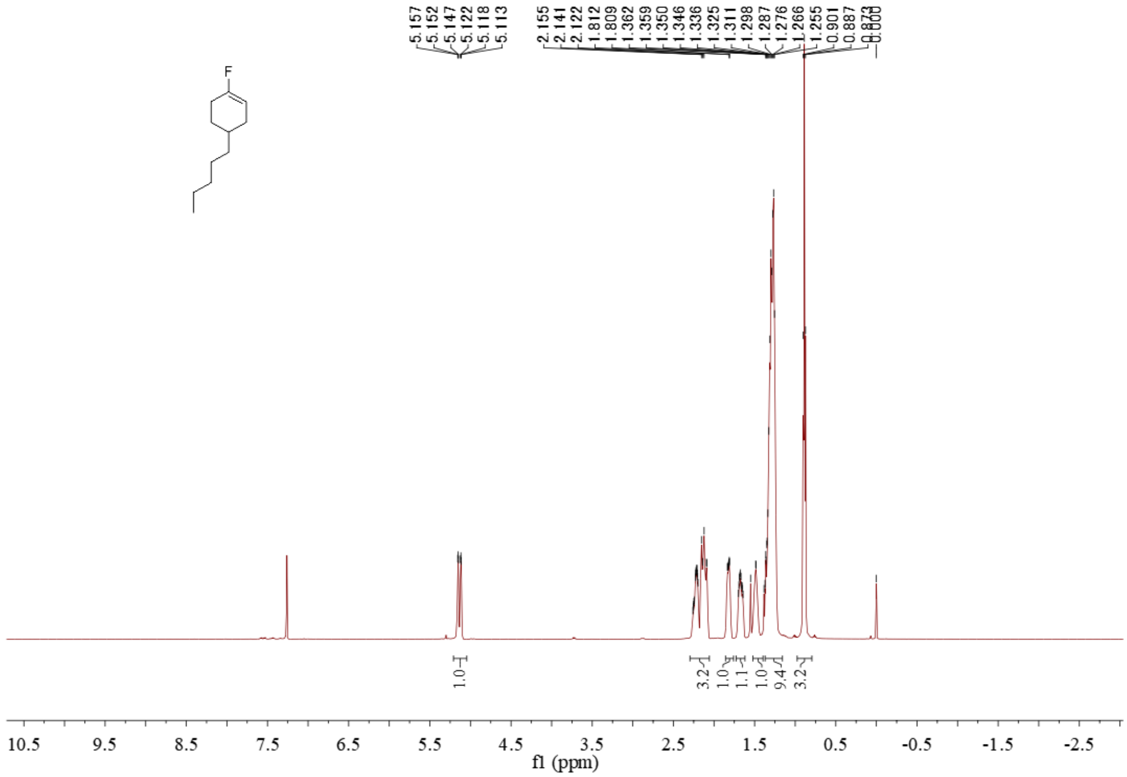

Supplementary Figure 70. ^{19}F NMR spectrum of known compounds **4u**, related to **Figure 4**

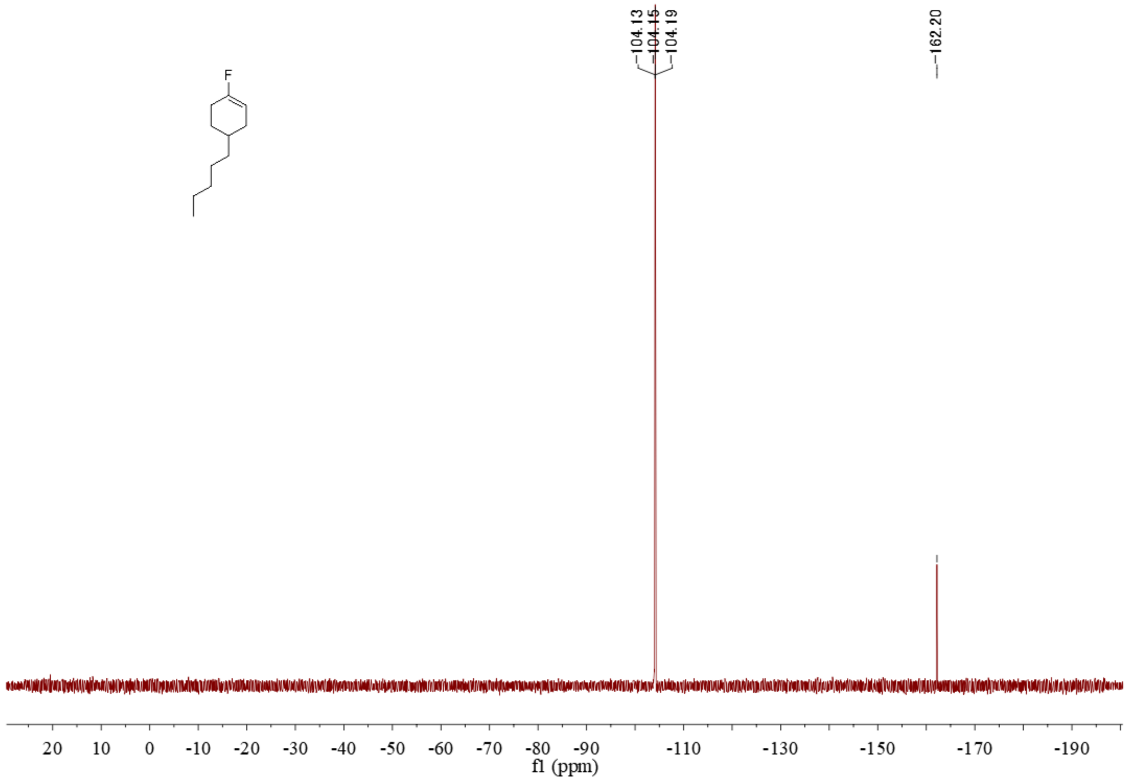

Supplementary Figure 71. ^{1}H NMR spectrum of known compounds **4w**, related to **Figure 4**

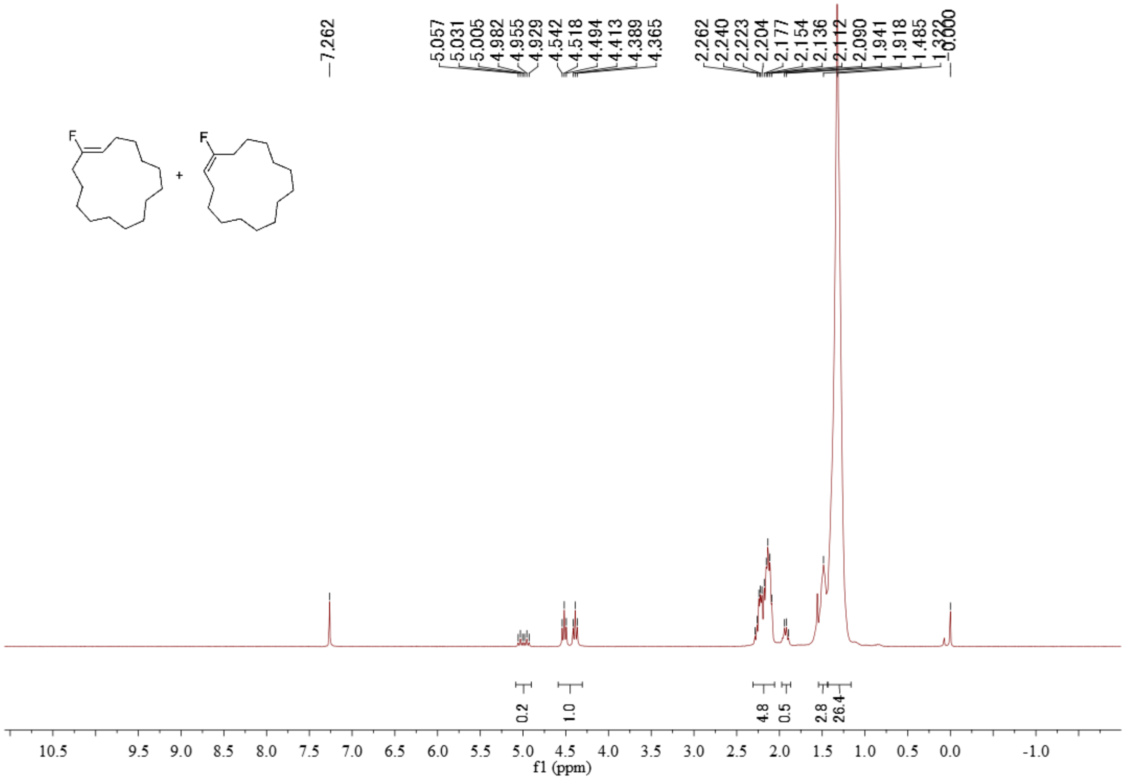

Supplementary Figure 72. ^{19}F NMR spectrum of known compounds **4w**, related to **Figure 4**

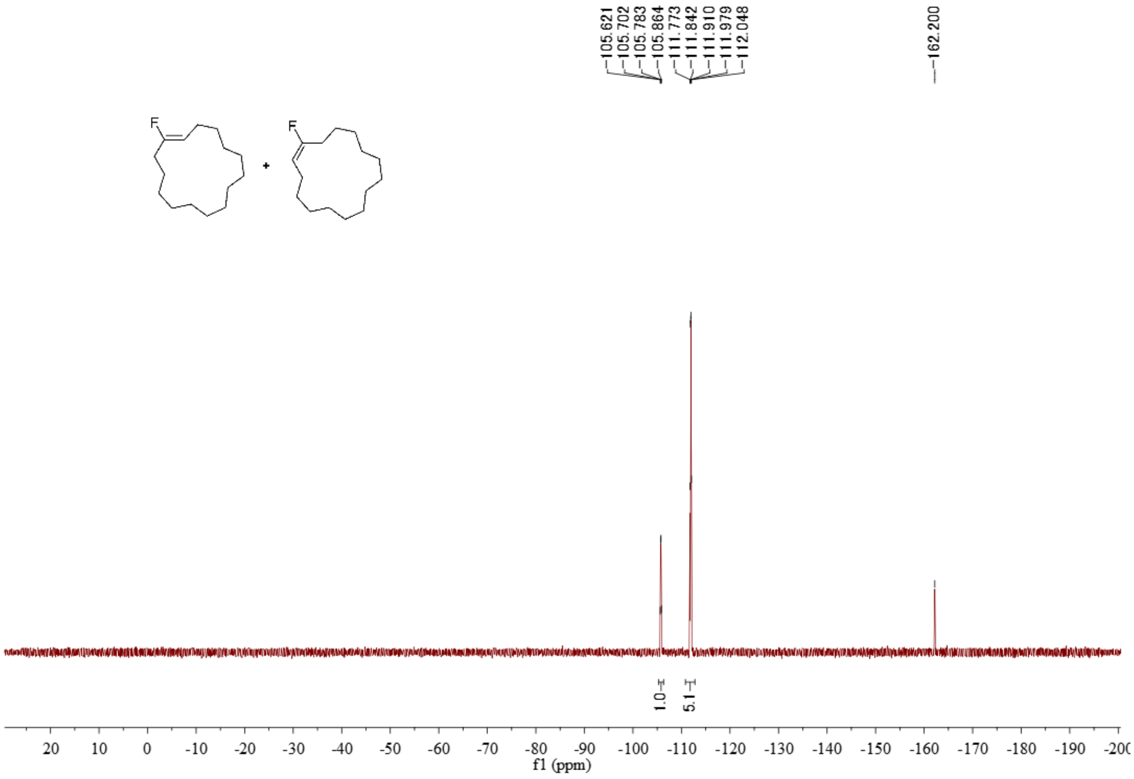

Supplementary Figure 73. ^{1}H NMR spectrum of known compounds **(*Z*)-4w**, related to **Figure 4**

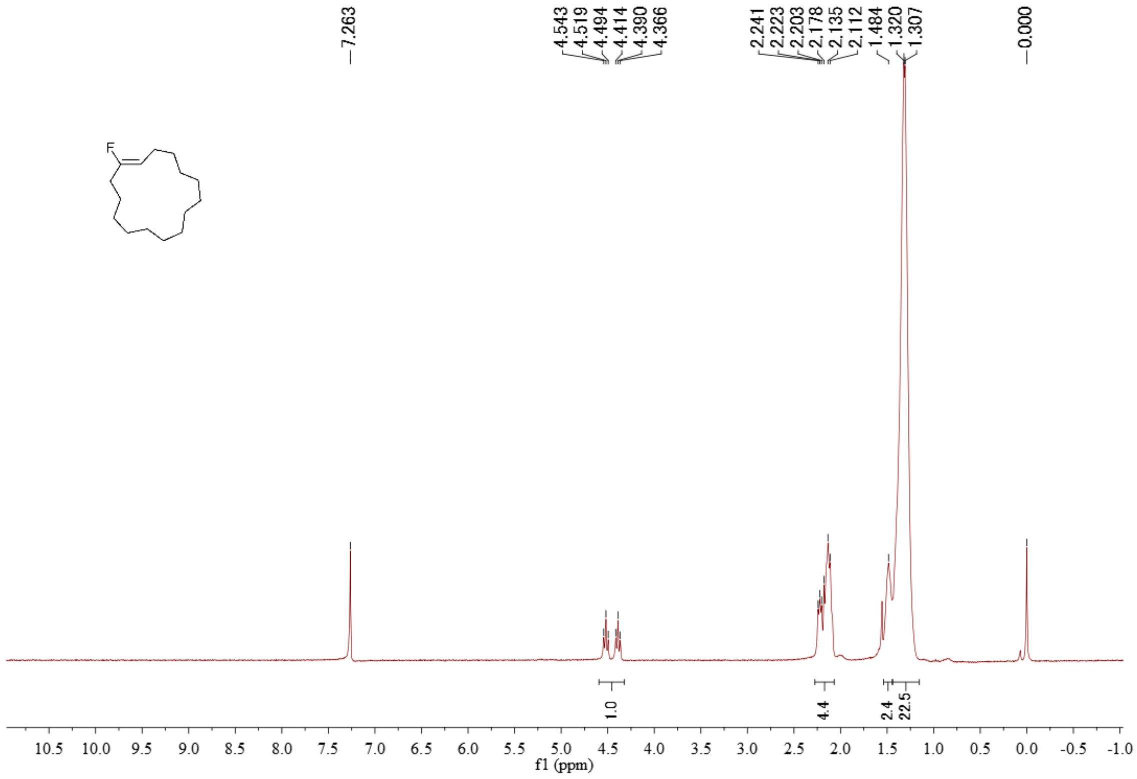

Supplementary Figure 74. ^{19}F NMR spectrum of known compounds **(*Z*)-4w**, related to **Figure 4**

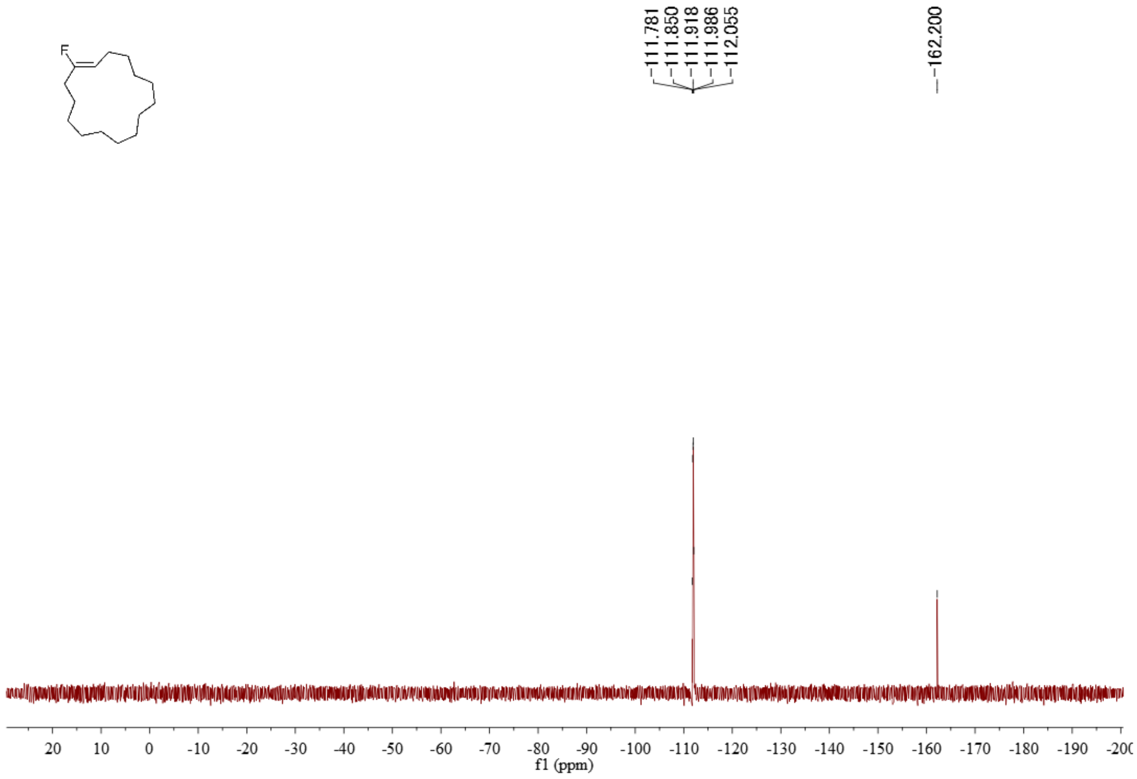

Supplementary Figure 75. ^{1}H NMR spectrum of crude reaction mixture under optimized reaction condition, related to **Table 1** (entry 1)

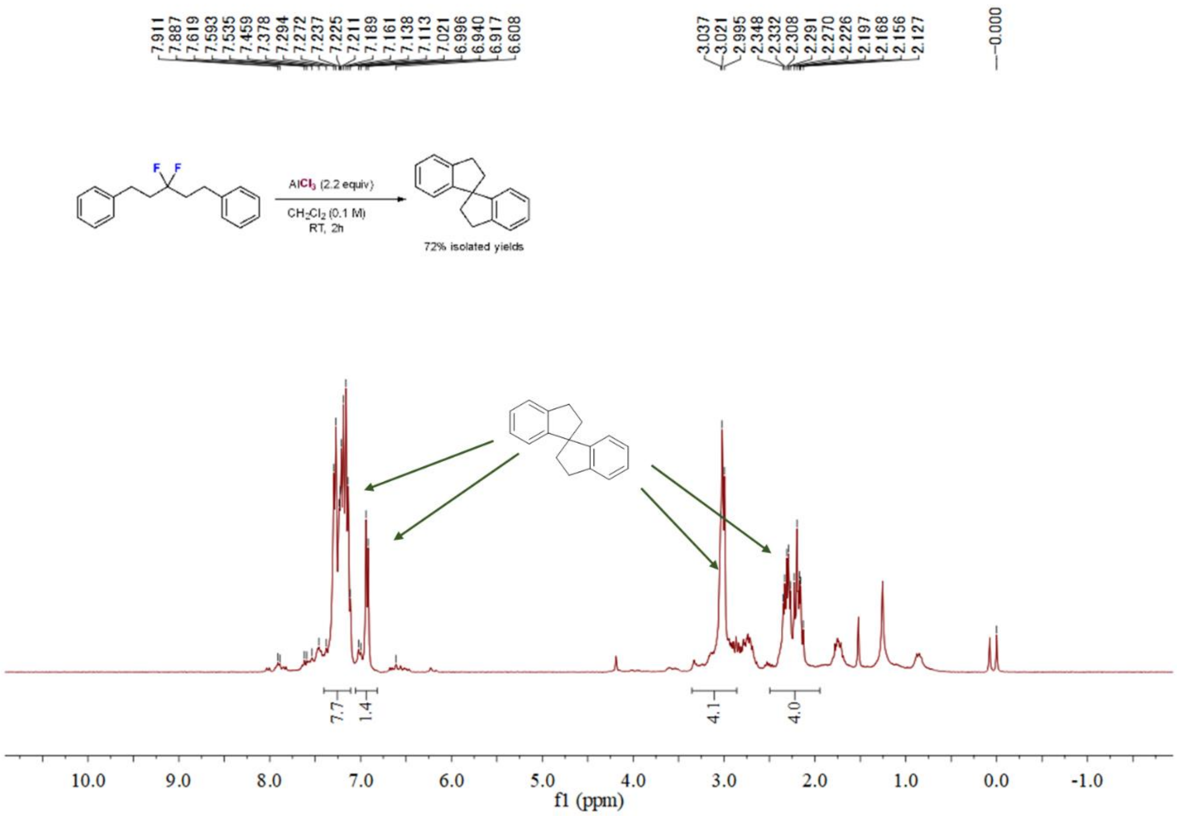

Supplementary Figure 76. ^{1}H NMR spectrum of crude reaction mixture under optimized reaction condition, related to **Table 1** (entry 5)

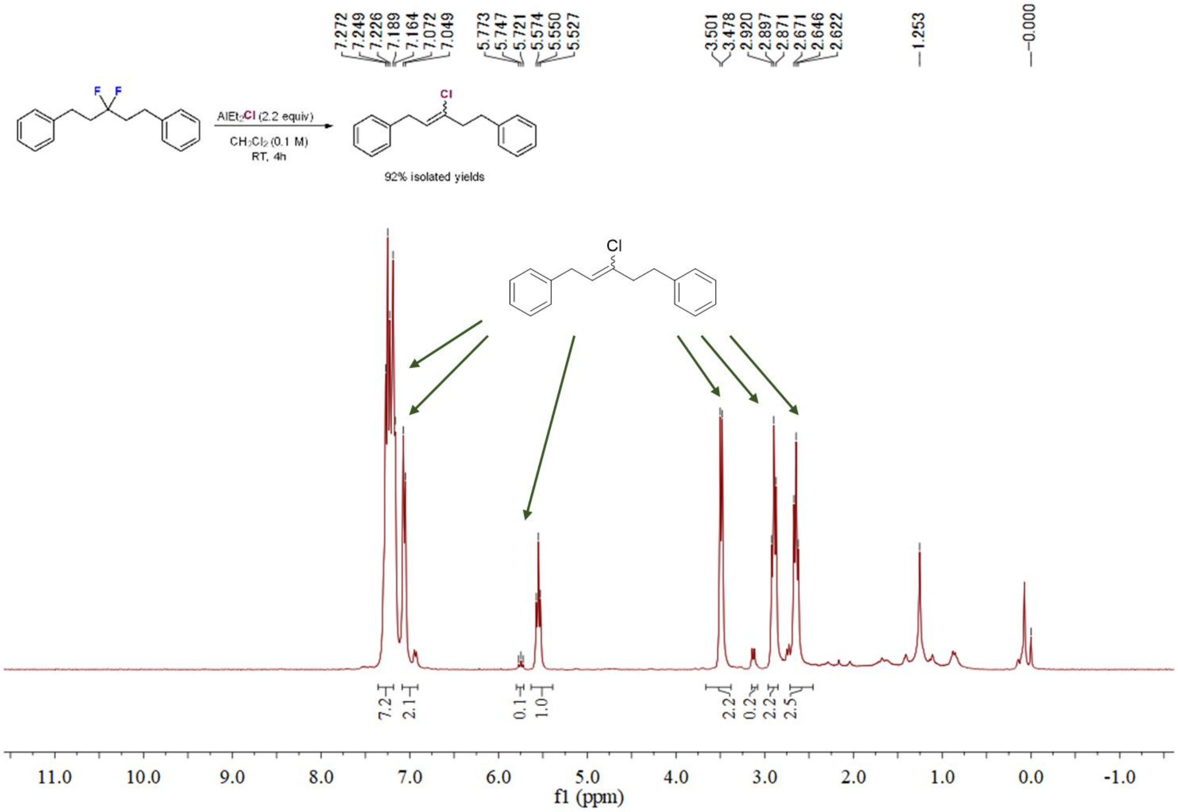

Supplementary Figure 77. ^{1}H NMR spectrum of crude reaction mixture under optimized reaction condition, related to **Table 1** (entry 7)

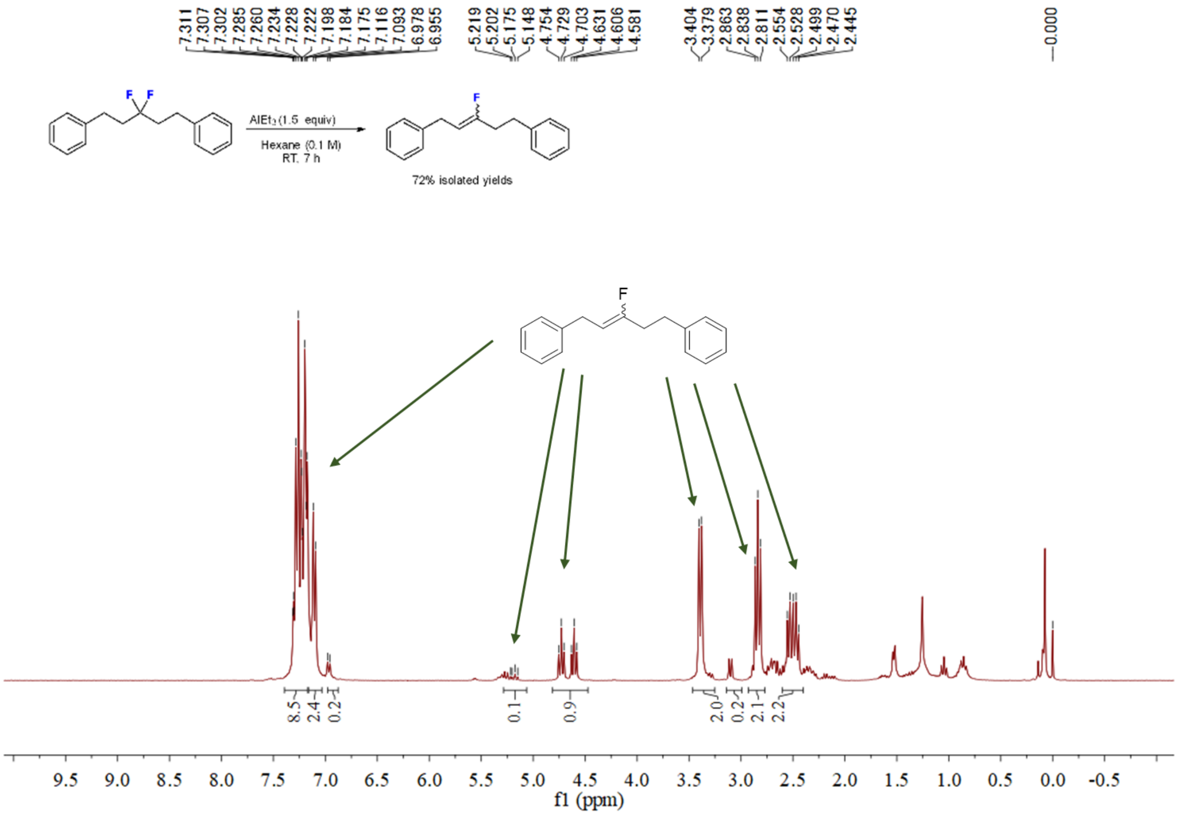

Supplementary Figure 78. ^{1}H NMR spectrum of control experiments, related to **Figure 5** (1)

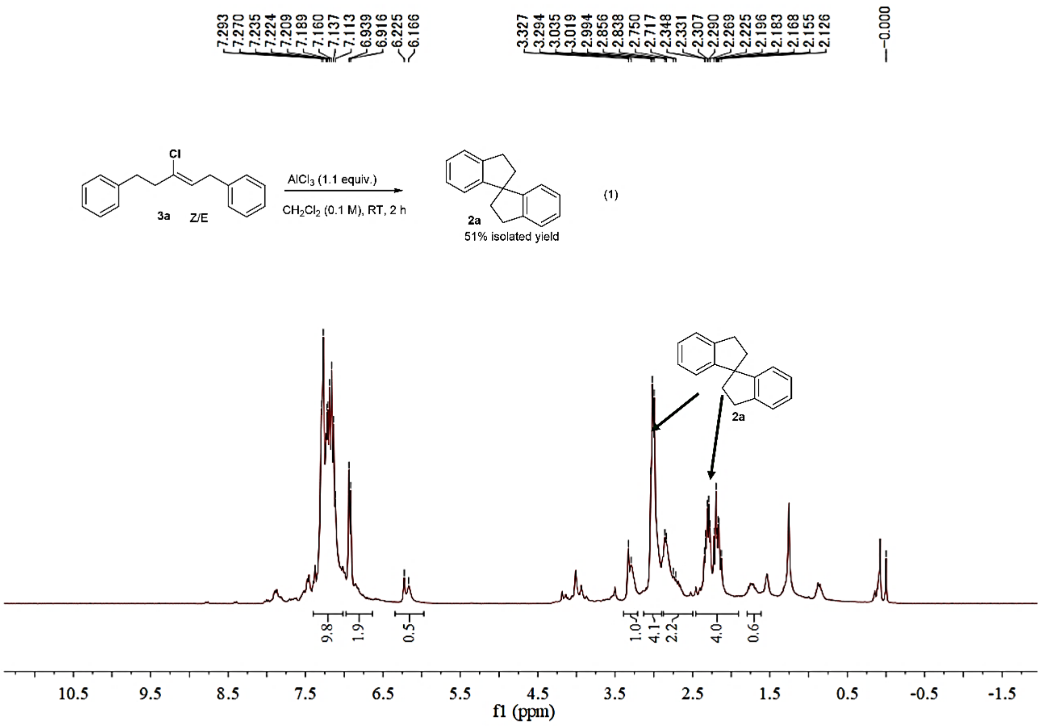

Supplementary Figure 79. ^{1}H NMR spectrum of control experiments, related to **Figure 5** (2)

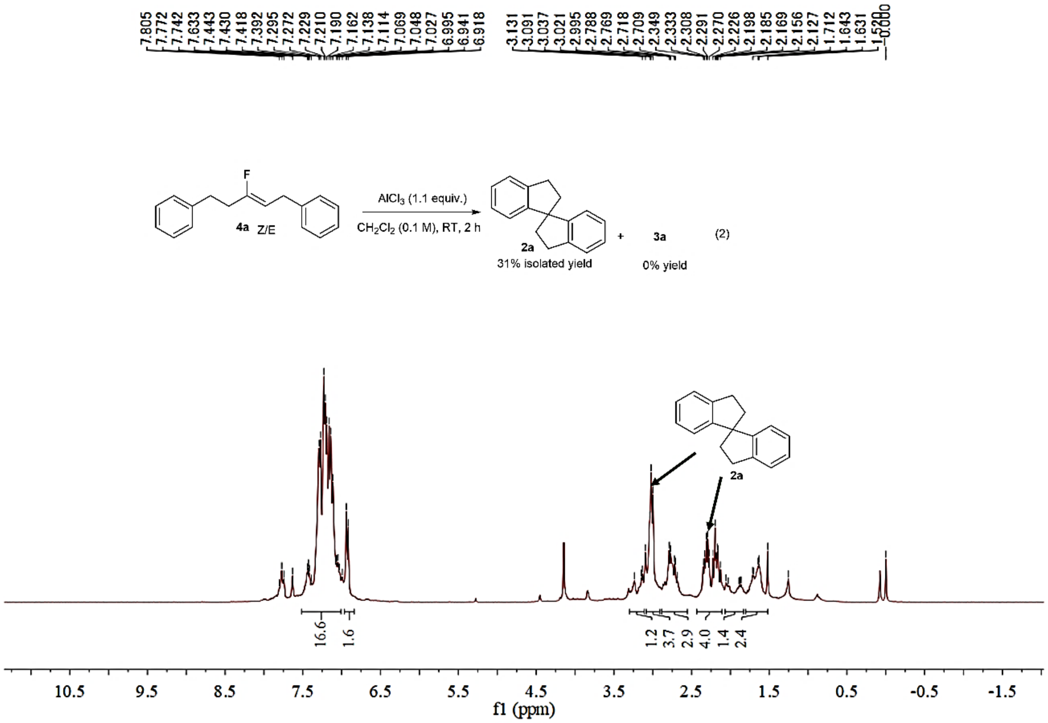

Supplementary Figure 80. ^{1}H NMR spectrum of control experiments, related to **Figure 5** (3)

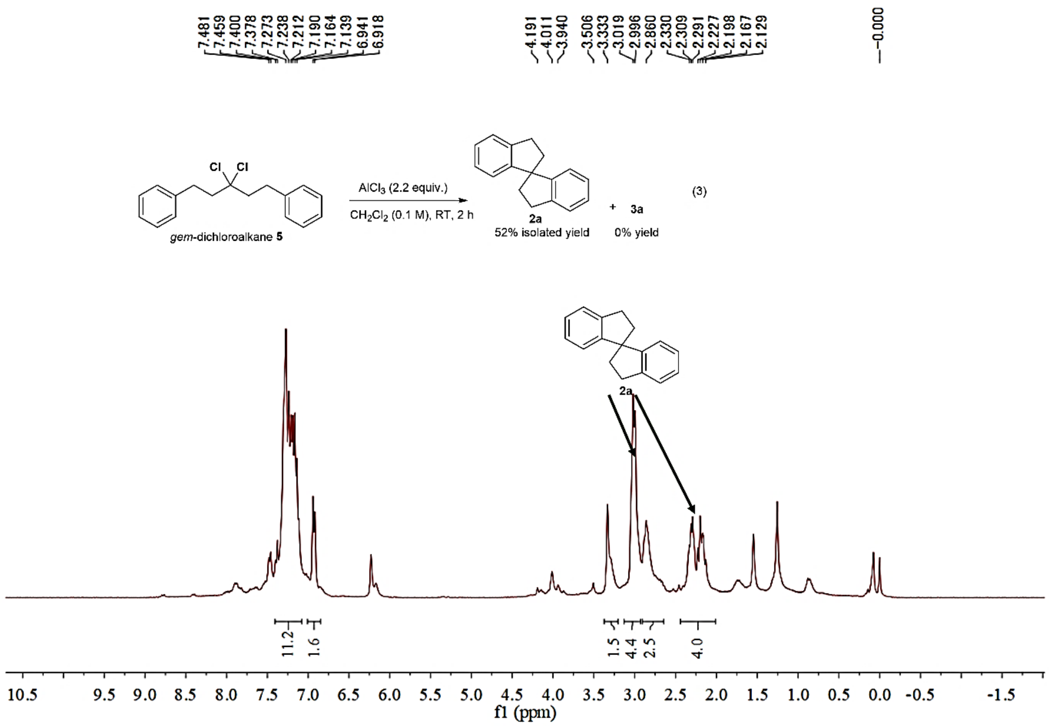

Supplementary Figure 81. ^{1}H NMR spectrum of control experiments, related to **Figure 5** (4)

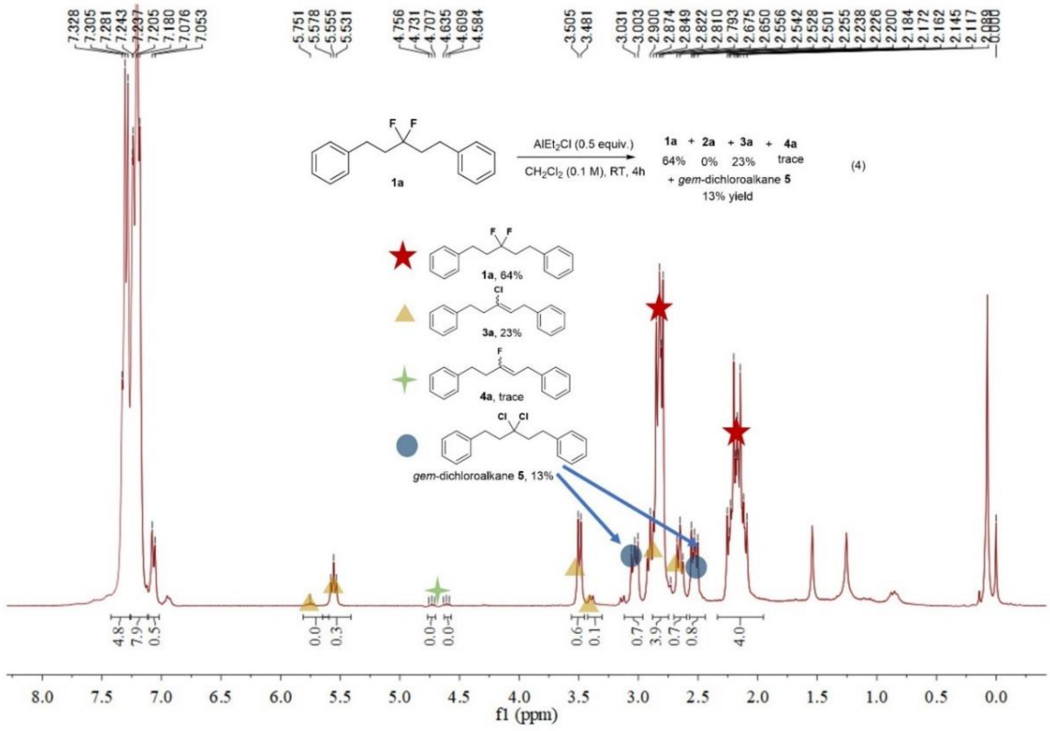

Supplementary Figure 82. ^{19}F NMR spectrum of control experiments, related to **Figure 5** (4)

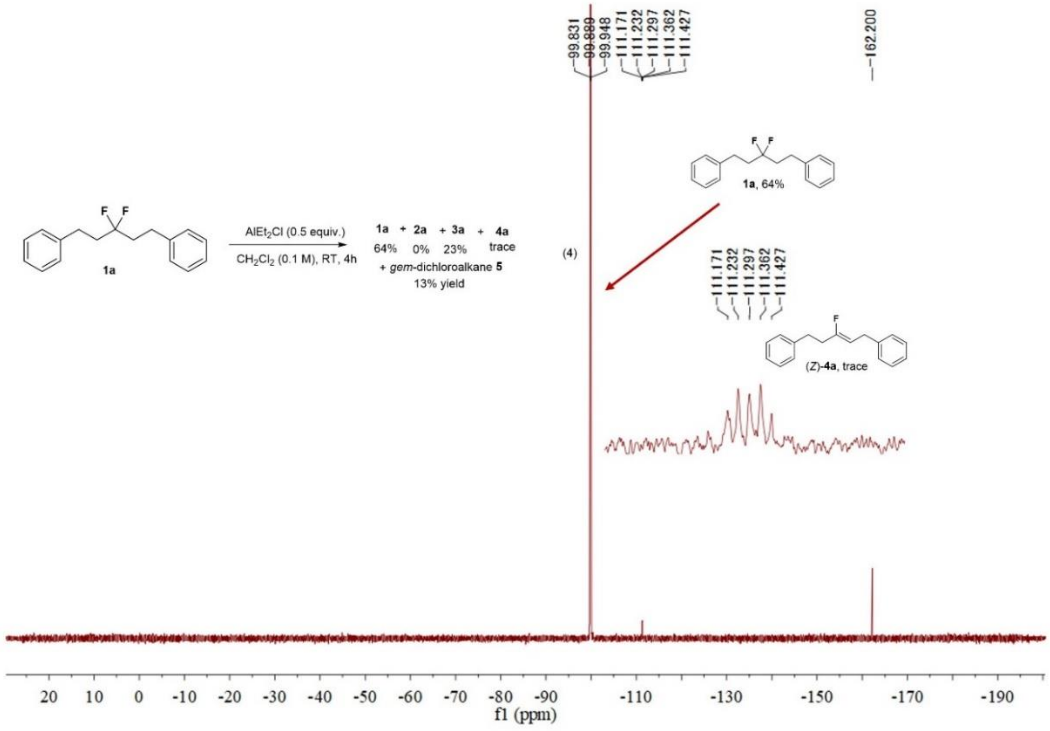

Supplementary Figure 83. GC-MS (EI) analysis of crude reaction mixture of control experiments, related to **Figure 5** (4)

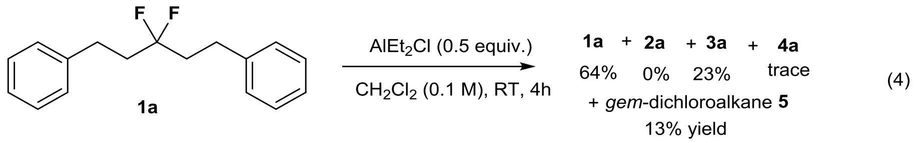

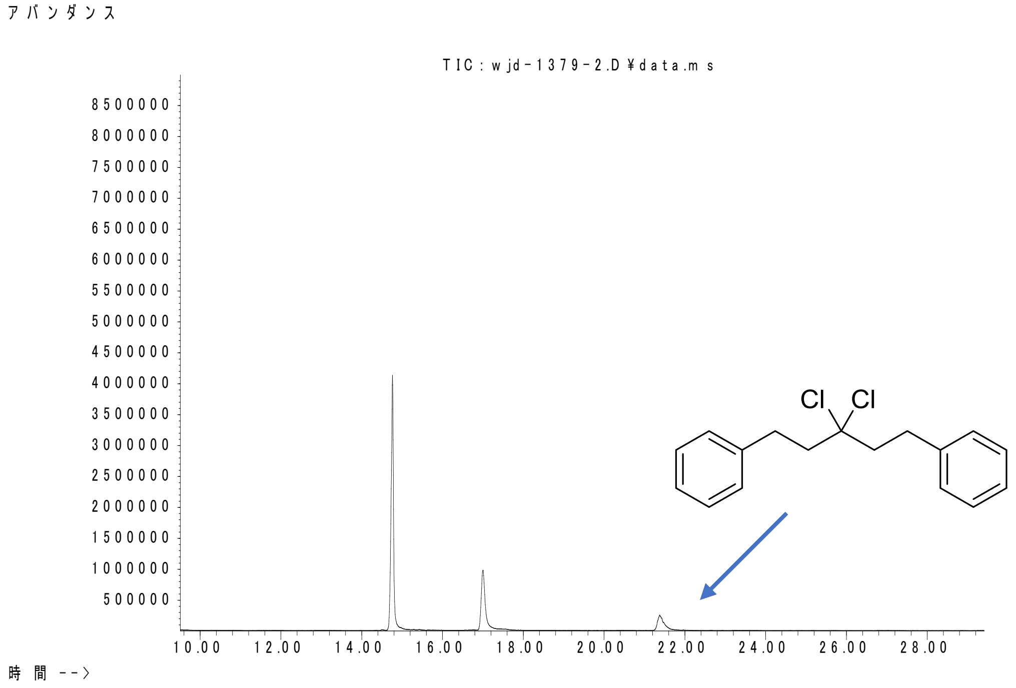

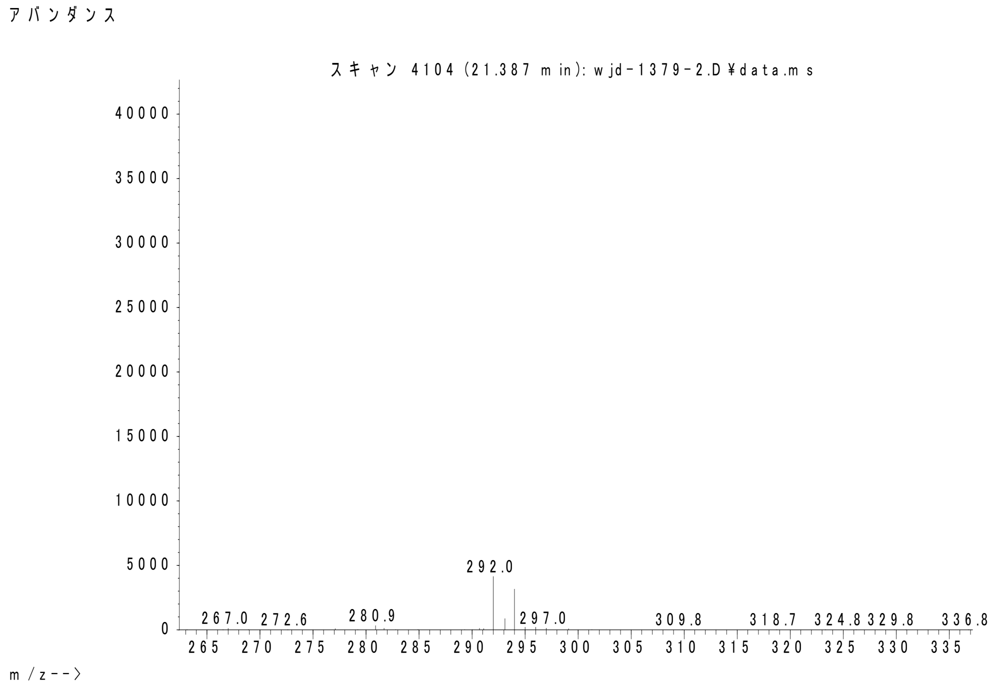

Supplementary Figure 84. ^{1}H NMR spectrum of control experiments, related to **Figure 5** (5)

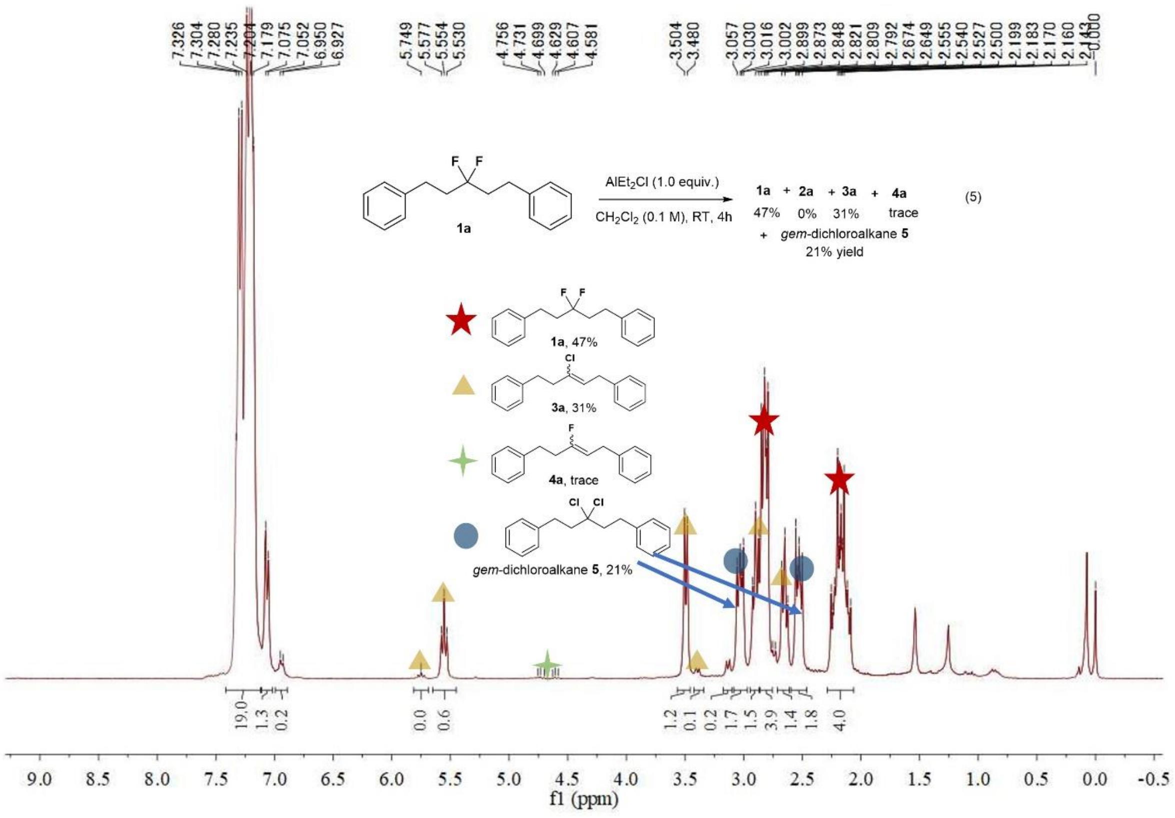

Supplementary Figure 85. GC-MS (EI) analysis of crude reaction mixture of control experiments, related to **Figure 5** (5)

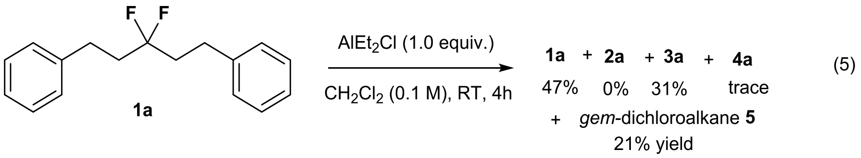

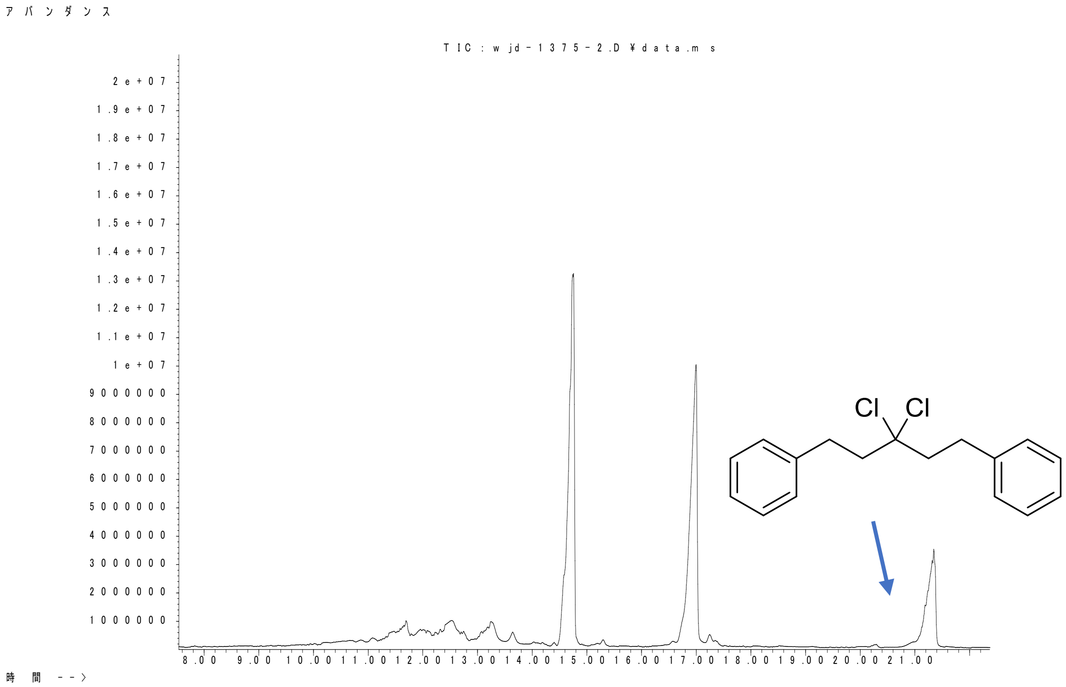

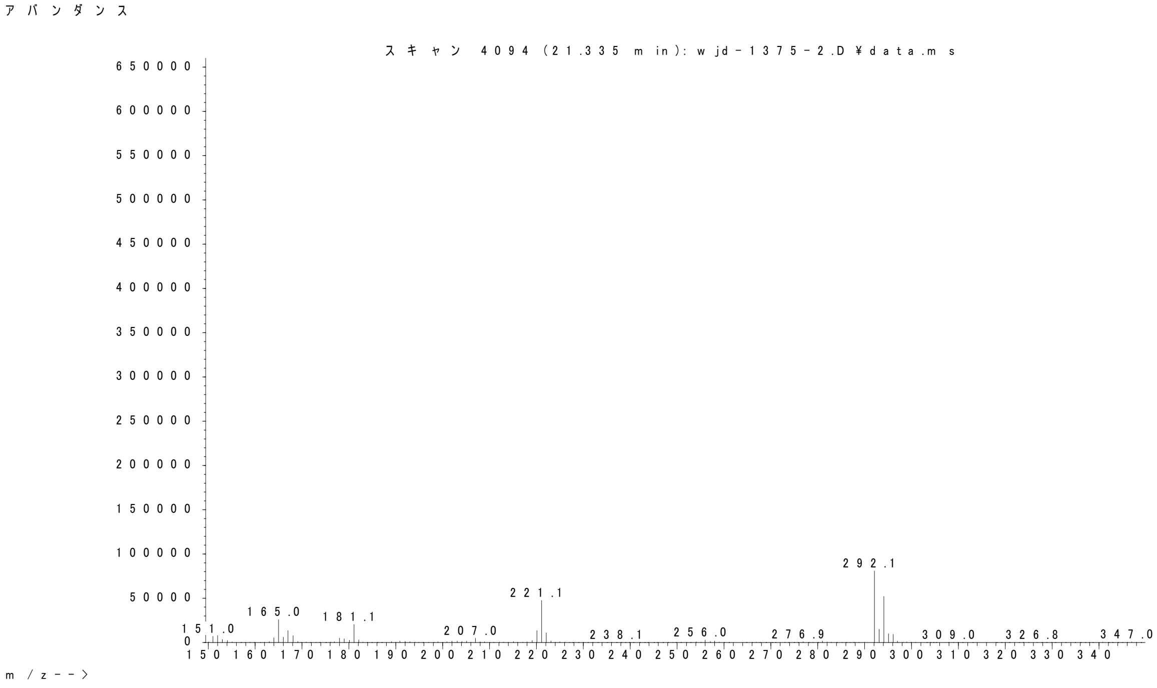

Supplement: Supplementary file 1 — supplementary information [file 41598_2019_55206_MOESM1_ESM.pdf]
